# Loss of Zbtb32 in NOD mice does not significantly alter T cell responses.

**DOI:** 10.12688/f1000research.13864.2

**Published:** 2018-11-05

**Authors:** William D. Coley, Yongge Zhao, Charles J. Benck, Yi Liu, Chie Hotta-Iwamura, M. Jubayer Rahman, Kristin V Tarbell

**Affiliations:** 1 Immune Tolerance Section, National Institute of Diabetes and Digestive and Kidney Diseases, National Institutes of Health, Bethesda , MD, 20892, USA; 2 Department of Inflammation and Oncology, Amgen, Inc, South San Francisco, CA, USA

**Keywords:** Zbtb32, ROG, NOD, diabetes, CRISPR, CRISPR/Cas9

## Abstract

**Background** *: *We previously identified the transcriptional regulator Zbtb32 as a factor that can promote T cell tolerance in the Non-Obese Diabetic (NOD) mouse, a model of Type 1 diabetes. Antigen targeted to DCIR2 ^+^ dendritic cells (DCs) *in vivo *inhibited both diabetes and effector T cell expansion in NOD mice. Furthermore, Zbtb32 was preferentially induced in autoreactive CD4 T cells stimulated by these tolerogenic DCIR2 ^+^ DCs, and overexpression of Zbtb32 in islet-specific T cells inhibited the diabetes development by limiting T cell proliferation and cytokine production.

**Methods** *:* To further understand the role of Zbtb32 in T cell tolerance induction, we have now used CRISPR to target the Zbtb32 gene for deletion directly in NOD mice and characterized the mutant mice. We hypothesized that the systemic loss of Zbtb32 in NOD mice would lead to increased T cell activation and increased diabetes pathogenesis.

**Results** *:* Although NOD.Zbtb32 ^-/- ^male NOD mice showed a trend towards increased diabetes incidence compared to littermate controls, the difference was not significant. Furthermore, no significant alteration in lymphocyte number or function was observed. Importantly, *in vitro* stimulation of lymphocytes from NOD.Zbtb32 ^-/- ^mice did not produce the expected hypersensitive phenotype observed in other genetic strains, potentially due to compensation by homologous genes.

**Conclusions** *:* The loss of Zbtb32 in the NOD background does not result in the expected T cell activation phenotype.

## Introduction

Although some autoimmune diseases can be managed by the use of immunosuppressive drugs, current treatment options for individuals with Type 1 diabetes (T1D) are largely limited to controlling the disease symptoms instead of addressing the underlying autoimmune assault ^1^. Reestablishing immune tolerance to the targeted self-antigens could provide more specific and durable treatment options ^2^. The Non-Obese Diabetic (NOD) mouse strain spontaneously develops autoimmune diabetes, and can be used to identify how tolerance is defective in chronic autoimmune environments or to find potential therapies that can overcome these defects ^3, 4^.

 Previously, we targeted self antigens to specific dendritic cell (DC) subsets by using chimeric antibodies that recognize antigen uptake receptors (either DEC205 for cDC1s ^5^ or DCIR2 for cDC2s) with the relevant self peptide covalently attached ^6^. The entire antibody is internalized and the antigenic self peptide is then processed and presented on MHC class II. This allowed identification of DC subsets able to induce tolerance against β-islet antigens within the context of chronic autoimmunity found in NOD mice. Using this approach, we demonstrated that self-antigen presentation by DCIR2 ^+^ DCs but not DEC205 ^+^ DCs could promote self-tolerance via increased apoptosis and decreased effector functions in islet-specific CD4 T cells ^6^. A gene expression analysis of the affected autoreactive T cells showed increased early expression of Zbtb32 in T cells stimulated by DCIR2 ^+^ DCs. In addition, transient overexpression of Zbtb32 in islet-specific CD4 T cells delayed diabetes development, and decreased both proliferation and IFNγ production in islet-specific CD4 ^+^ T cells ^6^. Together these studies suggest T cell expression of Zbtb32 plays a role in tolerance induction in NOD mice, and could be a target for treatment.

The Zbtb32 gene (also known as ROG or PLZP) was first identified as a homologue of the leukemia oncogene PLZF (Zbtb16) ^7^. Zbtb32 was then shown to act as a transcriptional repressor of GATA-3 ^8, 9^. Since GATA-3 regulates thymocyte development and T _H_2 differentiation, Zbtb32 knockout mice were produced to test if Zbtb32 played a role in modulating lymphocyte development and response to stimuli. In three genetic backgrounds (129/SV, C57Bl6, and BALB/c), the homozygous loss of Zbtb32 significantly altered T cell responses resulting in increased proliferation, increased cytokine production, and hypersensitive responses to inflammatory stimuli ^10– 13^. NK cells and B cells from C57Bl6 mice lacking Zbtb32 similarly displayed hyperproliferation and increased effector functions ^14, 15^. Therefore, data from these knockout strains show that Zbtb32 functions as a brake for lymphocyte activation and differentiation, and is consistent with our data from transient overexpression of Zbtb32 in T cells.

To better understand the role of Zbtb32 for T cell tolerance in autoimmune NOD mice, we introduced a targeted deletion using CRISPR/CAS9 techniques. The creation of mutant NOD strains has been severely limited by the lack of a robust ES line, necessitating backcrossing knockouts made in other genetic backgrounds onto the NOD background ^16, 17^. Because the region surrounding the knockout locus will retain alleles from the original strain, this can confound interpretation of such mice. NOD mice carry dozens of genetic susceptibility loci that contribute to the onset of T1D and some of those linked alleles could alter diabetes susceptibility or immune phenotype of any new mutant strain ^18^. These issues can be avoided by using CRISPR/CAS9 technology to directly edit DNA in NOD embryos, an approach utilized only very recently in the NOD strain ^19^. For the present study, we deleted a portion of exon 2 to cause a frameshift mutation in the Zbtb32 gene, and validated the loss of the Zbtb32 protein in our colony of NOD.Zbtb32 ^-/-^ mice. These mice were used to test our hypothesis that the transcription repressor Zbtb32 plays a critical role in the inhibition of T-cell mediated autoimmune T1D. Surprisingly, T cells from NOD.Zbtb32 ^-/-^ mice were not hyperreactive as would have been expected based on both our prior overexpression data and knockout mice in other strains. Although male NOD.Zbtb32 ^-/-^ mice showed a trend of increased diabetes incidence compared to littermate controls, the difference was not significant, and no significant difference in either time of onset or overall incidence was observed in female NOD.Zbtb32 ^-/-^ mice. Overall, most of our experiments suggested mild phenotypic changes perhaps as a result of either compensation by other family members or by effects on multiple cell types that is different on the NOD genetic background.

## Methods

### Mice

Non-Obese Diabetic mice (NOD, JAX #001976) were initially obtained from The Jackson Laboratory, Bar Harbor ME, and bred in the facility at NIH. Additional NOD females were regularly delivered every two weeks to compensate for the loss of breeders due to diabetes in the wildtype NOD colony. Approximately 630 NOD.Zbtb32 ^-/-^ mice (about 90 litters with an average liter size of 7) were bred during the characterization the mutant strain. All mice were housed in a specific pathogen free vivarium with a 12 h on/12 h off light cycle (6 am on and 6 pm off). The care, use, and disposition of all mice used in this study were reviewed and approved (protocol K024-DEOB-16) by the Institutional Animal Care and Use Committee of the NIDDK, NIH. Diabetes was monitored and mice were considered diabetic on the first of two blood glucose level measurements above 250 mg/dl.

### Generation of knockout

**Figure 1.**
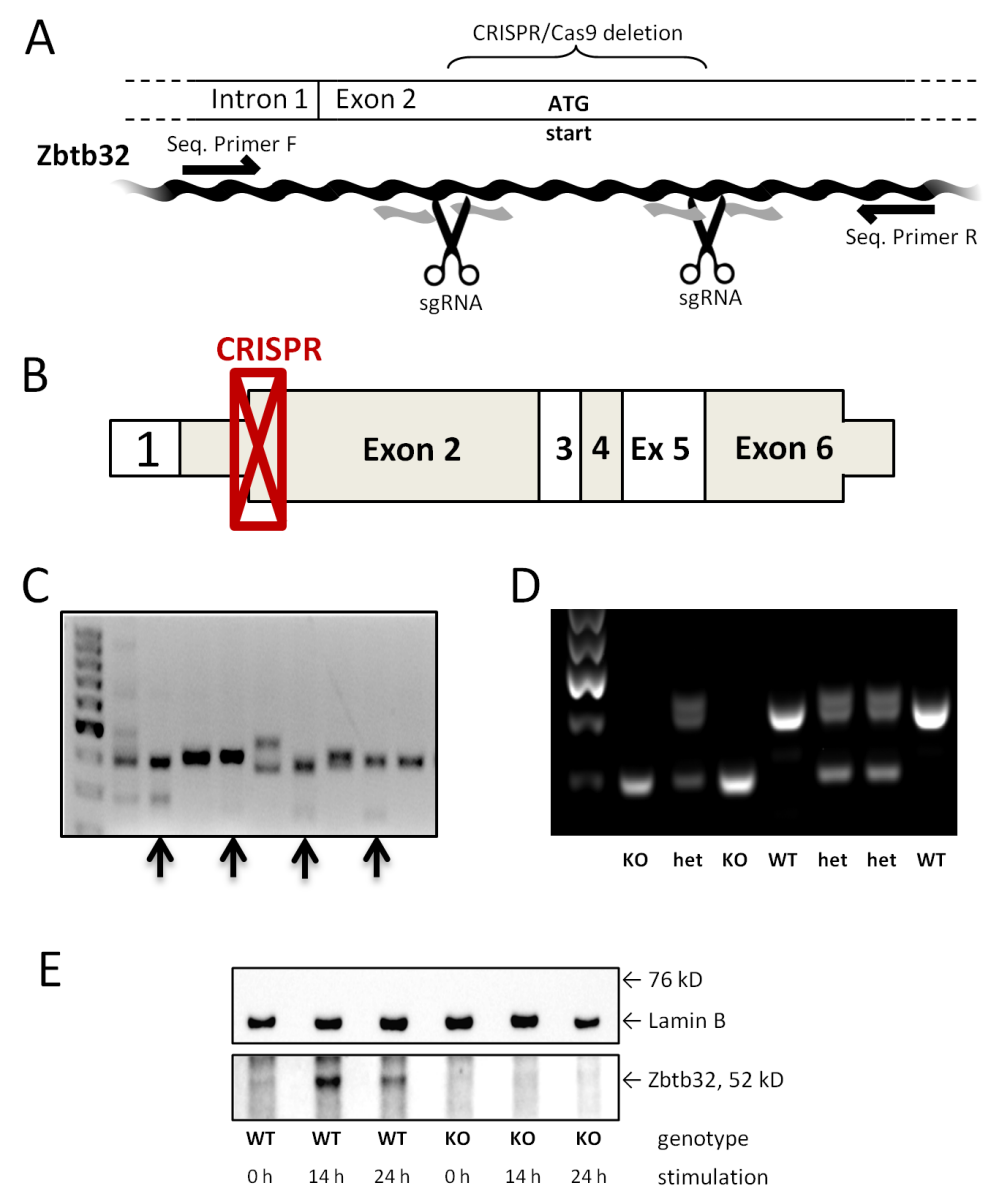
Confirmation of CRISPR-mediated knockout of Zbtb32. The CRISPR/Cas9 technique was used to target exon 2 of the Zbtb32 gene directly in NOD mice. A portion of the Zbtb32 gene was deleted and a frameshift introduced in NOD mice using the CRISPR/Cas9 technique, as illustrated here ( **A**). The scissors represent the location of cutting based on sgRNA binding and black arrows indicate binding sites for the standard primers, which were used for both sequencing and genotyping. The location of the deletion maps onto the beginning of exon2 of the wildtype mRNA ( **B**). Non-coding portions are shown as thinner than the coding portions. The CRISPR/Cas9-treated embryos develop into mice with genetic mosaicism of the Zbtb32 gene in the F0 generation. Four genetically heterogeneous females (indicated with black arrows) were examined further before one mutant allele was chosen to establish the NOD.Zbtb32 ^-/-^ colony ( **C**). The targeted deletion of Zbtb32 removed 146 bp from exon 2 as shown by genetic screening of knockout, heterozygous, and wildtype animals in the F2 generation ( **D**). The loss of the Zbtb32 protein after CRISPR-mediated deletion was confirmed by Western blotting extracts from stimulated CD4 ^+^ splenocytes ( **E**) (from three biologically independent replicates).

The NOD.Zbtb32 ^-/-^ genetic alteration was carried out as follows: NOD female mice (6–8 weeks old) were super ovulated and mated overnight with NOD male mice (>8 weeks old). Zygotes were harvested from the ampullae of super ovulated females and were placed in KSOM medium (Millipore, Billerica MA) before microinjection. Microinjection was performed in M2 medium (Sigma, St Louis, MO) using a micromanipulator (Narishige) and microscope (Nikon). The second exon of the Zbtb32 gene was targeted using the two following RNA guide sequences; 5'-UACAG UUAGC GGCUA GACUC-3' and 5'-CAAUC AUGGA UCCCC CAUUG-3'. Binding sites for single guide RNA (sg RNA) are indicated in Figure 1A. The modified Cas9n enzyme with nickase activity (System Biosciences) and sgRNA were co-injected into the pronucleus and cytoplasm of 238 zygotes (1 ^st^ round 37, 2 ^nd^ round 47, and 3 ^rd^ round 154) and resulted in a total of 35 pups. The final injection concentration in the mixture was 10 ng/μl Cas9n and 5 ng/μl of each sgRNA. After injection and incubation in 5.5% CO _2_ at 37°C overnight for 24 h, surviving 2-cell stage embryos were transferred to female NOD pseudopregnant recipients via oviduct transfer.

### Characterization of CRISPR/CAS9n genetic alterations

Genomic DNA was extracted from tail snips of the 35 21 day-old F0 mice using the DNeasy blood and tissue kit (Qiagen). The target region was PCR amplified by PuReTaq Ready-To-Go PCR beads (GE Healthcare) followed by IndelCheck CRISPR/TALEN insertion or deletion detection system (GeneCopoeia). Successful genetic editing of the Zbtb32 gene was observed in 15 of the 35 pups. Genomic DNA from pups with evidence of deletion was further PCR amplified and subsequently cloned by TOPO T/A subcloning (Invitrogen, Carlsbad, CA) followed by DNA sequencing. Compound heterozygous null founders were bred with wildtype NOD animals to provide four potential alleles (named A, B, C, and D) for the creation of a NOD.Zbtb32 ^-/-^ colony. All four mutant alleles were sequenced to verify a successful deletion ( Supplementary Table 1). As expected, the mutant alleles displayed a variety of deletion sizes due to differential DNA repair. Sibling crosses with matching mutant alleles were set up but, only mice carrying the mutant B allele were reliable breeders. Therefore, all NOD.Zbtb32 ^-/-^ mice described in this paper are homozygous for the B allele.

### Genotyping and sequencing

For routine genotyping and sequencing, we used the following PCR primers: forward 5’-AGCTG GCCTT TGGCT TAGTT-3’ and reverse 5’-CAAAG GTGGA AGGGC TTATG-3’. Binding sites for these primers in relation to the CRISPR/Cas9 deletion are indicated in Figure 1A. PCR conditions followed a typical 3-temp program; 95°C for 5 min, [95°C 30s, 55°C 30s, 72°C 45s] repeat 34 times, and finally 72°C for 10 min. The PCR results were run out on a 2% agarose gel. Single pass DNA sequencing services (ACGT, Inc., Germantown, MD) were carried out on genomic DNA using the reverse primer.

### Western blotting

During dissections, mice were euthanized by CO2 asphyxiation and then the spleen was removed and placed in ice-cold PBS. Splenocytes were negatively enriched for CD4 ^+^ T cells using magnetic beads via the mouse CD4 ^+^ T Cell Isolation Kit (Miltenyi Biotech, Bergisch Gladbach, Germany) and then were incubated with Armenian Hamster anti-mouse CD3 (clone 145-2C11, Biolegend cat#100301) and Syrian Hamster anti-mouse CD28 (clone 37.51, Biolegend cat#102111) for either 14 h or 24 h at 37°C in T-cell Media (RPMI-1640+10% FBS+L-glutamine +Penn/Strep). The cells were then pelleted and lysed used RIPA buffer supplemented with Protease Inhibitor Cocktail (Roche, Indianapolis, IN). A total of 35 µg of protein was loaded into a NuPAGE 4–12% Bis-Tris gel, transferred onto nitrocellulose, blocked in 5% milk for 1 hour at 25°C, and blotted for Zbtb32 using rabbit anti-mouse sc-25358 (Santa Cruz Biotech, Santa Cruz CA) at a 1:500 dilution overnight at 4°C. The membrane was then washed prior to the addition of goat-anti-rabbit HRP (Jackson Immunoresearch cat#111-035-003) at a dilution of 1:5,000 dilution at 1 hour at 25°C. The membrane was then stripped, reblocked and reblotted for Lamin B using goat anti-mouse sc-6217 at a 1:5,000 dilution for 1 hour at 25°C. The membrane was then washed prior to the addition of donkey anti-goat HRP (Jackson Immunoresearch cat#705-035-003) at a dilution of 1:5,000 dilution for 45 minutes at 25°C. All protein bands were imaged on a BioRad ChemiDoc CCD system and processed with the BioRad Image Lab 6.0 software.

### *Ex vivo* stimulations and cytokine staining

Splenocytes and lymphocytes were stimulated with anti-mouse CD3 (145-2C11; BioLegend, San Diego, CA). A total of 5×10 ^5^ cells were incubated for 18 hours at 37°C in T-cell media with either 0, 10, 100, or 1000 ng of anti-CD3 in a 300 µl volume. For cytokine staining, cells were stimulated with PMA and Ionomycin in T-cell Media for 5 hours at 37°C. Brefeldin A was added to all cells for the final 4 hours of stimulation. Cells were then fixed and stained for flow cytometry. FMO staining controls were performed using a mixture of both unstimulated and stimulated cells.

### Flow cytometry

All cells were washed in Wash Buffer [PBS+2% FBS] and then blocked with LEAF purified anti-CD16/32 (clone 93) on ice for 30 minutes prior to staining for flow cytometry. The Foxp3 (FJK-16s) antibodies were purchased from eBioscience (San Diego, CA). Foxp3 antibodies were diluted at 1:50 in Wash Buffer for cell staining, while all other antibodies were diluted at 1:100 in Wash Buffer. Staining was carried out for a minimum of 1 hour at 4°C. Antibodies purchased from Biolegend (San Diego, CA) are listed alongside their clone and Antibody Registry numbers. We used antibodies raised against mouse B220 (RA3-6B2, RRID:AB_11203907), CD1d (CD1.1, RRID:AB_2715919), CD4 (GK1.5, RRID:AB_312690), CD5 (53-7.3, RRID:AB_312736), CD11b (M1/70, RRID:AB_312798), CD8 (53-6.7, RRID:AB_2561352), CD19 (6D5, RRID:AB_130884), CD23 (B3B4, RRID:AB_10060129), CD25 (PC61, RRID:AB_2562611), CD43 (S11, RRID:AB_2563698), CD44 (IM7, RRID:AB_2621762), CD62L (MEL-14, RRID:AB_10555750), CD69 (H1.2F3, RRID:AB_10683447), CD335 (29A1.4, RRID:AB_10827686), IFNγ (XMG1.2, RRID:AB_469504). Cell viability was assessed by Fixable Live/Dead Aqua staining (Life Technologies, Grand Island, NY) according to the manufacturer’s protocol. Cell proliferation was measured using the Cell Trace Violet Kit (Life Technologies, Grand Island, NY) according to the manufacturer’s protocol. For cell fixation, permeabilization, and intracellular staining, we utilized the Intracellular Fixation & Permeabilization Buffer Set from eBiosciences. All samples were run on a BD Biosciences LSR-II cytometer and later analyzed using FlowJo v9.4.1. For analysis, CD44 expression was measured by mean fluorescence intensity (MFI) rather than by the percentage of positive expression.

### Statistical analysis

Statistical analysis for all experiments was carried out using GraphPad Prism v5. Where appropriate, statistical significance was calculated using either a Kaplan-Meier survival method (for disease incidence), or a two-way ANOVA tests for independent samples with *post hoc* Bonferroni comparisons for each possible pair of groups (used for all other data). Any significant differences ( *P* < 0.05) between the two control strains are denoted with an asterisk. All dot plots show exact data points with a horizontal line denoting the average. All histograms show the average and standard deviation for the data.

## Results

### Using CRISPR techniques to generate knockout mutations directly in the NOD genetic background

The NOD mouse is an important model for examining spontaneous and chronic autoimmune disease, but it has been difficult to obtain genetically modified NOD mice due to the lack of a robust NOD ES cell line ^19^. Directly targeting mutations into the NOD genetic background via CRISPR gene editing in NOD embryos is now possible ^20^. Matching sgRNAs in exon 2 of Zbtb32 ( Figure 1A and B) and CAS9n were injected into 2 cell-stage embryos. Using the CAS9n with nickase activity ensures that an insertion or deletion can only occur if CAS9n binds to both sgRNAs, vastly reducing off-target events ^21^. Four female mice carrying a suitable deletion were selected to be potential F0 founders (indicated in Figure 1C) and were crossed with wildtype NOD mice to produce heterozygous mice in the F1 generation. Two of these potential F0 founders were found to be germline compound heterozygous for the loss of Zbtb32, providing up to four potential alleles (listed in Supplementary Table 1 as alleles A, B, C, and D) for the creation of a NOD.Zbtb32 ^-/-^ colony. Sibling crosses with matching mutant alleles were set up using the F1 generation. Because mice carrying the mutant B allele were the most reliable breeders, all NOD.Zbtb32 ^-/-^ mice described in this paper are homozygous for the B allele containing a 146 bp frameshift deletion.

 We designed PCR primers to serve for both routine genotyping purposes (demonstrated in Figure 1D) and as sequencing primers. Genomic sequencing on select NOD.Zbtb32 ^-/-^ mice from multiple generations verified that the deletion within the Zbtb32 gene was stable ( Supplementary Figure 1). In wildtype NOD mice, Zbtb32 was not detectable at baseline in resting CD4+ lymphocytes, but could be detected by Western blot as early as 14 hours post anti-CD3/CD28 stimulation ( Figure 1E). No Zbtb32 protein was observed in NOD.Zbtb32 ^-/-^ mice at either baseline or post-stimulation ( Figure 1E).

Raw images for Figure 1 The raw images for the genotyping gels and western blots seen in Figure 1. Click here for additional data file. Copyright: © 2018 Coley WD et al. 2018 Data associated with the article are available under the terms of the Creative Commons Zero "No rights reserved" data waiver (CC0 1.0 Public domain dedication).

### Diabetes incidence in NOD.Zbtb32 ^-/-^ mice

**Figure 2.**
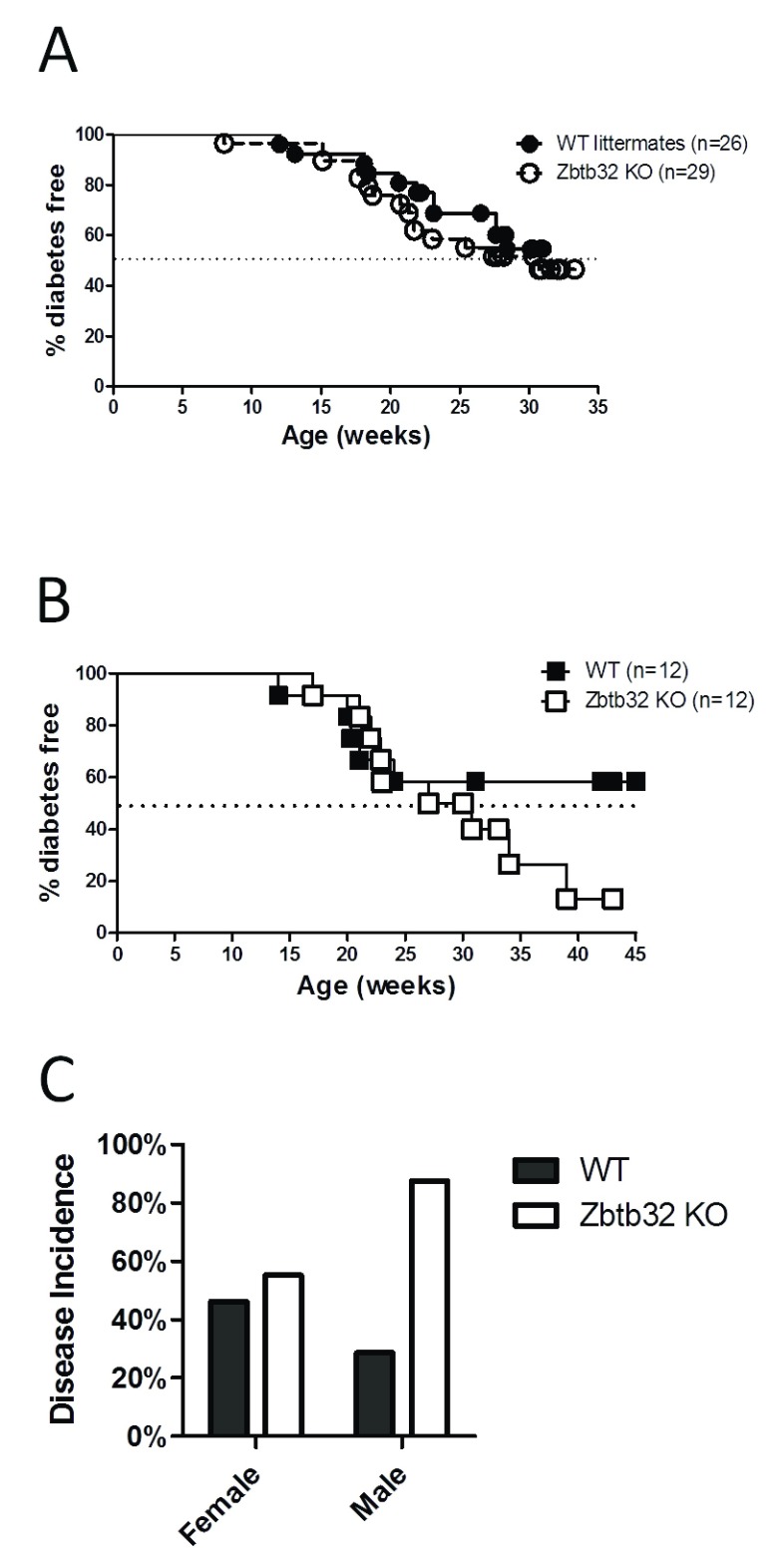
Diabetes incidence in NOD.Zbtb32 ^-/-^ mice. The onset of Type 1 diabetes was monitored in our NOD.Zbtb32 ^-/-^ colony by weekly blood glucose monitoring for both female ( **A**) and male ( **B**) mice. Disease incidence rates were followed for littermate wildtype, and homozygous knockout mice. Disease incidence rates for males and females were plotted in ( **C**).

To test the hypothesis that absence of Zbtb32 would increase diabetes pathogenesis in NOD mice, we monitored diabetes via blood glucose levels in NOD.Zbtb32 ^-/-^ mice and their control littermates for up to 35 weeks for both female ( Figure 2A) and male mice ( Figure 2B). NOD mice normally spontaneously develop hyperglycemia, between 12 and 30 weeks of age due to autoimmune destruction of the insulin secreting β-islet cells within the pancreas. The diabetes incidence rates showed no statistical differences between NOD.Zbtb32 ^-/-^ mice and their wildtype littermate controls when using a standard Kaplan-Meir survival test, but the male NOD.Zbtb32 ^-/-^ trended toward a higher than expected total diabetes incidence ( Figure 2C). Therefore, the trend in higher male disease incidence may indicate a mild phenotype in NOD.Zbtb32 ^-/-^ mice may impact disease onset over long timescales.

### *Ex vivo* T cell numbers and activation levels are not significantly altered in NOD.Zbtb32 ^-/-^ mice

**Figure 3.**
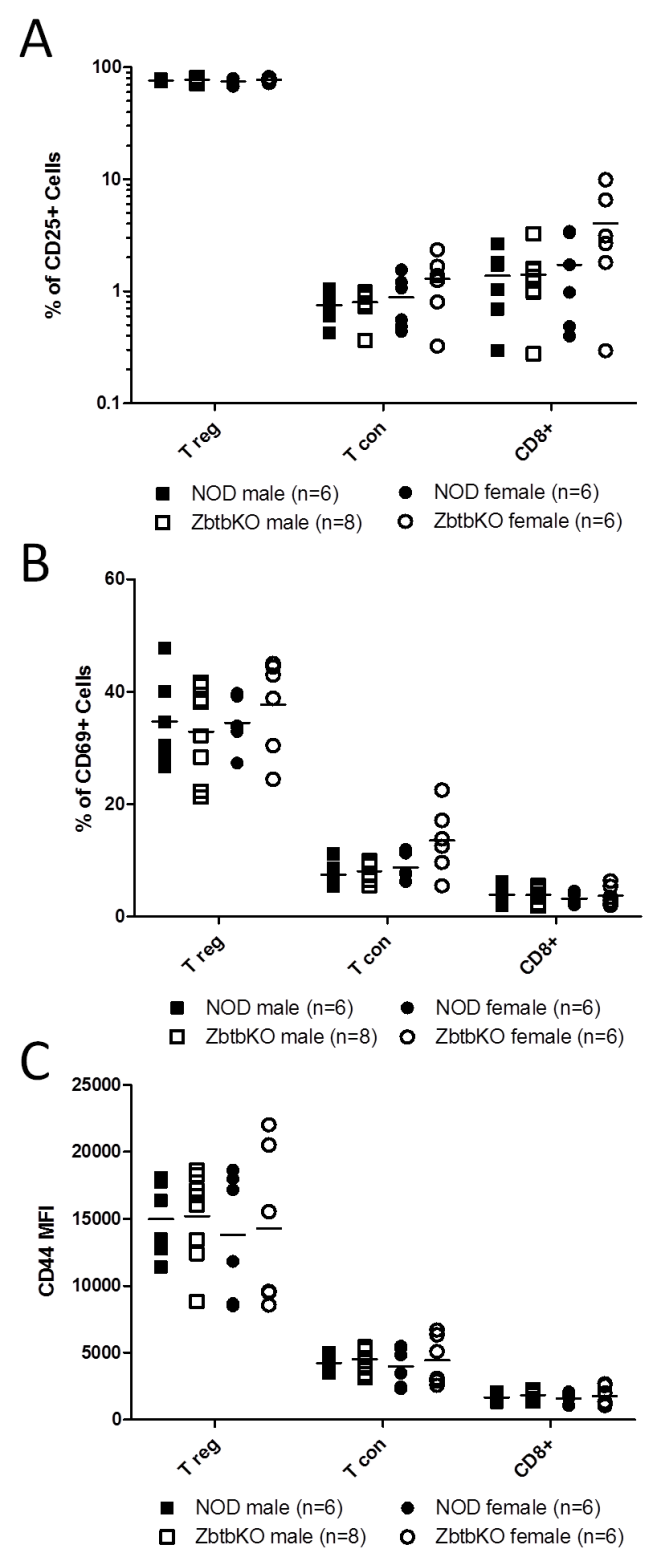
Activation markers on lymphocytes from pancreatic draining lymph node of Zbtb32 ^-/-^ mice. Lymphocytes from the pancreatic draining lymph node of male and female NOD and NOD.Zbtb32 ^-/-^ mice were stained for activation markers. Flow cytometry was used to determine cell surface expression of CD25 ( **A**) CD69 ( **B**) and CD44 ( **C**) on CD4 ^+^ Foxp3 ^+^ (Tregs), CD4 ^+^ Foxp3 ^-^ (Tcon) and CD8 ^+^ T cells. The data were combined from two independent repeats of this experiment.

Potential differences between wildtype and *Zbtb32 ^-/-^* lymphocyte phenotype of both male and female mice at 8 weeks of age were tested using flow cytometry. Activation markers on Foxp3 ^+^ regulatory T cells (Tregs), conventional CD4 ^+^ T cells (Tcons), and CD8 ^+^ T cells were measured without exogenous stimulation. As expected, higher activation was measured in the pancreatic-draining lymph nodes (pLNs) ( Figure 3) compared to the spleen ( Supplementary Figure 2), consistent with an autoimmune reaction to antigens acquired from pancreatic tissue. However, no statistically significant differences were observed between the NOD.Zbtb32 ^-/-^ mice and their control littermates for percent CD25 ^+^ ( Figure 3A), CD69 ^+^ ( Figure 3B), or CD44 MFI ( Figure 3C) (and Supplementary Figure 2A – C). T cells from the NOD.Zbtb32 ^-/-^ pLN, but not the spleen were more variable than the wildtype for these phenotypes, and in some comparisons trend higher (e.g. elevated CD25 expression in female CTLs seen in Figure 3A), suggesting a mild activation phenotype.

### *In vitro* stimulation of splenocytes is not significantly altered in NOD.Zbtb32 ^-/-^ mice

**Figure 4.**
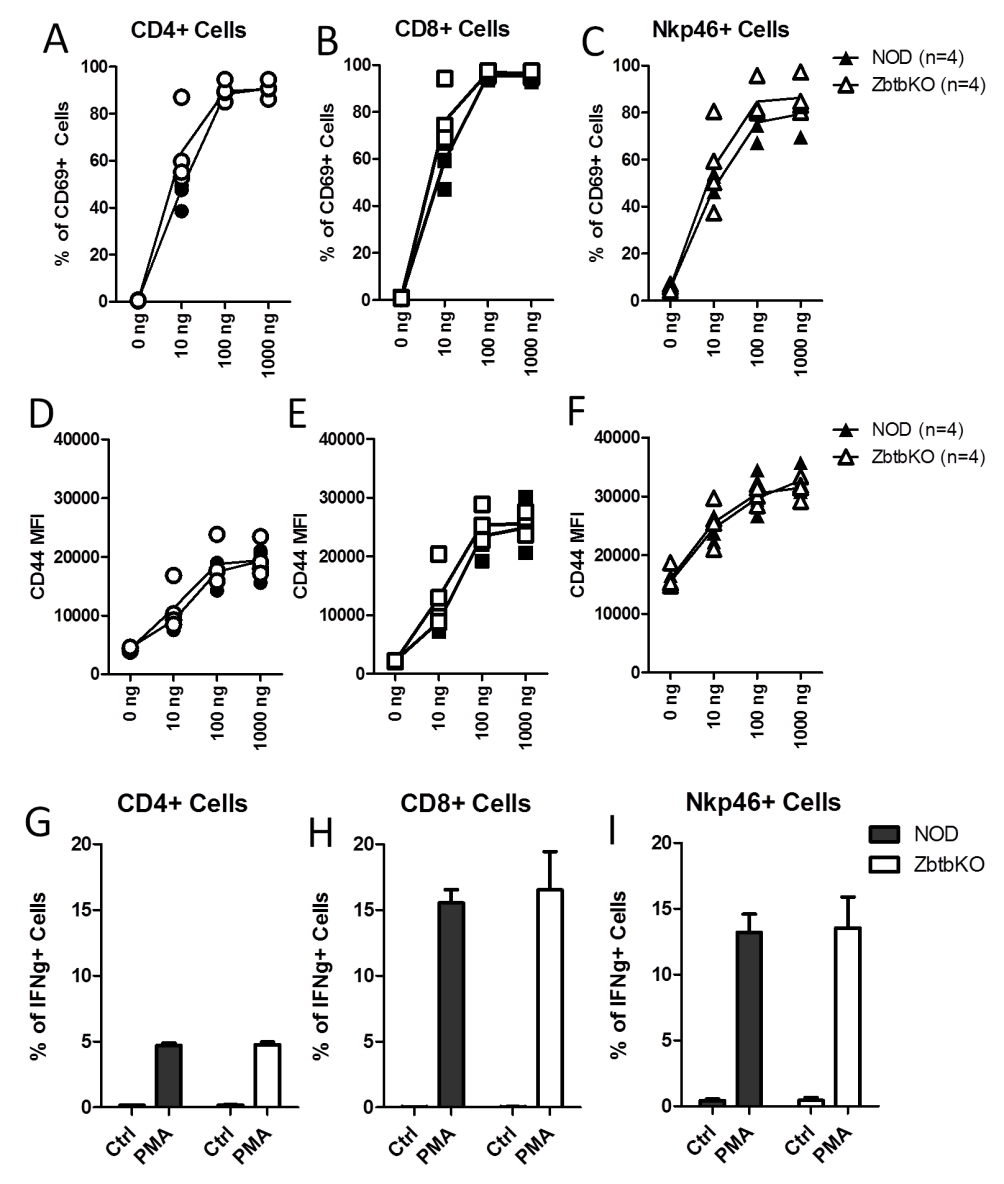
Activation markers and cytokine production in Zbtb32 ^-/-^ splenocytes after *ex vivo* stimulation. Freshly isolated splenocytes from female NOD.Zbtb32 ^-/-^ mice and control littermates were stimulated with the indicated dosages of anti-CD3 for 18 h. Surface expression of CD69 ( **A**– **C**) and CD44 ( **D**– **F**) was measured on CD4 ^+^, CD8 ^+^, and NKp46 ^+^ splenocytes. For IFNγ staining, splenocytes from female NOD.Zbtb32 ^-/-^ mice and their control littermates were stimulated with PMA and Ionomycin for 4 hours ( **G**– **I**). The percentage of cells positive for IFNγ in CD4 ^+^ ( **G**), CD8 ^+^ ( **H**), and NKp46 ^+^ cells ( **I**) is shown. The data were combined from two independent repeats of this experiment.

In previous reports describing Zbtb32 gene deletion in other genetic backgrounds, reported phenotypes included T-cell hypersensitivity, increased proliferation, and increased cytokine production ^10– 12, 14^. We therefore carried out parallel experiments using splenocytes from the NOD.Zbtb32 ^-/-^ mice. Splenocytes from both NOD.Zbtb32 ^-/-^ mice and NOD wildtype mice were stimulated with anti-CD3, and cell activation was assessed after 18 h by staining for surface CD69 ( Figure 4A–C), CD44 ( Figure 4D–F) and CD25 ( Supplementary Figure 3A–C). T cell activation markers increased with anti-CD3 stimulation, but no significant differences between wildtype and Zbtb32 ^-/-^ lymphocytes were observed. Levels of cell proliferation after 48 hours of anti-CD3 stimulation, as measured by dilution of Cell Trace Violet dye, were also the same between NOD wildtype and Zbtb32 ^-/-^( Supplementary Figure 3D and E). We next measured cytokine production by stimulating splenocytes with PMA and Ionomycin, and detecting IFNγ production in CD4 ^+^, CD8 ^+^, and Nkp46 ^+^ cells via intracellular cytokine staining. In all cases, the stimulation with PMA and Ionomycin produced the expected production of IFNγ, with higher expression in CD8 ^+^ and Nkp46 ^+^ cells. However, we did not find statistically significant differences between NOD.Zbtb32 ^-/-^ splenocytes and their matching controls ( Figure 4G–I). Therefore, unlike other Zbtb32 ^-/-^ strains, *ex vivo* T cell responses from NOD.Zbtb32 ^-/-^ mice were not significantly altered.

### No significant changes in peritoneal cavity B cells in NOD.Zbtb32 ^-/-^ mice

**Figure 5.**
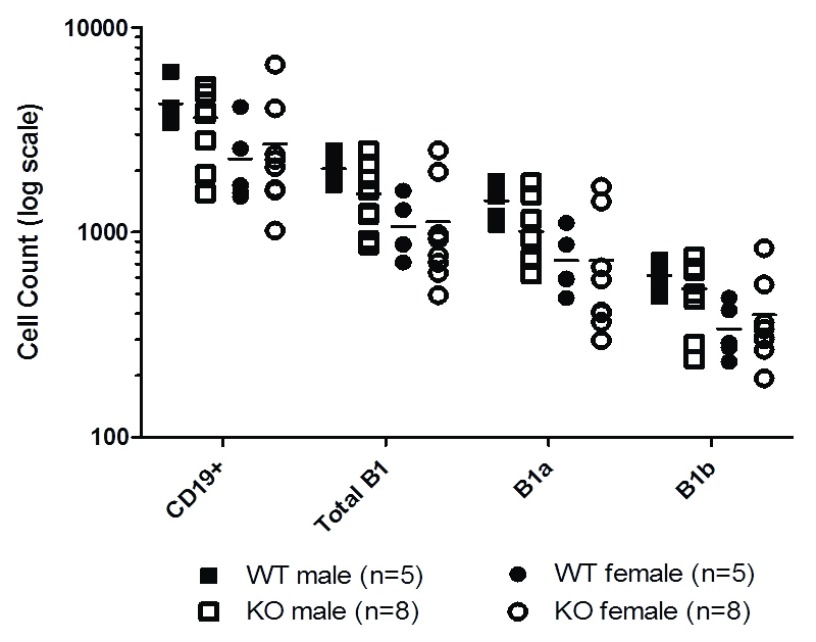
B cell populations within the peritoneal cavity. The number of B cells in the peritoneal cavity of 4 week-old mice were measured, including all CD19 ^+^ cells, all B1 cells, and the subpopulations of B1a and B1b cells. The data were combined from two independent experiments.

Because the T cell and diabetes phenotypes did not match the expected result based on overexpression of Zbtb32 in T cells ^6^, potential effects of Zbtb32 deletion on other cell types was considered. B1 cells, which primarily reside in the peritoneal cavity, display a constitutively high expression of Zbtb32 ^22, 23^. A related transcription factor, Zbtb20, was recently implicated as a regulator of terminal differentiation in germinal center B cells ^24^. The authors proposed that either Zbtb20 or Zbtb32 may play a critical role in either B1 cell proliferation or differentiation. B1 cells can be further divided into B1a and B1b cells based on their surface expression of CD5 ^25^. In NOD mice, B1 cells can be identified as CD19 ^+^B220 ^+^CD11b ^+^CD43 ^+^, with CD5 ^+^ and CD5 ^-^ subpopulations being labeled as B1a and B1b, respectively ( Figure 5). Since prior reports had indicated that Zbtb32 can regulate early B cell proliferation ^26^, B1 populations in the peritoneal cavity of NOD.Zbtb32 ^-/-^ mice and their wildtype controls were examined at 4 weeks of age ( Figure 5). NOD.Zbtb32 ^-/-^ mice trended toward fewer total B cells in their peritoneal cavity. But no statistically significant differences were observed in the proportion of B1 cells among all CD19 ^+^ cells between wildtype NOD and NOD.Zbtb32 ^-/-^ mice (analysis not shown). Therefore, the mild diabetes phenotype and the absence of the expected T cell activation phenotype are unlikely to be due to effects on B1 cells.

Raw data for blood glucose from Figure 2 The raw data values and the GraphPad Prism fole for blood glucose measurements. Click here for additional data file. Copyright: © 2018 Coley WD et al. 2018 Data associated with the article are available under the terms of the Creative Commons Zero "No rights reserved" data waiver (CC0 1.0 Public domain dedication).

Raw flow cytometry data from 8 week-old mice Raw flow cytometry sample data and the FlowJo analysis file for resting splenocytes and lymphocytes from 8 week old mice. Click here for additional data file. Copyright: © 2018 Coley WD et al. 2018 Data associated with the article are available under the terms of the Creative Commons Zero "No rights reserved" data waiver (CC0 1.0 Public domain dedication).

Raw flow cytometry data from *ex vivo* stimulated splenocytes Raw flow cytometry sample data and the FlowJo analysis file for *ex vivo* stimulated splenocytes, including cell proliferation and cytokine production. Click here for additional data file. Copyright: © 2018 Coley WD et al. 2018 Data associated with the article are available under the terms of the Creative Commons Zero "No rights reserved" data waiver (CC0 1.0 Public domain dedication).

Diabetes incidence in NOD.Zbtb32 ^-/-^ mice Click here for additional data file. Copyright: © 2018 Coley WD et al. 2018 Data associated with the article are available under the terms of the Creative Commons Zero "No rights reserved" data waiver (CC0 1.0 Public domain dedication).

Alignment Click here for additional data file. Copyright: © 2018 Coley WD et al. 2018 Data associated with the article are available under the terms of the Creative Commons Zero "No rights reserved" data waiver (CC0 1.0 Public domain dedication).

## Discussion

We previously focused on Zbtb32 as a potential regulator of T cell tolerance in the NOD mouse model, and found that this transcription regulator was preferentially induced in autoreactive CD4 ^+^ T cells after interacting with tolerogenic DCIR2 ^+^ DCs, and that overexpression in T cells inhibited both diabetes and effector T cell expansion ^6^. The available literature corroborated these findings but did not provide insight into how Zbtb32 might control T cell development and tolerance in a spontaneous autoimmune disease such as T1D ^9– 11, 27^. We initially hypothesized that the Zbtb32 ^-/-^ mutation in NOD mice would result in hyperactive T cells and increased diabetes. Instead, no significant changes in disease incidence in the NOD.Zbtb32 ^-/-^ mice were observed, and the increased T cell activation and differentiation observed in mutants from other genetic backgrounds were absent. No significant shifts in the number or percentage of the major lymphocyte populations were observed. Furthermore, we observed that the loss of Zbtb32 had no impact on intraperitoneal B1 cells, despite the fact that it is constitutively expressed in those cells in wildtype animals. In most of these readouts, a trend towards the expected phenotype was observed, suggesting a very mild phenotype, with only the long-term male disease incidence rates provided any evidence that the loss of Zbtb32 affected T1D disease pathogenesis. Although NOD females normally have higher incidence than males, this phenotype can vary greatly, and 50% incidence is within range of what we have observed for our colony (20–50% in males and 40–90% in females). 

Prior investigations of Zbtb32 knockout mice in other genetic backgrounds consistently showed an activated T cell phenotype that included hypersensitivity, increased cytokine production, and increased proliferation ^10– 14^. Furthermore, our results showed that overexpression of Zbtb32 delayed diabetes onset, limited T-cell proliferation, and decreased IFNγ production from autoreactive T cells in an adoptive transfer model of T1D ^6^. There are several possible reasons that may account for the observed lack of expected phenotype. One potential explanation for our unexpected results is that the CRISPR/Cas9 mediated deletion produced either an off-target effect or otherwise caused genetic instability at the deletion site. The use of CRISPR techniques in the NOD genetic background is a new approach that has, to our knowledge, only been described in two previous publications to date ^19, 28^. The CRIPSR/Cas9 mediated deletion was significantly smaller than other previously engineered Zbtb32 deletions (Compare Figure 1B to Supplementary Figure 4), but still disrupted the reading frame of the Zbtb32 gene. Our western blots ( Figure 1E) demonstrated that the protein had been eliminated, and the use of CAS9n minimized other potential mutations. Nevertheless, we re-examined our colony for any evidence of genetic instability or off-target effects by re-sequencing archived genomic DNA from founder mice and five subsequent generations of sibling crosses and found that the mutations remained stable ( Supplementary Figure 1) in every sequenced member of our NOD.Zbtb32 ^-/-^ colony. To identify potential off-target effects, we compared sequence homologies among all the transcription factors in the Zbtb family as well as sequence homologies for just the guide RNA strands used by the Cas9n enzyme. Both homology analyses indicated that only Zbtb16 was a potential target for unintended genetic editing. However, sequencing the Zbtb16 gene in multiple generations of the NOD.Zbtb32 ^-/-^ mouse colony revealed no off-target mutations of Zbtb16 (data not shown). These results therefore argue against the idea that our overall lack of phenotype can be explained by erroneous CRISPR/Cas9 activity.

 Genetic backgrounds can have a dramatic effect as genetic alterations combine to either mask or exacerbate disease phenotypes ^29^. Interestingly, the role of Zbtb32 in immune tolerance was examined in the BALB/c strain by using the Experimental Autoimmune Encephalitis (EAE) model. The loss of Zbtb32 in BALB/c mice had no significant impact on the EAE clinical score or timing of onset of paralysis suggest that the loss of Zbtb32, despite the observed activated T cell phenotype, was not sufficient to precipitate severe autoimmunity ^11^. A rapid and induced autoimmune model like EAE differs significantly from a spontaneous, chronic disease model like NOD, but the parallel results between our findings and the findings in Kang *et al.* ^11^ suggest that, in some contexts, other genes may be able to compensate for the loss of Zbtb32. Further support for this explanation comes from an examination of regulatory transcription factors in T _H_17-mediated autoimmune pathology ^30^. The authors found that although Zbtb32 gene expression was highly correlated with a protective phenotype, the loss of the Zbtb32 gene in the 129/Sv background did not produce the expected hypersensitive T _H_17 response and instead observed a diminished T _H_17 response after repeated stimulations. Based on their gene expression data, Gaublomme *et al*. hypothesized that while Zbtb32 normally suppresses pro-inflammatory responses, other unidentified transcription factors continued to upregulate pro-regulatory genes and therefore mask the expected phenotype in T _H_17 responses ^30^. The NOD.Zbtb32 ^-/-^ mice differed from past knockout strains in terms of the T cell response. Examinations of lymphocytes from the pLNs ( Figure 3) and the spleen ( Supplementary Figure 2) revealed only mild differences between wildtype and knockout littermates in NOD mice. Considering that the target pancreatic antigens are trafficked into the pLNs and by 8 weeks of age NOD mice display chronic autoimmunity and lymphocytic infiltration in the pancreatic islets ^31, 32^, we had expected to find increased levels of lymphocyte activation in the pLNs. The major phenotype observed in other Zbtb32 ^-/-^ strains was a hyperreactivity to *in vitro* T cell stimulation, manifest in higher proliferation and cytokine production ^10, 11, 14, 26^. However, T cells from NOD.Zbtb32 ^-/-^ mice showed no increase in proliferation or IFNγ production in response to *in vitro* stimulation. 

 It is possible that the lack of phenotype in the NOD genetic background, but not other backgrounds, was due to compensation from homologous proteins. The loss of Zbtb32 from birth could activate compensatory mechanisms in NOD mice that obscure any overt abnormalities in adult mice. It is even possible that a compensatory mechanism was selected for in the course of breeding NOD.Zbtb32 ^-/-^ mice. Our incidence data includes one mutant mouse from our first generation of the colony that developed hyperglycemia at an abnormally early age (8 weeks), and three out of the four mutated alleles proved to be poor breeders. Zbtb32 is constitutively expressed in the testis ^33^, but its role in fertility is not clear ^34^. These unexpected results could potentially be explained by compensatory mechanisms that were either already present within the NOD genetic background, or were selected for during the generation of this NOD.Zbtb32 ^-/-^ mouse colony. Therefore, an alternative approach targeting expression in adults could yield a different result.

Taken together, our results show that the systemic loss of Zbtb32 in NOD mice does not lead to a hypersensitive T cell phenotype and increased diabetes pathogenesis. Although the NOD.Zbtb32 ^-/-^ male mice displayed some increase in diabetes incidence compared to littermate controls, these trends did not reach statistical significance. Therefore, we conclude that NOD mice with the loss of Zbtb32 have a mild phenotype that may be a result of compensation by a currently unknown mechanism. In a broader context, these data do not support a major role for Zbtb32 as a target for treatment of autoimmune diabetes.

## Data availability

The data referenced by this article are under copyright with the following copyright statement: Copyright: © 2018 Coley WD et al.

Data associated with the article are available under the terms of the Creative Commons Zero "No rights reserved" data waiver (CC0 1.0 Public domain dedication). 

**Dataset 1: Raw images for Figure 1** The raw images for the genotyping gels and western blots seen in Figure 1. 10.5256/f1000research.13864.d197441 ^35^

**Dataset 2: Raw data for blood glucose from Figure 2** The raw data values and the GraphPad Prism fole for blood glucose measurements. 10.5256/f1000research.13864.d197442 ^36^

**Dataset 3: Raw flow cytometry data from 8 week-old mice:** Raw flow cytometry sample data and the FlowJo analysis file for resting splenocytes and lymphocytes from 8 week old mice. 10.5256/f1000research.13864.d197443 ^37^

**Dataset 4: Raw flow cytometry data from *ex vivo* stimulated splenocytes:** Raw flow cytometry sample data and the FlowJo analysis file for *ex vivo* stimulated splenocytes, including cell proliferation and cytokine production 10.5256/f1000research.13864.d197444 ^38^

**Dataset 5:** Diabetes incidence in NOD.Zbtb32 ^-/-^ mice 10.5256/f1000research.13864.d224340 ^39^

**Dataset 6:** Alignment 10.5256/f1000research.13864.d197446 ^40^

## Supplementary material

**Supplementary Table 1. Alignment of NOD.Zbtb32 mutant alleles.** The four mutant alleles of Zbtb32 that were produced by CRISPR/Cas9 were then sequenced and aligned. Allele B was chosen to serve as the only mutation in the colony.

**Supplemental Figure 1. The CRISPR/Cas9 deletion remained stable in the NOD.Zbtb32 colony.** We archived breeder genealogies and genomic DNA from all mice in the NOD.Zbtb32 mouse colony. A genealogy of breeding cages carrying the B allele of the mutated Zbtb32 gene is depicted in ( **A**). A total of 16 archived DNA samples were sequenced and then aligned in order to verify that the mutation remained stable in the colony ( **B**). These archived samples included 1 founder of the B line, 13 homozygous knockout descendants, and 2 homozygous wildtype offspring.

**Supplemental Figure 2. Activation markers from resting splenocytes in NOD.Zbtb32 ^-/-^ mice.** We examined populations of Treg, Tcon, and CD8 ^+^ T cells in freshly dissected spleens from both male and female mice and their littermate controls. Flow cytometry was used to determine the ratio of CD25 ^+^ ( **A**) CD69 ^+^ ( **B**) and cell surface expression of CD44 ( **C**) on the indicated splenocytes.

**Supplemental Figure 3. Activation markers on** ***ex vivo*** **-stimulated lymphocytes from pancreatic draining lymph nodes.** After isolating lymphocytes from the pancreatic draining lymph nodes of NOD.Zbtb32 ^-/-^ mice and stimulating them with anti-CD3 as indicated, we stained for activation markers in both male and female mice as well as their matching controls. Flow cytometry was used to determine the ratio expressing CD25 ( **A**– **C**) for CD4 ^+^, CD8 ^+^, and Nkp46 ^+^ splenocytes. Additionally, we measured cell proliferation in splenocytes after 48 h in response to 1000 ng of anti-CD3 ( **D**& **E**).

**Supplemental Figure 4. Other zbtb32 KO maps.** For comparison purposes, the mutant mRNA sequences that arise from each of the previous 3 mutant strains are depicted here. The mature mRNAs contain no introns, and non-coding portions are shown as thinner than the coding portions. Each of the previous three mutants was produced by deleting large portions of the Zbtb32 gene and replacing the sequence with selection and/or reporter genes.
