# Supplementary material for: Loss of Zbtb32 in NOD mice does not significantly alter T cell responses
Source: F1000Res. 2018 Nov 5;7:318. Originally published 2018 Mar 14. [Version 2] doi: 10.12688/f1000research.13864.2 (PMC5909056; doi:10.12688/f1000research.13864.2)
Supplement: Raw images for Figure 1 — The raw images for the genotyping gels and western blots seen in Figure 1. [file f1000research-7-18503-s0000.tgz › 245ff0d7-7947-4bda-a66f-e2f36566a852_Dataset_1_F1_Raw_Gel_Images.pptx]

## Slide 1
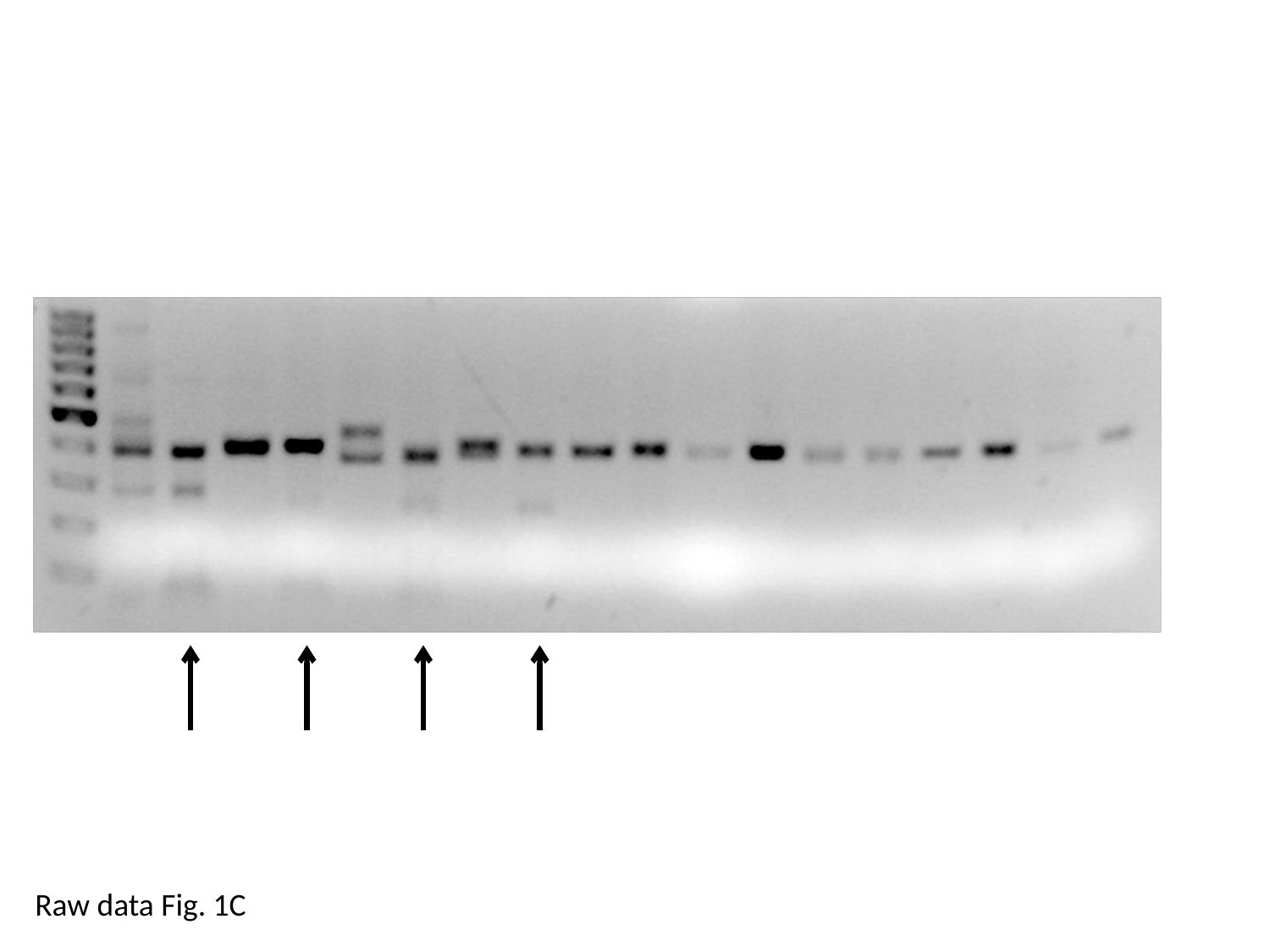

Raw data Fig. 1C

## Slide 2
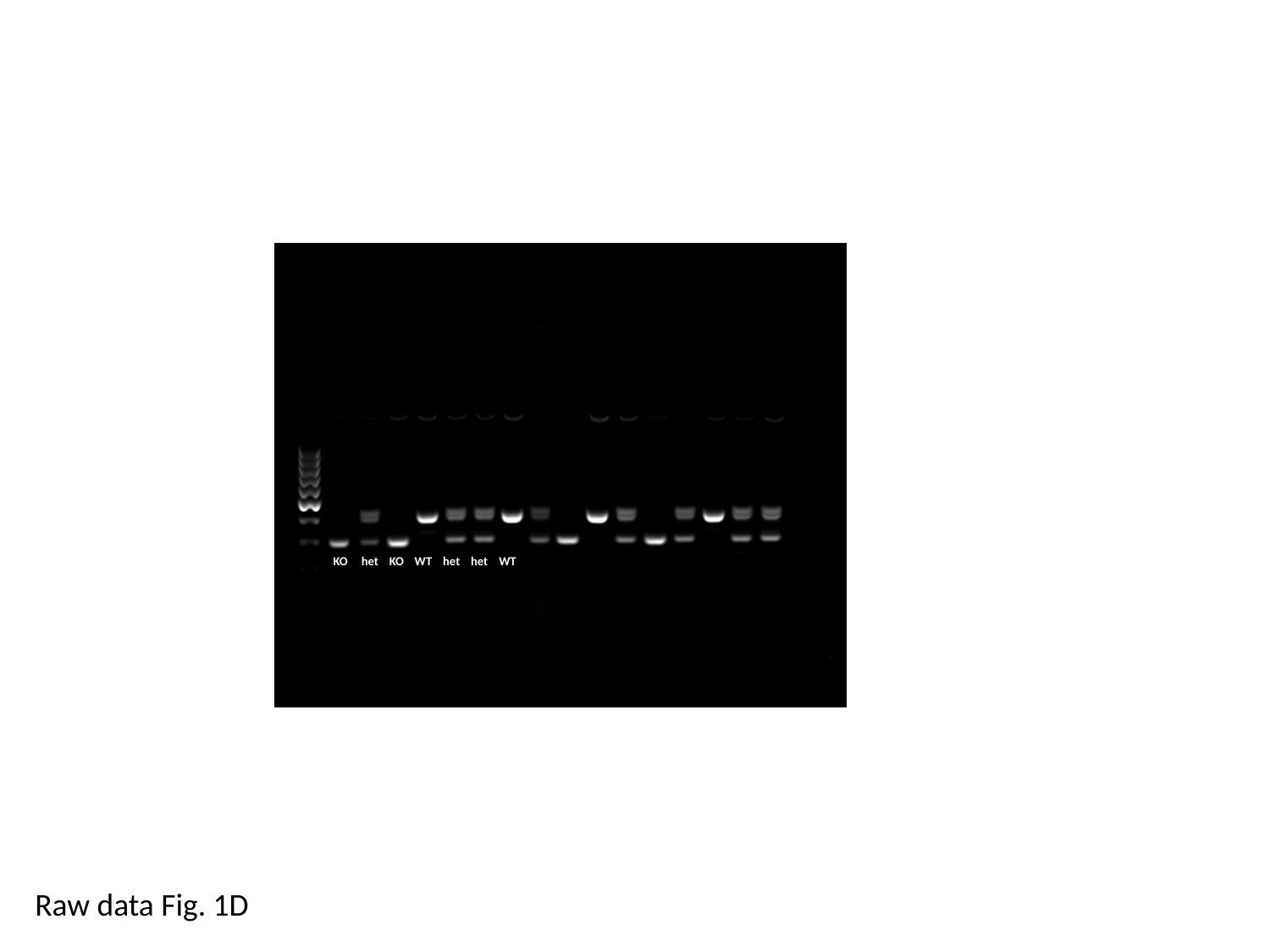

KO het KO WT het het WT
Raw data Fig. 1D

## Slide 3
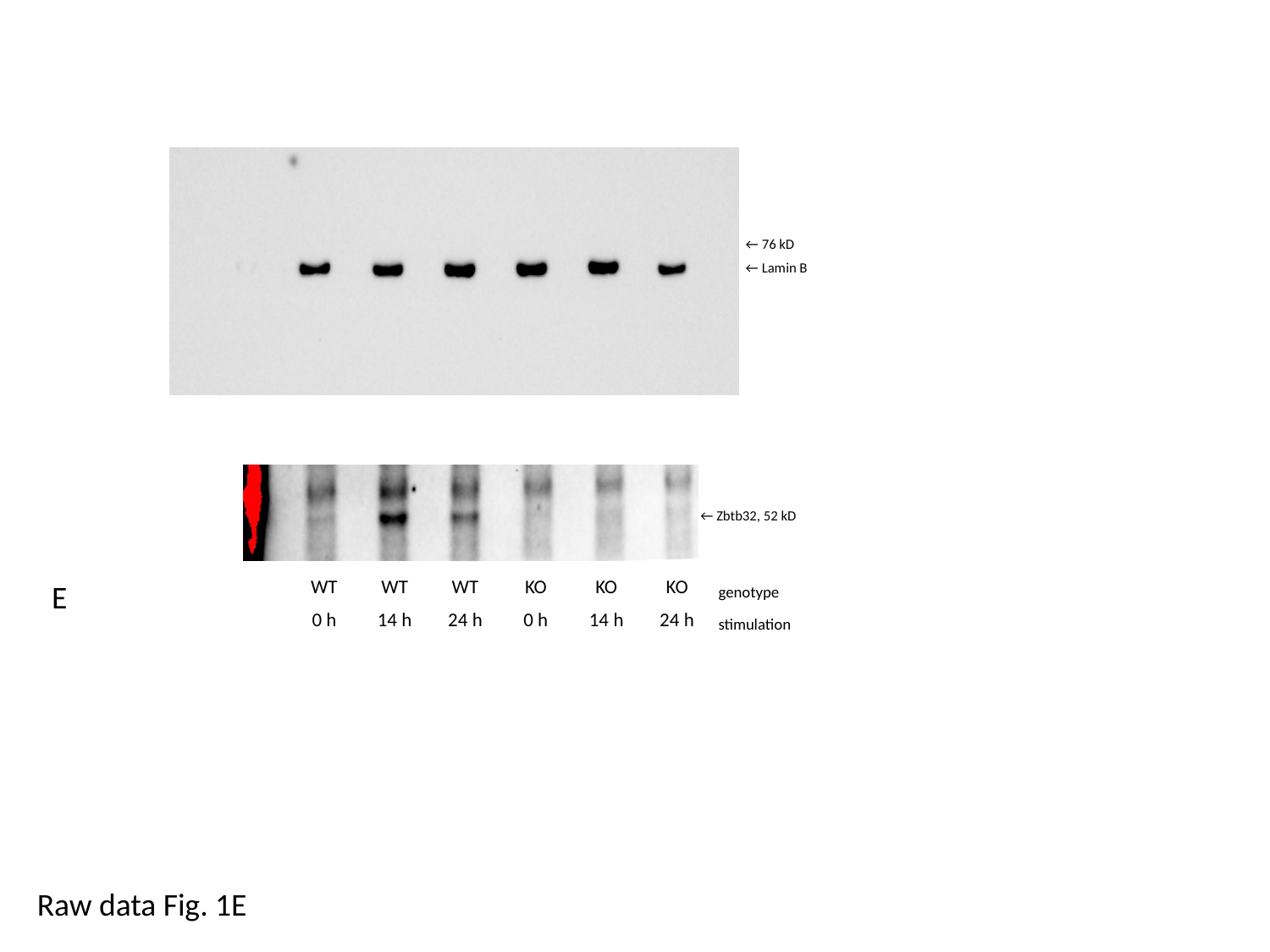

← 76 kD
← Lamin B
← Zbtb32, 52 kD
E
| WT | WT | WT | KO | KO | KO |
| --- | --- | --- | --- | --- | --- |
| 0 h | 14 h | 24 h | 0 h | 14 h | 24 h |
| | | | | | |
genotype
stimulation
Raw data Fig. 1E
