# Supplementary material for: Loss of Zbtb32 in NOD mice does not significantly alter T cell responses
Source: F1000Res. 2018 Nov 5;7:318. Originally published 2018 Mar 14. [Version 2] doi: 10.12688/f1000research.13864.2 (PMC5909056; doi:10.12688/f1000research.13864.2)
Supplement: Alignment [file f1000research-7-18503-s0005.tgz › 1be6f759-047d-45af-a2ea-c99f886af0dc_Dataset_6_Alignment.docx]

guideRNA-2 ------------------------------------------------------------

Zbtb16_Gene aggtaagtcgcatcctgtggtatttgaggttaggctttcagtatactaattttaaggata

4643 -------------------------------------AAAAGGACGTCTAGTTTAGAATA

5064 ----------------------------------------------------TTAGAATA

7605 ----------------------------------------------------TAGGATTA

6288 ----------------------------------------------------------TA

5224 ----------------------------------------------------------TA

6411 ------------------------------------GAACTTACCGTTATTTTAGGAATA

6000 ---------------------------------------------GGCATACTTAGAATA

6004 ----------------------------------------ACGTACGTCTACTTAGAATA

5999 ----------------------------------------CGGCACTAACTTTACGAATA

4633 --------------------------------------AAGAAAAACCAGAATCGGCTTA

guideRNA-2 ------------------------------------------------------------

Zbtb16_Gene tacagcttagttcacag-cagttctatagtttaaaat--ggctGCTAGGATCCTCCCTTG

4643 --CAGCTTAGTTCACAG-CAGTTCTATAGTTTAAAT---GGCTGCTAGGATCCTCCCTTG

5064 --CAGCTTAGTTCACAG-CAGTTCTATAGTTTAAAAT--GGCTGCTAGGATCCTCCCTTG

7605 --CAGCTTAGTTCACAG-CAGTTCTATAGTTTAAAAT--GGCTGCTAGGATCCTCCCTTG

6288 --CAGCTTAGTTCACAG-CAGTTCTATAGTTTAAAAT--GGCTGCTAGGATCCTCCCTTG

5224 --CAGCTTAGTTCACAG-CAGTTCTATAGTTTAAAAT--GGCTGCTAGGATCCTCCCTTG

6411 --CAGCTTAGTTCACAG-CAGTTCTATAGTTTAAAAT--GGCTGCTAGGATCCTCCCTTG

6000 --CAGCTTAGTTCACAG-CAGTTCTATAGTTTAAAAT-GGGCTGCTAGGATCCTCCCTTG

6004 --CAGCTTAGTTCACAG-CAGTTCTATAGTTTAAAATAGGGCTGCTAGGATCCTCCCTAG

5999 --CAGCTTAGTTCACAG-CAGTTCTATAGTTTAAAATGGGCTGGCTAGGATCCTCCCTTG

4633 ATCAGCTTAGTTCACAGTCAGTTCTATAGTTTAAAAT--GGCTGCTAGGATCCTCCCTTG

guideRNA-2 ------------------------------------------------------------

Zbtb16_Gene CCTTTTGTGGATTGGAGGGGGTTTACAGTGTCCCCTAAAAGGAGTCTACCACAAATAGAA

4643 CCTTTTGTGGATTGGAGGGGGTTTACAGTGTCCCCTAAAAGGAGTCTACCACAAATAGAA

5064 CCTTTTGTGGATTGGAGGGGGTTTACAGTGTCCCCTAAAAGGAGTCTACCACAAATAGAA

7605 CCTTTTGTGGATTGGAGGGGGTTTACAGTGTCCCCTAAAAGGAGTCTACCACAAATAGAA

6288 CCTTTTGTGGATTGGAGGGGGTTTACAGTGTCCCCTAAAAGGAGTCTACCACAAATAGAA

5224 CCTTTTGTGGATTGGAGGGGGTTTACAGTGTCCCCTAAAAGGAGTCTACCACAAATAGAA

6411 CCTTTTGTGGATTGGAGGGGGTTTACAGTGTCCCCTAAAAGGAGTCTACCACAAATAGAA

6000 CCTTTTGTGGATTGGAGGGGGTTTACAGTGTCCCCTAAAAGGAGTCTACCACAAATAGAA

6004 CCTTTTGTGGATTGGAGGGGGTTTACAGTGTCCCCTAAAAGGAGTCTACCACAAATAGAA

5999 CCTTTTGTGGATTGGAGGGGGTTTACAGTGTCCCCTAAAAGGAGTCTACCACAAATAGAA

4633 CCTTTTGTGGATTGGAGGGGGTTTACAGTGTCCCCTAAAAGGAGTCTACCACAAATAGAA

guideRNA-2 --------------------CAATCATGGATCCCCCATTG--------------------

Zbtb16_Gene AGGATGAATGTGCTACAAGAAAATCCTGGATGCCAGATTGGAGTCTCAACTATCTTCTTT

4643 AGGATGAATGTGCTACAAGAAAATCCTGGATGCCAGATTGGAGTCTCAACTATCTTCTTT

5064 – line C AGGATGAATGTGCTACAAGAAAATCCTGGATGCCAGATTGGAGTCTCAACTATCTTCTTT

7605 AGGATGAATGTGCTACAAGAAAATCCTGGATGCCAGATTGGAGTCTCAACTATCTTCTTT

6288 AGGATGAATGTGCTACAAGAAAATCCTGGATGCCAGATTGGAGTCTCAACTATCTTCTTT

5224 AGGATGAATGTGCTACAAGAAAATCCTGGATGCCAGATTGGAGTCTCAACTATCTTCTTT

6411 AGGATGAATGTGCTACAAGAAAATCCTGGATGCCAGATTGGAGTCTCAACTATCTTCTTT

6000 AGGATGAATGTGCTACAAGAAAATCCTGGATGCCAGATTGGAGTCTCAACTATCTTCTTT

6004 AGGATGAATGTGCTACAAGAAAATCCTGGATGCCAGATTGGAGTCTCAACTATCTTCTTT

5999 - WT AGGATGAATGTGCTACAAGAAAATCCTGGATGCCAGATTGGAGTCTCAACTATCTTCTTT

4633 AGGATGAATGTGCTACAAGAAAATCCTGGATGCCAGATTGGAGTCTCAACTATCTTCTTT

**** ***** ** ****

guideRNA-2 ------------------------------------------------------------

Zbtb16_Gene CTCCCCTCCTTCCT-CCCCCCACCTCTCTTGGATAGTCTGTAGCCCTCACTAGCCTGATA

4643 CTCCCCTCCTTCCTCCCCCCCACCTCTCTTGGATAGTCTGTAGCCCTCACTAGCCTGATA

5064 CTCCCCTCCTTCCTCCCCCCCACCTCTCTTGGATAGTCTGTAGCCCTCACTAGCCTGATA

7605 CTCCCCTCCTTCCTCCCCCCCACCTCTCTTGGATAGTCTGTAGCCCTCACTAGCCTGATA

6288 CTCCCCTCCTTCCTCCCCCCCACCTCTCTTGGATAGTCTGTAGCCCTCACTAGCCTGATA

5224 CTCCCCTCCTTCCTCCCCCCCACCTCTCTTGGATAGTCTGTAGCCCTCACTAGCCTGATA

6411 CTCCCCTCCTTCCTCCCCCCCACCTCTCTTGGATAGTCTGTAGCCCTCACTAGCCTGATA

6000 CTCCCCTCCTTCCTCCCCCCCACCTCTCTTGGATAGTCTGTAGCCCTCACTAGCCTGATA

6004 CTCCCCTCCTTCCTCCCCCCCACCTCTCTTGGATAGTCTGTAGCCCTCACTAGCCTGATA

5999 CTCCCCTCCTTCCTCCCCCCCACCTCTCTTGGATAGTCTGTAGCCCTCACTAGCCTGATA

4633 CTCCCCTCCTTCCTCCCCCCCACCTCTCTTGGATAGTCTGTAGCCCTCACTAGCCTGATA

guideRNA-2 ------------------------------------------------------------

Zbtb16_Gene CTCACTGTGTAGTGTAGCTAGGTTTGCACTTGCAACAAACCTCCTGCCTCAGCCCCCTAA

4643 CTCACTGTGTAGTGTAGCTAGGTTTGCACTTGCAACAAACCTCCTGCCTCAGCCCCCTAA

5064 CTCACTGTGTAGTGTAGCTAGGTTTGCACTTGCAACAAACCTCCTGCCTCAGCCCCCTAA

7605 CTCACTGTGTAGTGTAGCTAGGTTTGCACTTGCAACAAACCTCCTGCCTCAGCCCCCTAA

6288 CTCACTGTGTAGTGTAGCTAGGTTTGCACTTGCAACAAACCTCCTGCCTCAGCCCCCTAA

5224 CTCACTGTGTAGTGTAGCTAGGTTTGCACTTGCAACAAACCTCCTGCCTCAGCCCCCTAA

6411 CTCACTGTGTAGTGTAGCTAGGTTTGCACTTGCAACAAACCTCCTGCCTCAGCCCCCTAA

6000 CTCACTGTGTAGTGTAGCTAGGTTTGCACTTGCAACAAACCTCCTGCCTCAGCCCCCTAA

6004 CTCACTGTGTAGTGTAGCTAGGTTTGCACTTGCAACAAACCTCCTGCCTCAGCCCCCTAA

5999 CTCACTGTGTAGTGTAGCTAGGTTTGCACTTGCAACAAACCTCCTGCCTCAGCCCCCTAA

4633 CTCACTGTGTAGTGTAGCTAGGTTTGCACTTGCAACAAACCTCCTGCCTCAGCCCCCTAA

guideRNA-2 ------------------------------------------------------------

Zbtb16_Gene GTGCTAGGATTATAGTTATGGGTCCCTGTGCct---------------------------

4643 GTGCTAGGATTATAGTTATGGGTCCCTGTGCCA--------------GTGGAAAATGGGG

5064 GTGCTAGGATTATAGTTATGGGTCCCTGTGCAA---------------------------

7605 GTGCTAGGATTATAGTTATGGGTCCCTGTGCAA---------------------------

6288 GTGCTAGGATTATAGTTATGGGTCCCTGTGCAA---------------------------

5224 GTGCTAGGATTATAGTTATGGGTCCCTGTGCAA---------------------------

6411 GTGCTAGGATTATAGTTATGGGTCCCTGTGCCAAG-----------TTGGAAAGGGGGGG

6000 GTGCTAGGATTATAGTTATGGGTCCCTGTGCAA-----------------------ATGT

6004 GTGCTAGGATTATAGTTATGGGTCCCTGTGCTG----------------ATGGAGTGTGT

5999 GTGCTAGGATTATAGTTATGGTCCCCTGTGCTGA-------------AGGAAATGGGGGG

>guideRNA-2

CAATCATGGATCCCCCATTG

>consensus

aggtaagtcgcatcctgtggtatttgaggttaggctttcagtatactaattttaaggatatacagcttagttcacagcagttctatagtttaaaatggctGCTAGGATCCTCCCTTGCCTTTTGTGGATTGGAGGGGGTTTACAGTGTCCCCTAAAAGGAGTCTACCACAAATAGAAAGGATGAATGTGCTACAAGAAAATCCTGGATGCCAGATTGGAGTCTCAACTATCTTCTTTCTCCCCTCCTTCCTCCCCCCACCTCTCTTGGATAGTCTGTAGCCCTCACTAGCCTGATACTCACTGTGTAGTGTAGCTAGGTTTGCACTTGCAACAAACCTCCTGCCTCAGCCCCCTAAGTGCTAGGATTATAGTTATGGGTCCCTGTGCctggcttcaacctcctcaattgcactgcatttgtagctaatagctatgttgtgtgtgcacacatatacatatatatgtacatatacatacatcaaacatg

>4633

AAGAAAAACCAGAATCGGCTTAATCAGCTTAGTTCACAGTCAGTTCTATAGTTTAAAATGGCTGCTAGGATCCTCCCTTGCCTTTTGTGGATTGGAGGGGGTTTACAGTGTCCCCTAAAAGGAGTCTACCACAAATAGAAAGGATGAATGTGCTACAAGAAAATCCTGGATGCCAGATTGGAGTCTCAACTATCTTCTTTCTCCCCTCCTTCCTCCCCCCCACCTCTCTTGGATAGTCTGTAGCCCTCACTAGCCTGATACTCACTGTGTAGTGTAGCTAGGTTTGCACTTGCAACAAACCTCCTGCCTCAGCCCCCTAAGTGCTAGGATTATAGTTATGGGTCCCTGTGCCCAACAGCCGGCCACCTAAGAAGAGGAAAATAAAAATGGAGTATTAGAAGATGCCAAAACTCGTCGGCCTTGGAAAAAAAAATAATTTTCTTTTCCT

>4643

AAAAGGACGTCTAGTTTAGAATACAGCTTAGTTCACAGCAGTTCTATAGTTTAAATGGCTGCTAGGATCCTCCCTTGCCTTTTGTGGATTGGAGGGGGTTTACAGTGTCCCCTAAAAGGAGTCTACCACAAATAGAAAGGATGAATGTGCTACAAGAAAATCCTGGATGCCAGATTGGAGTCTCAACTATCTTCTTTCTCCCCTCCTTCCTCCCCCCCACCTCTCTTGGATAGTCTGTAGCCCTCACTAGCCTGATACTCACTGTGTAGTGTAGCTAGGTTTGCACTTGCAACAAACCTCCTGCCTCAGCCCCCTAAGTGCTAGGATTATAGTTATGGGTCCCTGTGCCAGTGGAAAATGGGGGAGAGATCTCGAGCTTTTGCTCTTAGAGACCCTTCTCCTCTCGTGCTTGTTTAGATCTCTGCTCTGCGCTCTGGCCCCCCCG

>5064

TTAGAATACAGCTTAGTTCACAGCAGTTCTATAGTTTAAAATGGCTGCTAGGATCCTCCCTTGCCTTTTGTGGATTGGAGGGGGTTTACAGTGTCCCCTAAAAGGAGTCTACCACAAATAGAAAGGATGAATGTGCTACAAGAAAATCCTGGATGCCAGATTGGAGTCTCAACTATCTTCTTTCTCCCCTCCTTCCTCCCCCCCACCTCTCTTGGATAGTCTGTAGCCCTCACTAGCCTGATACTCACTGTGTAGTGTAGCTAGGTTTGCACTTGCAACAAACCTCCTGCCTCAGCCCCCTAAGTGCTAGGATTATAGTTATGGGTCCCTGTGCAA

>5999

CGGCACTAACTTTACGAATACAGCTTAGTTCACAGCAGTTCTATAGTTTAAAATGGGCTGGCTAGGATCCTCCCTTGCCTTTTGTGGATTGGAGGGGGTTTACAGTGTCCCCTAAAAGGAGTCTACCACAAATAGAAAGGATGAATGTGCTACAAGAAAATCCTGGATGCCAGATTGGAGTCTCAACTATCTTCTTTCTCCCCTCCTTCCTCCCCCCCACCTCTCTTGGATAGTCTGTAGCCCTCACTAGCCTGATACTCACTGTGTAGTGTAGCTAGGTTTGCACTTGCAACAAACCTCCTGCCTCAGCCCCCTAAGTGCTAGGATTATAGTTATGGTCCCCTGTGCTGAAGGAAATGGGGGGGAGAGATCTCGACCTCGTGCTCTTAGAGACCCTTCTCCTCTCGTGTGTGTTGAGACGCGTGCTGTGCGCTCTGGGCGCCCCGTGTGAGTCTGGGGTGGCCCTTTCGCCTCTGTCGCTGCGCTTTATAAGTCTCTCTACACATTTAATTTTTGATGATGATGCTGCTGAC

>6004

ACGTACGTCTACTTAGAATACAGCTTAGTTCACAGCAGTTCTATAGTTTAAAATAGGGCTGCTAGGATCCTCCCTAGCCTTTTGTGGATTGGAGGGGGTTTACAGTGTCCCCTAAAAGGAGTCTACCACAAATAGAAAGGATGAATGTGCTACAAGAAAATCCTGGATGCCAGATTGGAGTCTCAACTATCTTCTTTCTCCCCTCCTTCCTCCCCCCCACCTCTCTTGGATAGTCTGTAGCCCTCACTAGCCTGATACTCACTGTGTAGTGTAGCTAGGTTTGCACTTGCAACAAACCTCCTGCCTCAGCCCCCTAAGTGCTAGGATTATAGTTATGGGTCCCTGTGCTGATGGAGTGTGTGTGAGAGATCGAGGCCTTGCGCTTAAGGAGCCCCTTCGCCTCGTGCTTGAGTTGAGGCCTGGCCTGGGCGCTGGGGCCGCCGCGT

>6411

GAACTTACCGTTATTTTAGGAATACAGCTTAGTTCACAGCAGTTCTATAGTTTAAAATGGCTGCTAGGATCCTCCCTTGCCTTTTGTGGATTGGAGGGGGTTTACAGTGTCCCCTAAAAGGAGTCTACCACAAATAGAAAGGATGAATGTGCTACAAGAAAATCCTGGATGCCAGATTGGAGTCTCAACTATCTTCTTTCTCCCCTCCTTCCTCCCCCCCACCTCTCTTGGATAGTCTGTAGCCCTCACTAGCCTGATACTCACTGTGTAGTGTAGCTAGGTTTGCACTTGCAACAAACCTCCTGCCTCAGCCCCCTAAGTGCTAGGATTATAGTTATGGGTCCCTGTGCCAAGTTGGAAAGGGGGGGGAGAGAGCTCGAGGCCTTGCTCTAAGGGACCCCCTTCCCCTTGTGTTTGATTTGACGCCTGCTGTGCGCTCTGGGG

>6288

TACAGCTTAGTTCACAGCAGTTCTATAGTTTAAAATGGCTGCTAGGATCCTCCCTTGCCTTTTGTGGATTGGAGGGGGTTTACAGTGTCCCCTAAAAGGAGTCTACCACAAATAGAAAGGATGAATGTGCTACAAGAAAATCCTGGATGCCAGATTGGAGTCTCAACTATCTTCTTTCTCCCCTCCTTCCTCCCCCCCACCTCTCTTGGATAGTCTGTAGCCCTCACTAGCCTGATACTCACTGTGTAGTGTAGCTAGGTTTGCACTTGCAACAAACCTCCTGCCTCAGCCCCCTAAGTGCTAGGATTATAGTTATGGGTCCCTGTGCAA

>7605

TAGGATTACAGCTTAGTTCACAGCAGTTCTATAGTTTAAAATGGCTGCTAGGATCCTCCCTTGCCTTTTGTGGATTGGAGGGGGTTTACAGTGTCCCCTAAAAGGAGTCTACCACAAATAGAAAGGATGAATGTGCTACAAGAAAATCCTGGATGCCAGATTGGAGTCTCAACTATCTTCTTTCTCCCCTCCTTCCTCCCCCCCACCTCTCTTGGATAGTCTGTAGCCCTCACTAGCCTGATACTCACTGTGTAGTGTAGCTAGGTTTGCACTTGCAACAAACCTCCTGCCTCAGCCCCCTAAGTGCTAGGATTATAGTTATGGGTCCCTGTGCAA

>6000

GGCATACTTAGAATACAGCTTAGTTCACAGCAGTTCTATAGTTTAAAATGGGCTGCTAGGATCCTCCCTTGCCTTTTGTGGATTGGAGGGGGTTTACAGTGTCCCCTAAAAGGAGTCTACCACAAATAGAAAGGATGAATGTGCTACAAGAAAATCCTGGATGCCAGATTGGAGTCTCAACTATCTTCTTTCTCCCCTCCTTCCTCCCCCCCACCTCTCTTGGATAGTCTGTAGCCCTCACTAGCCTGATACTCACTGTGTAGTGTAGCTAGGTTTGCACTTGCAACAAACCTCCTGCCTCAGCCCCCTAAGTGCTAGGATTATAGTTATGGGTCCCTGTGCAAATGTGTGAGAGTTTAGACCTTTGCTTTGAGACCCTCTTCCTCTCGCGCTTGATTAGACCTCTGCTGTGCGCTGTGGCCCCCCCGTGCGAAACTCTTGTGGCACCCTCGCCCGTGTCTCTGTGCTTTTAAAATCTATCCATTTAAAATATTTTAGAGCCCTGCGCGCTCTTTTCTGTGGCATATATCGTGTCTG

>5224

TACAGCTTAGTTCACAGCAGTTCTATAGTTTAAAATGGCTGCTAGGATCCTCCCTTGCCTTTTGTGGATTGGAGGGGGTTTACAGTGTCCCCTAAAAGGAGTCTACCACAAATAGAAAGGATGAATGTGCTACAAGAAAATCCTGGATGCCAGATTGGAGTCTCAACTATCTTCTTTCTCCCCTCCTTCCTCCCCCCCACCTCTCTTGGATAGTCTGTAGCCCTCACTAGCCTGATACTCACTGTGTAGTGTAGCTAGGTTTGCACTTGCAACAAACCTCCTGCCTCAGCCCCCTAAGTGCTAGGATTATAGTTATGGGTCCCTGTGCAA

>zbtb16_fullGene

GGCCACTCGGCCGCTGGGCTCGTGCGGGGTTCTCTGGTTGCTGCTGCAGC

AGCGATCGCCAGTGCCCGTTGGGGGTCAGCTAGAAAGCGGCGCTGAGGCT

GTCAGCTTGGGCACAGCTGCCTCACCAACCTTTCTTCTCCGGGCACGGTC

CGGTCCGGgtgagaggtggcaggccgggaggtctgccagggctgcgggcg

cgtgtagtcggggactgggtgtctttgtacaggcaagggctggcggagga

gcagagagcctgtgggtgctcatatgtatgcggaccggtgcgcttggcgt

gagataggattgtgtcttatttatttgtgtttattcgctggcgagctggg

gagctagacccggaggaagcaaggagtgaacctgggggtcgggtgggagg

ggactggtatccgatcgggttttgttttctttagagaaaacgtggtggga

tttttttcttctttttttgattggacttgatgccctcttttaaggagaga

gggacgcttcggagagactctggcgctccgagtcctcctcggctctcggt

gggaacggccggtggagcgggagctcgctgtgcgctcccggccgcgcacg

gtgggttcccggctctgcacctgatgcattcgggatgtctttcccaccct

ggcgcgctgcaagcttgcccagcccctcgcacgctcccgcacgcgctcga

aatgcgcacgatcctgccagcctgcggaccccaccggaatgcacaggagc

gcaccgctgcaaccagctcgcctcctccccgccacgctcccagcgtgttt

gactcgggcacgccggcagctggtgcgggaggccggggaccctgctggtg

acttcccggggatcgtgatcttgggtggaaggtggaggacccagagatct

cttctcatttggcctcccaccactcgcctccaggcttttgtgcatccttt

tctcccctgggggggttgcgctgccgggccggcctagcagcggcgtccag

cccgctcggaccttctcctttccccaggctctttcgctttccccctttct

tagttctgttccctttcttccaggcacctgactcctggggaaaagttgct

ttcccaagtttgccaaagtcctatgcaagtctcggcgggggtctctggga

acaaggggtgtgttgcaccatcgatcctcggagctctggcgggttagcgc

tggctttgcccagaatcggctgatcgctgcacctagggacggtcggatcg

cgggtgcggagccgccggttagggatagggcaccttgatttccctcaagg

cccaaaaggttcctttgagcagcgaaccttctttattttggcttctcccc

cttagttctccaaatctctgatctctctcccgaaagcacaccgtgctcat

gaagcttttgaggaatggatatctcaccgttcttgatgatgccctccgtt

tccactttagggtgggcaataagagtggccagccactattgttaactctt

tccgcatgagttttgcacctaagagtctttaagactcctgtcgagggtga

aggcaagctctgcgaagagtggctgagagcctagctctctccaccctctt

ccccaggctgtgggaaatgttggctacagagagacagaggttgggacctt

atcccgttaggtctttctgcctatgtcccaatggctgttcaaagttggga

gcctctcagtcatccagaacactttctgggagaggcctttgtgttccagc

cctgtcttcccttcttttgggcttaattcaccccagagatgctggtgcca

atgtcacttagagaaccatattcaatgtcagtttttctctttgtcgctgg

gcttgccaacacctgggaggcatccagtctggtgtgcactctcaaggctg

ttgggaacatactctcctatggtgttacccactttctcattttagagcat

tctgtccattggggggcttgtgggctgtgggtaaaagaggaggctgtagc

atcttgtataattgcatgtaggtgtacattcacgcgtgtgcacacctgtg

cacacgtgcgcgcgcacacacgcgcgcgcacacacacacacacacacacc

ttggacacatacagcagctttaaagaacttgtagttaaaaatgagttaag

gtagaatgtgatgtgatgggtgtgagggggagggtgcggtgtggtgtgtt

tgtcacagcctaagtatgggtctgccaatcctgtgcagccctgacttaag

cgtgcaaggacagaggccatagaaaccactggatgatggagaggagtcat

gtctaggatgactaggagaagacagcttcatggccagcagatctgggacc

accatcatgtttaacagccctggctagatctggttgtctcctaaggccgt

ctcagtgagccagaactagaaaaacattgaagatgagggcttgcccatag

gggtggacaggaaagccgaatgcttcttacttttaagtaagtttgtgagc

ctcatggtggacactggccttatccaggaaggctctgctctcaggctcga

agaatgcctaggattcattgcaatcatcatttcgctttatccgatggatg

cgtgcttcagagcagtgggggaaagagtgtggggaagcgcaaaccttcct

tttcctctcagcgtgttggaaaggatgatggtaccttcctaggaagccca

cccaccttctgctacccctcccccgcaaccaagcctctttctggggtaga

ggcatactgtagcctttgactttttattataagggacactggggaagttg

ttgtcacttgctcaccccttttgtcttctcttcccttcttctccagTCCC

CTCTGCTGGCCCCGACATCTACACCGCCCCACCTTGCAGAGCAGAGAAGG

AAAGAGAGCCCCATGCCTGAGCCCAGGGGAGCATCATGGATCTGACAAAG

ATGGGGATGATCCAGCTGCAGAACCCTAGCCACCCCACGGGGCTGCTGTG

CAAGGCCAATCAGATGCGACTGGCTGGGACTTTGTGCGATGTGGTCATCA

TGGTGGACAGCCAGGAGTTCCATGCCCACCGGACGGTGCTAGCCTGCACC

AGCAAGATGTTTGAGATCCTCTTCCACCGAAACAGCCAGCACTATACTCT

AGACTTCCTCTCGCCAAAAACCTTCCAGCAGATCCTGGAGTACGCCTACA

CGGCCACACTGCAAGCCAAGGCGGAGGACCTGGATGACCTGCTGTATGCA

GCTGAGATTTTAGAGATCGAATACCTGGAGGAGCAGTGCCTGAAGATCCT

GGAGACCATCCAGGCATCTGATGACAATGACACAGAGGCCACCATGGCTG

ACGGTGGGGGCGAAGAAGAAGAGGACCGTAAGGCTCGATACCTCAAGAAC

ATCTTTATCTCGAAGCATTCCAGCGAGGAGAGTGGCTACGCCAGTGTGGC

TGGACAGAGCCTCCCTGGGCCCATGGTGGACCAGAGCCCCTCAGTCTCCA

CCTCTTTCGGTCTCTCAGCCATGAGTCCTACCAAGGCAGCCGTGGACAGC

TTGATGAGTATAGGACAGTCACTCCTGCAAGGAACTCTTCAGCCACCTGC

AGGGCCTGAGGAGCCCACACTGGCAGGGGGTGGGCGACACCCTGGGGTGG

CTGAGGTGAAGATGGAAATGATGCAGGTAGATGAAGCCCCTGGCCAGGAC

AGCCCTGGGGCAGCTGAGTCTAGCATCTCAGGAGGGATGGGGGACAAGCT

TGAAGAGAGGAGCAAAGAGGGGCCTGGGACCCCGACTCGAGGCAGTGTCA

TCACTAGTGCCAGAGAGCTGCATTATGGGAGAGAGGAGAGTGGTGAGCAG

CTCTCGCCTCCTGTTGAAGCTGGCCAGGGACCCCCTGGGCGGCAGGAGCC

CCTGGCACCCCCAGTGGAGAAGCATTTGGGTATCTACTCGGTGCTGCCCA

ACCACAAGGCCGATGCTGTGTTGAGCATGCCGTCTTCAGTGACGTCCGGT

CTCCATGTGCAACCTGCCCTGGCAGTCTCCATGGACTTCAGCACCTACGG

GGGTCTGCTGCCTCAGGGCTTCATCCAGAGGGAGCTGTTCAGCAAGCTGG

GGGAGCTGGCTGTGGGCATGAAGGCTGAGAGCCGCCCTCTGGGGGAGCAG

TGCAGCGTGTGTGGGGTCGAGCTTCCGGACAACGAGGCAGTGGAGCAGCA

CAGgtaagcccctgccgcagtagccctgcaaccatgctctggcttgcata

gcgtttctttatgccaggaccatcctgaatctggggacttgactcctttg

tgctctttgaaaaaccacaccgaaccattcttctcattattgaggcatag

gcgggcacggttattttcagaggacagtgaggagaaatggtgtctagctg

gtaacatcagggaactcttggggtcagcctgtaggccctccttgccattt

tgcttttgggcctccatctggtttttctgtggtttggactacacttttgt

tttgcctagagtgactgtttaataataaagtgatgagaagaagggcagaa

agtggggttcaggctgagggtctcctctgatctggatctagccatggtat

cagtctagctttttctttgcccactgtcggcagcctatgatgaaagcagg

ggttggggatcgtttaagtcctgtatgtacccataggtctcaagtgtctc

ttttggcaggcacattgtgaggcagagaaagctttagttaaatttaagga

gaagagttttggtcttacagcctatatagcctcttctcatgccatctctt

atcataaaggctagccctctggcttctggcttcatctttgcaacgccctt

cccacttttgagttcttgtgatatgtttggtttaggacaggtgcttcacc

tggaattgcacctctgttacaatatcccccagaggtaggttgtgtttctt

ttagtaagacacagggaaaatgctgcggctgagttctacgaaggcaggaa

gatgcaaagtaagacatcctgtgagtgatggttccttgtagctacccaat

gaagaagatccttcctgcacaacaggttggggagcactggcccaagagaa

gggagttagcagtgggcttgccttttctgtttgccataaactacaagccc

atcattttcgccccgcgtccctgagtcggaccagttgcgcccacaaatct

ggttttctgttgttatggatgctaactttttacacaacttccggctggct

gatttgctgaccttgggtggattttccacctttgtgtgcactcttcctcc

tcctccattcctgttgcttatttaggtgggggaacttgagggggtgggaa

accgatataatcacctctacccgcagcgtgctccggaagcttcctgaccg

gccgagctgagctgtaccttatggaaagtggtactcagctaaaaaagtaa

acatttctttttccagagagtacagaaaataacaactgcggatatgtgtc

ttcttaggacttgagctgctcaaaaaatagactgatgaaattgtgcttgt

tttttgctcttgcttttcttaagaggaaacacccccctgaaccttgacca

gataggtctatcattccggatgtgaatgtgctgtgaaaaggggggtaata

tggaaagtcactttttaaggtcagtttttgggtgtgtgtgtggggggtag

agagagctctgttgttaaagtgcttgctatgcaaatgtgaggacctgcat

atgtaaaaagccatgcattccaacaacaccaaaaaaaaaaaaacccaaat

tcagcaaaatgcagctgcaatcatagcccaggggaggtggagatagactg

gaatcctggaactgtggccagtcagccaaattcacaagctccaggcgcaa

caagagatctcatcatacacacacacacacacacacacacacacacacac

acacatgtatatgtgtatgtatgtgtatatatgtacatacacacatatat

gtatatatatatgtgtgtacgtgtacatatatacacatacatacacatac

atatatctgtatatatatgtgtgtatgtatatacacatgtgtgtatacat

atatacatacatgtatatatacatgtacatatatatatatggatgtgaag

aatgattgaagaagatttctggctcccacaagcacacacacacacacaca

cacacacacacacacacacacacacacacacacacactcacaaataaagt

gttttttatctgattttttaaaagaaaatttaacttatctgcttgggaca

aggtcttactccatagccctagctagcctggaactggctatgtagactag

gctagtcttgacctcacagaggcccacctgcttcctgctcttaagtgctg

ggttaaggttgtgtaccacatgactggcctttttctgggtcataaatctc

ccaagccttccttacagaatcaagaattttcaatgagtccatgtacgtag

ttttctttgtactcaagaaggccacaaaagatgtaactgtcccgagagtt

ctgggttctcctgtgatgggtccctaatggaaagaaatgagggttggctg

agctgtgtctgagcggacacgtgcaggggtgagcatctgtaggtgttcct

agagctctctgagctcagtttaagggttgatgttgaccgaagtgacaccc

aaagtcactggggctgtggccaccatctagttaccatgaaagtgaagaac

tgcctctgtgtccttcattttattccagccttattcctgtatctcaccat

tggctctgggccctcgtgtatgaggttccgagcattcctgtgtgttggag

cccgtgtgaggtgatgtagactatgtccctgtcctcattagcttgtaaca

gtgaggcttgattctggagttcttgctgccacgccatgtgcttttgactg

agagtggaagtgagtccttcaccctgagcagctagatctggtgccagacc

cctccatgataagctctgaggtccgaggacatcaggagagggagaagata

tttctccgagtatgcagccctggctacccttggatagatgtacttcgtgg

gaggcctttgccgtcctccctccaggcgcttggattaatgtaccacctcc

ccatttcccactgttctgtgcctcacccccctcgcacctagcatcaatct

gagacatgttcagtggaagaatagtcatcacagccacgagagcaaacaga

aaggcaagattgaaaaatcagatgccagaaagccatggacttggccagga

tcctgacagggaggtctatgatagggtacaggctgcaaagttcactttat

ttaaagccatcaggttagcgtgttggtgcagatgaagcatctggcattta

ttcttgctcggggaagaagaaagggcagggtacagagaagcaaggcagga

aggggcttgttggttattgaattacagccataggtttctccataacttga

tcccaacctggggtaccctgaggggaacacctcttgactggacagagggg

taatgaaaccttgtggggggagttgtgtgtcatgatccctggatcaaatg

gcaggatatcaaagaaataaggaaggatggatggatggatggatggatgg

gagggagggaatatcagacacaagcaccacttggataaatctaatggact

tactaagtgacagaacctgaaggaagctagccagtgagaagacacagttg

agcagcaatcctctccaatttttggacccccgggtctttcccagtaagcc

ataataaaaggacagtgtatagagaggggaccctgtcaggcttctggaag

acgggactcttctgtcttttttttctctgaaaaactggatcagttattgg

tttgggtgtggtctaggatccagctcacctgatcctgagatgctcctgac

cctacccaacttacccagttgtcagtaacattacacgagcagcggtgttg

agccgtgtggagacccagtcctcgtttatggttgtgtctgcctaactccc

aggacagctttgattgcattctggtcctttggtgctatagaatcagagcg

aggcctggatcttctataggattctctgttatatcaaatactgaccaacc

ctcaggactgctcagtggctctgccactcttacttagttctggaagggag

gttcagacaccaaggggtggtcttggctggcttcctgaacccacttgtgg

tagagccctacccaaaaactttctggggcctgctttaggacatacgagcc

atgttataacccatcctggctgggatgttggggctctgtttgctcagctt

ggccttgctcacatggcagacgctgttttcagtgagggtcctatggccat

taacagtttgatttgagagaagatctagtgtaccccagttagctttgaat

tcacgatatatctgaggatgacattttttttttgaacttcagatccttct

gtttccgcctcctgcgtgctgggaatctcaggtgtgcaccaccatggcta

gttttaagtggtgctgagggtcagatccaggactttgtgtatgctaggaa

agtgctcttgcaactgaacatccctccctccctccctccctccctccctc

cctccctccctccctccctccctccctccctccctccctccctccctccc

tccccacactgtggcctttgaactcctggtggtcctcctgcctcagcatc

ccaggtacgagatctgagctctcatgcctgacttccaggcacctctgagg

tagcaccaagttccggtttggaacagggtaagctgtggagagccacagca

gccacattaataatatgtgatgctctattgacagactttctcctcaccct

tctgaaggagggggtggggctggcaatccaggctctgtgattacaaagtc

ggagaggctctgaaagctaaaaggttttgtgtaagtttgtagcaaactcg

tttagtggcaaaagctgatgcggggctatatttagcctgctttattttgc

atattcatatgtttggctgcaaaaatattaatgtgtttgattacagagtg

ctgccctagatgccactggagctggtaaacacaggtcccgataaggctaa

catttgcatagttctgatttctgaagtgcctggggggggcgggggtaagg

gatcgtgggctaatatcaagccccgccccaacttagagataaggaaaatg

gtgaatggaggaagtgacttgctggtagccacacagctataaatggtcaa

ggccaggctctctacttgccatcttgttcatgagccttgggcacactgca

gcagtccttataagcacatgcacggtgctgttctggctcagctccacctg

acctagtctttaggtaccagcaccatccctaaactgccagggttggaggc

ttcagccatcttccttgacgtcagttctaaaggtcaggtgtctccaggct

ttgcttagtagctatttacagctgctatgtcattcacagattcaccccag

tcctgttcaagcccgtttctgtttccaggaagtatccttttggggtgatg

catgattctgtaactaacttcctgggctcctggaaacctggtcttttgga

gaggtctggggctatgtaagggagtaagatggtttgccagggtgtgtccc

gctgtgttggaggccaaagctctgagttacatgtgttgtgaccccctttc

catcctgagctttctttttaagagtactttgctggtgaatcgtattttat

tccatggttttagattcctcattgtgggatcagacagccaaggtagggtt

agtttgatttctgtctgtctgcattgacttgtggtttttgaatttatcta

tcattcattcattcattcatttatttttagacaggcagggtctcactgta

tacccttggctgtcctaaaactcattttgtagaccaggctggccttaaac

tcacagagatattcctgcctctgccccctaaattatgggattaaaggcat

gtgccaccatgctcagcttgttgatttgtgggtttttttttccacttttt

aaacctgctcagggttacttagctagatgagatctggaatgaactcttga

cttttgtagacctgcatgttgtaggaaaatcctaacagctctttaaagcc

atcttatcctaaatggctaagctcagactctacttttgatttcttgctaa

tttggaaggaaagcatttccaattactatgtgtgtgtgcccgtgtttgtg

cttgggagtgtgcctgtgtgtgtgcttgtgtgtgtgtgcacgagatgact

ttaaaacatacaactgagagttaaagtgtaagacttaagagtcttgaaag

tgtaagacttaagagtcttgctaaatgaagatgagaggtgaagagagggc

tctggaatgttccacgataaataaaagttccgtttggcatcttaggtgag

gcgggtggcgcttctctagttattttggtagagtgtctacctccaacacg

gtgccttatacatagtgaatgtgcttgtactgactctgtgtcacaaggct

gattttatcatgtattggctgtgtgactggggttgtggtagttaacctct

ccaggcttgtctgttcatttataaaatgcggataacaggacctactcagg

gggtcattataaggagcaacgtgtaaatatttgccgtgcatggaaatgca

caatgcatgcgctccataggtgtctgccatttggaagccattgctattgt

atagaagaacaagcttgcagtacaggttcatggcttgctgtgccattcca

acccttgcttcccggagggtctgtctgtcgtccatgccttcagtactgta

aaactgctatggggtgtgtgtgtgtgtgtgtgttgggtggtgactgaccg

agtgcacctctgtttttcctgggaagtagactgatacactctggtgtccg

ggtacactttgtcccattgaccccgggccttgtgggccatgccagttctt

tagatgtagctgggcagtgctgcccaaatgtcaggcccaaatgcagagca

cagacgtcctacactctgcgtgcatagggctcagtgtgtggggctctctg

gtggaggggtagtcagcacacagactgccttactacgggcagtcttgggc

tgggctcacacgaaggaacactaggtgctgagagctctctccctaggcat

ccttcccatagtggtccagggacagtgttggatggttgtcacatgtcagc

ttctgagctgagtgctgtgtttagctcctagctggcctgttagggcagtg

ttagtctcacattcccgacggccaaggaaagcccaagaggttagcggact

tgcctatggtaagcagcaaaggatgctaggacccactttccctaaagctg

agggtccagctctggcctctcatttctcctggtcacttcccaggccagtc

acctgcaaatggcatagtcgataggtgttcaggggcccagaccacacaga

tccaggaagccccatgccacatcttactgacagagcaactggcctctaga

gatagaacctttttatgctgactagaaaacagaaaaccagagaagggaga

gaaaaagaaacccgttttcaaaaataaaaattctgcattgttaatttcat

gccagcagaatcagaaaaccccctttctactgtttccccttaaaagcccc

tgcgtccttattatgctggctcagtctgaaaaaaaaaaaaaaaaaaccta

tgcttgggttttgcctatgcgaagcttaactctgcagctgtggggagggg

ctccagccctttcttgtcggcatggaaactttggctaaaactcccccaat

cctgtctgaatctcagttctgaaatcacgttccactctgaccctgaggcc

tgattctcctcagtgtgcctagatcgtgttcggcattaccagaggaagga

tccaagttaggtaagaggaaggacttctgaaacagcacagcattgctggg

gaaatttctcaaagacctggctacagatattttgatgaagaggtaaaaac

tctagccctgcacgagttctttacagccaacaggagtccccgggctctgt

aaagacatacagcacacccatcggacatgctaaaaataagatccgcactc

catcaaaccgttatctttgcccaggacggggtggtaatacatgacacgag

ttaactggtagcatggatggagtaaggagttgcctacctaccttccactt

cccttgtgaccctagcctgataggtgagccactgagccccaagtcatcct

ttcctgggctaagtggattagctgaggttgtgatcttaatgccattaggt

ctgttggcacgaccctccttgccatcatgagaagcagatctggcctaacg

taatggccctctgggcaatccatcaagcccggagtacggggtgtgggagc

gttactagtgttaatttgggggagtcttttatggtgactgggaaagaaat

ggaaggatggttgggaatttatttattcaagccattcagcaggttagcgg

ctgctggggtctacagaggccaccccctcccccatttcttcaaggaaggc

ccctccctaatccctgaagccccggcctgattacttccagagttggagcc

aggacttagcaggctcttgtttctcgctgaggcctccttattaggagcac

ctggcatcataagcctctaatgggaaccagcgttctcctcctgcgtgacc

ggtgacaagtcgccctgtcagccggggctcggccgtgcgaggaagttctt

attagctttatggtcaggagggcttgtcctgttcaggaaatgaatgttgc

cgctaaaggtcctccaccctgacctcggcggtgtaacctgtgcgggcctg

ggagacccactcaggatgcttgggactgtagcctctctctgcttctcgtc

atatgtaaagaggggctcattgtggtgtgggtgcagccttgaacttttga

tttatgcagtgagagtgacttaaccctttctgagcctaggaggctatgca

gtaagtaagacccaggatgtgtatctagacatatgcgagcccttgtgggt

gtgtgcatgtgcacgtgtatgtttgtgcacctgtgtgcacgtgtgagtgc

atgcctgtgagtgtgctcttgtgtgcacctgtgtgtgtagttgggaaaga

agacacatcacatgtcattgggctgttaatggcagggagtgttttagggt

cttttgtctgtctgtctgtgtctgtctgtgtctgtgtctctgcctctccc

tgctccatccctttctcctagatttctcacaatggttggctgcacagtgg

cgagcggagaaaacgtggtgtctggaaatgaggatgggttcttctccaag

ctgtgtggacagggcggcgttcccagacgcttgtgtctgggtcaggaggg

ggaagtttaggagcatgccgtttggcaagatggtatcctggtcagtaggg

tcatggggcacacaacacaaacatgtagcccagtgcaggtgctcactggc

cctgggactgttggtttgaaaggacacgcacctctctttaacgtctccct

catgaagtcagattaacagagttaaagaatagtgtgcgtgtttctctcag

tacatcaaatgtgctcccctctgccccctcatagttccaagtccttaaag

caaggtctctctggttgtcacatggtgtcccccccccccaatgcctgagg

gaatgtttctaaatgcgacttttttttttttttttttttttttttgccaa

gaacagtggcgtctgacctcattcttaacggaaactttctgcctgcttcg

tttttaaagaaaagatatttgtgcgttgtctggccagtggatgttctact

tggtcactactgctggtttaaagtcctctggtgacaaaggaatactgcac

cttaaggggaggctgtggagtgtggcctagaaaagaggggtcagcgaagg

caaacacctccttgggaacctcacggatctgtctctgtttgtagcctcgt

tgatggcagctcacagaaaaaatacaaattagtgtttttcagattgtggg

gttggggacaaagagaaataacatattggtcagccacgtcgagtccatta

agcccagggttttggagactgagatctaagcctgtttcagggcccacaca

ctggaagcaaacttacttcctcttagacttttcttcttcctttatgtagc

aaaaccacacacatctcatgaacagttcctgcgcagcttggggagggtga

aagttgtggaagtcaccggccacctcggggtcagaaacacgcagtgtcct

cctttaaaatgatctgctaaaccaaggctgcgctaacatttgtgaatcta

cctccctgcatccattccctcgcccctcgcagagggactgaaggtggtac

ttaaatgcagtgggaagctaaatcaaaggagtctaaaaccacttctttac

ctgaagttccttatgggaaaactgactgggcctgtgctggctgcaggctc

tgtagggcttccagcacagtgggtttggggccaccactcactttttcata

attatcttattttactttctgtgtcctgacagctataagaacgtgtttgg

ttttttttctctccctttttgccaagttgcagataaataccacctcccca

agccagattcagtggtgtgtggggggtgcagaccagaacacatcttggca

ttccttgttgcaaaagtgaaaaatatctctgtttgtagttaattttagtg

gcaagcagatcatgttaactgggtgtaattacttccagatatggccgagg

gttttgtgtgttgggatgaagggcctggtaagtgctcacttcctgtgtaa

attagcctcttccattctgtctttcattctggccagccctggactgtgtc

attctcccgctctccccctcccagcctgcagccctgggggtgagactgct

tgctctgtccttccgagtgtaattcttttgtcacttgcagacacttgcct

tgctgagtttacacttggaatgagaagggttttaaaaggtcctcactggt

gccccgactgcctctgtcccacagataggtcctgagagtgtaccctagtc

atgcaggctgcatcttagcaatcagaacgtgtgtgtcagcgtgcgcatgc

tcacgtatgcatgcttttatgtatgtatgcacatgtgtgcagaggtatag

atgaatgtgtatatgtgtatatgtgcatgcacgtgtgcaccctccacttt

tattttttggggacagagtctctcaaggcatgaaactggctgattagatg

atcagattgactgtccagcaggccctagggatctgtctgattccacctcc

ccagtgccggggttagaagggtccatcaccacatccatttgagtgcttgt

gatagaactcaaatccccacagttgcacagcaagcacttttccaactgaa

ccatctctgtacccccttatttttagcggatttttttgtgtcacccacaa

ccctctccttttggagactccaccgccacccctgtctgtctgtcagacgg

agtatatcttcagatgtcctgggactcactatgtaaaccgagatgactcg

aaactcatgcctgcctgagtgccagggttaaaggtgtgcactaccatgcc

cagcagaaccatacggtcaaaagtctctggaagatgccaaacagtgaagc

tatagccagagacaggacttatgtgtcccatgtgaaaccactaatcttga

cccaagaagggggacccacgcatgcacagatcccggtcagaaaaggagac

ccacctgagccgtttctgccaagttatctgggtccccaggattagaatgg

aggagaagggcattcaactcaagttctcctgaactctgcttctgcccggt

ggccagcattcttggtgctgcttgcataagaggtgactgtttatcatcct

gcatcggctctactgataacccttgtggtccctggaacaatctactagag

ctagatggcttgataagggccacctttctccgtgtgggaagataaactgt

aggggtcaggtgaggggtggtgagttaggcgtagcgacgggtggaggctg

tcgatggggtgcagttgtggggaagagcttgaaggaggggactgtggtgg

cccttgggtggctgcattgatctcgtggtgtcccagtttcctgccctcac

ctctttctcccctgcttcatggaaacaattatagactcaactttgtgttc

agactggggcggtattggccattttttgcctcttctgtccttcccttctc

ccagtcttaaaagttactaagatgatgtccgcctgagctcttgtgcctat

ccccagtccactgctcttgcaggaagcatctaggcaaaggagaaacttct

gaccctctcacctggtcaataagctgcctgatactcaaacaaaagagttt

ggactggctgctaaaggcagggtacccagctgatagctttgttttgatat

ccttgtggtgtttgcagatcacggagtgggctatttagagcagggggtat

ctgtggaggcagactgcctgagaagcacaaagtccaagagattgagagat

agagtcaggcatcaagcatgtgtgtgtgtgtgtgggggtggtggtggtgg

tggaggtggcgtggggtgggggtggtgctgggctcttggatcagaagaga

cattttctggaaggtgatgcagtgctaattttttagtcactgttgagctg

gtgacaaaagcgtcgtctgtagagtgaggtatgagggccgagaagccagg

cactaagatttgctaacggtgtatctggataagctgcacagcctctcttt

gtctatggcctcgatgatgtgttatgtttcagatcctcagctcggggaag

gtgctggctggactggagctctagagttatagagcttttgaatgctgctc

ttttggcccaaggaagaggctgtgacaagaagtagtcgagttgtgactga

atggtgtcagccctgtgctagcagccagcgagtgaggtcataggggatca

tggaagtgcatcaggaatggaagggtccccgctttgcctggggctagggc

tctgttatataagattgtctagcacacatggggccctgggttcgaccctc

agctctccataaaccctagttcagtggtgcatggctgaagtcctagcagg

tgggatgtggaggaagaaagataagaagctcacactctgcctcaggtggc

atagtgagtttgaggccagcctgggctactttagatcttgcctcaaaaac

aaaaacaaaatctcccaaaataagaaacaaacaacccccccaaaccaaag

accgaaccaaaacaaaaaccccaaaactaaaacaaacaaaaaaaccccaa

gcaaaacacatcaagtagtctaccaattaaaggtgtctttgcagaaagct

agcattgtagagaccatatttatcctaaaagtatcttgaacccagcattg

ctgccaccactactgatgtttgggacatccagaggacttgaagtctacac

tgtattcttttgccttgccttggggagggttagcttagactttgggaatt

gatgactcttgggaattttgtgagtggtgaacactgtggtggggggccat

cctgggaaggcaggatttgggaggggctcaacccctgtaaatactgactt

cacttcccagcttagggagtggacgagctctggctaagacccctctgatt

actgtagtgtttctgtggttgtggttcagcctcctgtgagtttgggttcc

tgggaagtcacatggaatcacctcccccactcacacacacgaccccagac

acgcacagctacacagacagacacacagaaacagacagacagaacccccc

ccccccgcacccctacctgcaacttgggcatcaccaaataaagatcagag

ttgagaagaatggactaagatgtaactaaggtaatacctaaggcaggttc

agagcctggttttgaatcctggttgagccttctgggaaggttctagaagg

ccaaggagcacatgtgttcaaattctaggctggtacccagcacaaactct

atgtgggggtctcagttccctttcctaacagtgttccgaagtcgggatgg

gggccgtttgcttcctgtctgcagtggtgggaagcagcccctctgaacac

accttaggctggtggttctgtctcactgcagtgacctagcagtccggctg

ggcagggcccaggctcagcctgtgcgccctcccttgcattatttatagta

aaccccatgagcacagagtgctaaagcggaagggtgagtcacccattgaa

accatcgatgacataaaaacaagcaaaatgcttactgtgtgttttgtgca

gtatttcagggcaggttgtgctaatttgaacactcctaaactggatctgt

gaaagtgtacatgtgtgcgagtggggggtggaggggggggggagagttag

agttcagcctgagggaagctgggtattgaaaatctccttccagttctgcc

ctctgtcctcggctgccctcagatccctcttcaggttccagaggagtgct

gactcccttgctgtggttcacctgtgtctctggagggttagagagtggct

ggcgcggtgggggaggggttagactctgcctcatccctgtccctgttctt

gcttcctgcggaggaaggcacagttcctgctttacatggacatcgttcag

gtgtttggggtgactaagaatcatgagagcatgtaagagttgacaaggtc

ttttggagcttttatttcaaaacgtttgtttcaggaggagttactaagtt

ctagggaggctctgtgatttgccaaagaccacatagcaagctgtcgggca

gactaggactagaaagcagggctgtcactctctgtccccttgaattcctt

ctttccagccatatcagggcctgcccaggcctgcttcccacttcctcctt

actagaatctgacttgagaactatttcaaacctttgtcactgagcagggg

tcaggtgtgttttggcatagggacttgagtttgccccttccccctccccg

aactcccagcctcttttctgtcaatctgagttgtcaaatgtctgtcaagt

ctatcgttggtgaggggactatctgtgttggggtggggtgggggttgaga

tgcaaagactgctaagacttgggtcttgccctagagcttagaatttgagc

agagttaaaaagtatttggagacgatggttggtggcggcacacgccttta

ataccaacactcgaggcagaggcaggtggatctctgtgagtttgagcctg

atctacagagcgagttccaggacagccagggtgacccagagaaaccctgt

ctcgaaaaaccaaaaataaaagtacttagagagtatgtgttggcgtctgc

aatggagtggaaggtaacggaaggaccacacaggagagccatgtatagct

agatgagtcagcttcccttcgcactggagatgctttagtcaaaatgcact

gagttcatttgtaattatcactgaggtggaccttgcaacaaagtcccgtg

gggtcttgcttatggtgagaaacatttcacccggtaaccctacccagtaa

ctgtggtatggtaaaagagaggcagtcccagaattctggagagagtggca

gttgagtgggttgttgaaggacaagttgaattcagggtatggtggctcac

acctgtaatcccagcatttgggagttaaggtgggaggagacctgagctac

tgagtctaactctgtctcaactacctcttcaaaccccaaagtaggatgtg

ttttttgtttgtttgtttgtttgtttgttttgtccagagacatggtttct

ctgtgtagccctggatgtcagtcctggaactcactctgtagaccaggttg

tccttgaactctgcctgcctctgcctcccgattgttgggttaaaggcttg

tactgtcacatttggcatcccaaagtaggatttaaagatgaaaagtggga

atatgtggtcagacccaggagacagtagaaaaatctctcatctgccaggg

cccagggtcatcatccatgttggggtgtgtgtttgtgggttggtattcct

tggggctgccctaaatgttggaccaggcctaaagaaggcagagaatgaag

agggtatcagaggaaaccccctggacactcagtaaacccaggttcaaaat

ctgacccttgtagatactaaatccaaaagtgtcagtggagaattctgagg

atttcagcttatgtctttttttgtttgtttgttttgttttgttttgtttt

gttttgttttgttttgttttgttttcgagacagggtttctctgtatagcc

ctggctgtcctggaactcactttgtacaccaggctggcctcgaacccaga

aatccgcctgcctctgcctcctgagtgctgggattaaaggcgtgtgccac

catgcccggctcagtttatgtccttaacttcaacacacagcgcagggagc

agtgtaattgatcagcaaatgtaacggttgctcagtttgggctatatcct

gggatgactgggaccttgggaggtgttctgagaaggaccctggctagaag

agcccatactgattgtcacctccaccgggtatgccttttactgtccccag

ctttccccctatcttttagagttggcctgagaaggagttttgagggtaac

taatggtccctaaagagagttcctctcctatctgacactccaccagtgaa

agacgaaataaccctacaccacctcatctctgaaggcagagagccaagga

gctctgagaaagaggggcttcagcctgcaggatcagagaaatgacactgt

gacgcgggtcccagactgctaccgatcttcttttttagtagcaatgtgat

ggcccaaagttagactaacggtcaagccttgcttctcgcctacactgaca

gtggggcagagctggaggctatctgtcacagcccgttccgggctgtggtt

gtcacaccatgcaatgtctgcaaaccgacagtggccaggggagataaggg

cccccgttgtgtaatgagagccgtgggctgtttgcccgatggatctggcc

cctgggtaactgctaacctcaacggaggggcggctggcttggcagcgccc

aggcccccttgccgacttgcgggggcctttttgtggttggataaaggaag

ctcatatagactgcctgcagcgtggaggcctggcccacagggctggcaag

gttctcttctttgtcttttttgatgttgatggttgatggagagagggaga

gaaagagagagagagagaaagagagagagagagagagagagagagagaga

gagagagagagagagagagagagagcgcagagagcgcacacgcacgtgta

tgagcacccaaaagggggggacacagtttgtctgggcatctcccatttca

ttttagcataaaataaaatgaatgttgcaagctgaaagtcctctgggccc

tgggtccttgtaactcaggagctttgactgggaaggaagcatggctgaag

tttctagaagccagcagatgcttgcaggagcagcagagggggctgtgttg

ctgttccctccctcccttcatccctggatgcattggtggactccagcatg

tgtcctttgcctttataggagagccagagccagaccctcggcttcttctg

tgttcagtttcatcagctggccactggctgaacctgactgaggtggtcac

ctttttgtcagtttgtcacctgctccatgtcccccatcagagaggctttg

tgggtctctaagcaccttgctctatttccagggttcttgatgctccctag

caatgcttccagggaaggggccgcagagaccaggctgaagcccctggctg

tattctgtatttgtgtctttcctatccgggccatggtttcccttaaacca

tgatttcaactcccgttgttattctcacaaccctcttgtgagtgtctgaa

tttgtaaaacttttacatccctgctctcctgctctgatcacatcggggtg

gttatattccgtggcctaaccacatggatttgcccagtttagaagtgaac

caggttctcaccttcattgtttaacacattaggcttgcattaataatgct

ctcctctgagcgtctctttcctgttatttgtactgtgtgcgcttggttat

ttccgagctgtctgattgatccccaaggtggaggtgatatttttttttct

cccagaaagggtaggtatgttctgtttgttcccattagcgtgaatggaag

gtccttttcgttggaggttgctttgtgtccatcatagggttctggctcag

ggatgggcagggacaggagagtggcgatttaaaggatccagaggcagtga

ctcctattgattttagcttatctgaccctttagcttgaggctgcccttcc

ctcaaagtgtacaggctggctgtgagttagagcgactgagaaatgtaacg

tatgccatcccttttccttgcattaattagggctgagaaatccattattt

agagggatctcaggacctggaagaaggctctagagcttgctataaagatc

tggggccagctggaagattcccgggtagcagttagacagaggaggtaatg

ctgggaaggagaagggacaggtgccatgtgggcaggcaggcagacagaca

gaccctggctcttcccctgtataggggaaccaggctctcacctttactgt

ctccctgatttcatttcaaatattcatcaagatctgcccacggccagcta

tggtggtgcacacctttaatgccagcattcaggagacagaggcaggtgga

tcactataagttccaggacagcctggtctacgtagtgagttccaggccac

ccagtgctccacagtgagactctgtctcaaaaaccaatcaaccaaacaac

caactaaccaaacaaacaaacaaaaaaaccaaacaagtgtccactcaggg

atggcaactgtttggagaatcaccaggagagggcggacatagccgtggct

attcaagtcagaaaccatttttattgagcacttactgtgtgcgaagtggt

tgctcatgctgtagagaagcagaggacctaaagagatctaaactaaaggc

tacagcaagacaggaaatgacaactgtatgtgtcctggggcgtgtggtgt

gtgctgatgtatctgcggacacgctcaactattgcatgagtgaagctgag

gagggcagaggagaggggtcggggcaccatgctgatgggagacttagtag

ggcttcctggaataggtggtacctggctggcccttggaggatatctaggg

ctgtagttgtcatctgtaaacagggggtgtggggagggggagcagagatg

tggtcctgtgagtggatgcagggatggagtcacgtgctagagtggaggac

cagtgtggagtacagagcctctgagggagagtgagttggggccaggcctg

gagtgctttggctgctgcggtttgtaaagtgtggctggttggggttggac

ccttgctcaggatgctggatcctctgtggtcacctaccttccctataggt

ctagtggaggggctggctgatgaaggggtccttttggagacctgagcttt

ctcaggtgctctgatgtcacggaatgagaacattgctattagacctttct

gacacttaggggttttttttcatgtagcccaggctagccttgaacttgtt

acatacccaaggataaccttgaatcctgatccttccattttcttctcctg

agtgctaattataggtgtgaaccaacacactcagtttgtgttgtgaaccc

aatgcttcgtgcatgctaggaaggtactcttagaactgaccacaccctca

gtcatgaatggagacattcaactggccacacacacatggctccttttcat

gatcactttacaaagatgttccacggcagagcaaactcagtcctgggcct

tgggagagcaggatttggactgaatagctttttgtgaatagcactcatac

ccacttactggcttatggctacctgaatcatcctctttctgtgtttgtcc

cagtcagctttcaagacctggcccaggcaggttcatctcagctgtgccac

caccctggagctagggtcctccctcttttctgtttccggcagcagttgga

gcttacaccatctgcccatgatctctgctgccttttctcttgtctccttg

tctccacatctctttcttggtagtctcatggtttctgtgaatgcctctct

aatgaaagcaagtctcctaatattagttgttttttttccctctgtgtgtg

tgtgtgtgtgtgtgtgtgtgtgtgtgtgtgtgcgcgtgtgcgcgcgtgtg

agagcacactccattctccttgcctgctcggcatagcagtcctaccagga

accagacagatgtcagtgtctttcagacttctgtaacactgtcactctag

agtcttgtctgccatcatttgtgtgtggagctctgtcccatcccaggcac

ttatcttcagccttgggtattttcttgtccagccattttgccttctgtat

ccaccatcaccttcttcaagccatctccttaccctcaggtaagggcaccg

caggtcctgggctagttgccctccctcccatgtgctcctccatatccagc

ctaagtagtgctgtgccctagagcttttgagaaactagaaaattctttgc

tcagtgttcaagctattggtgagcctagatcccagctgcatgtatactga

tccccattttctcggcctggattgtaagatggccacccctcagggagggg

gtcacctttttgtttttctttttgcatctgtgcaacagccctgaaatcct

cacctgcatgtccttcttcatgcctcgctgtgtggtgcaggcctccatgc

caagcatgcatactaccacctccttcagcagcccctttctaatgcattca

tttctaccctccactttcaagtgggacccaaaccccacttgggactgctt

tatatgacacagttctggaaggctggccttcagagggagccagaatggtg

tggtgagtactggctggttccttgctgtgattttcctggggttcattttc

taggcattcttggaaggcaaggacttgggctcagcacctgctggtccagt

taaccctattcctgtttgcagcaagcttgatccctccctccctccctccc

tccctccctccctccctccctccctccctccctccctccctccctccctc

cctcccaagacaaggtctcatgtagttcagggtggcctcaagcttgctgt

gcagccagagatgaccttgcattttagatcttcctgcccttaccttccaa

gagaggttgtaggtgtgactgactggtttccattccctccttgtgtggga

tggatatgggagagttcccgagtagagggcaaggactgcgggtttgctct

gctccaacactggtagggtcgtggattcgtgacccatgatgtaccaagta

ggttgccttggggagagaggaacctccgattccaaaggtcccaggaagca

gaaagtcttgtctacgctgcctggagaccttccctgtccacctcagacta

gaacagtgggagatgaaggctgtgagtaagatgccaggacacagggcagg

agctctcagtgcgtgaccactagggatgagtctgcacccagccctgccag

aagccatttagagcctgtgctttgtgaacctgatgtccgtgtaaactaga

gagagagaggagaggaaactgggtggagtgtgtgagggggtacctcctcc

tcctgtctacacccaggtggaagatgcttggggctgccctgaggtgacca

gtcaggcttcagaaagcaaagtggagattctgggacacttggctgctctt

gttccctgctgagatggttcctgtaattaagatgggaggttcagaaaggg

acactcagagctcagctggtccatggtcatcaaggcaagacaagtagcat

tactgctttgaggaccattttatggatgtgaccaaacctccccctgcccc

ccacgaccagcacagtagtcattccaggtgactctttatctctgctgtgc

tgcttgctttcacagggtggctgaaagggacaggagtggcctccttcctg

aaatggtagattcatcttgtcataccagtgtctgagtcctgagagtatgg

gagcacattcttgttttattctcagactcctagtaccggggggggggggc

agccatttgaatctcaatttctccgacctgaagcctcactggcctaacct

tggaggtctttggtgagtaggaggtgtctgttttagccataggaggttca

tcagtcctcgggcacgatctctcatggtgtagtgcctggttttctgctga

tggaaaggtggtttcgagacctgtgtagcttaggccatgtgtgggcagag

cttccaaatctttggttcagagttccagcctgttgggcctccagtccagg

ggatctcattctcttggtggttgtgtgggtgggtgggggctctcctttcc

tggtcatgcccaactggaccctttggcatctccgttgtcccattttctgc

aagacacaaatggccagaccaggcccttgttttttgttttgttttgtttt

gttttgttttgttttgtttttctgcagcatttttatgtccattggaaagt

taagagaaatttgttaatgactttgttatgttcccctcccatcctcttct

ttcctcttgtggagctggttataaaaccgagagccatatacatcttaatc

acgtgctgtcctattgagccatatacctaatggttcagttagcatagtgg

gcccaagtttagatccgaaacacaaaggtttgtgcctataatcccatcac

tggggagatagagatagacatgttcctaaagctcattggcctgtcaggaa

agaaaccaaagagctacacgttcgatgggagactgtctcagaaacttgag

acaataattcctcaataatcaaggaaggtagtattgacctatgatcttca

tatgcaagcacatacatatgtactaacatgtgaacacgtacacatgtact

ccacacacataaacagacacacaagcatccatacacacagacattttatc

tgttttattttgtcttgagatagggtctcactaaattgcctaggttggcc

tggaactccctgtagcaaggtgtgccttgaacttataatcgcctgcttta

gcctcatgtgtagataagatcacaggcttgtgccaccagacccagctgaa

tccaccccctctgtccctctctctctcctttgtctccttctcccccctct

ctaaatttaagatttcgtttgtcttctctcttccccttccccgagcagaa

ctcaagactgtctggggggcgggggtatggggttgggctaggggctgtgc

atcctcctctctgaccatatacacagagcagagtggttttaatgaataaa

taccaccttctcatgcctttgtcagtctacctgcctcctctagccatgaa

cttccgattcttagtctgtgtggggcaggtcttgtcggggtagatgctga

tggccatggtagaggtagatgcggatctgtaggttgcttggggaggagct

tcctctgttttatgagtttggttgctacttcattgtgatggtttttcact

tcgaatttctctccccgtttccctagccccttgtgtgacgtggcttctgt

ttgcactcgaggcgtgctggtgcagtttcctctatgtgtggccatggctg

agagtggactctttggtttctgcagtctgctagacaaagggaatgaaacc

cactgtcttaccttctcactctcgccgaagtccagcagcaggctttgggg

atgatttttccctgggatgatcttaagtcttgaccaggccatgtactgct

gcttccttggcaccctgaggccagcctgggcttcagggacctgagggtca

ttggtgtctggtgggctgggtggaatgctagtctgtgtctcagttccatt

actgtggagaaaagcaagacaagcaggcctctttagctcttagtaccctc

ttaccagtcctgatcttggtgaaagggtgctgtatttgtgcacttaggtc

ttccagggctggctacaaagagtggggagtgtttctgggcaatttgtcct

aacaatcagtttgggttgagggagaagagccggtaaaagaaaggcctgga

ctgtctggcagatagagaacctggtgtatgtgtgtgtgtgtgtgtgtccc

catccatgtgtgtgtgtgtatccatccatgtgtgttgtatgtctgtatct

gtgtgtgtgtattgtatgtttgtgtgtatccatccgtgtgtgtgtgtgtg

tgtgtgtgtgtgtgtgtgtgtgtgtacagctctggctactcttgactctt

cctggtcaggacattgagtagggctgatcctggtcctgcctttgctggaa

tgatgggattcccacccccaaccctagctttgttgcatggtctgggcata

tggacatcttcactgtgcatggaaataagcactagaggagattccatctt

tctctcactctccccatggatagcagcttactcctcatggcccttctccc

tgtccctgtgttgtcttttggtttagatttttttctttgtacattaaatc

catctgcagcactggaaaaagaaaagaaaagagaaatcatactagctctt

tgggacacgtctcatgtataagggctggttctttatagagtcatcgagac

ttttttttttttactttctgtatttgtgtgtttggaatagtattgatgtc

agtagctcagctactgcactgttgtctggttgaacgcgtcagcataggca

gtcataaatactgctgtttggatcttgtggtaaaacagaaacccagtggg

tccacttatctaatctgaggtgagggttcactcaaacacagagggaggtt

taattcttttggttaataagggacatatgccgtccctgtgggttgcaaag

ccgtggctgtctgggagatatccatgcctctaggggtcagtgggaaactt

gggtgtgcaggcagtgggagggaggcctccagatggggccaccagctcag

caagtttccgtgtgggcttaggcaccgttgctagtaggcccctcggtgcc

tttgtgtgaattagaaaaagtgcccatcgtgtgggaggatgtggttctta

gggcctgggacatgataagatggacctctctggatcttacatctttttgc

ccaccttcccatcagacacctgagttcccgggcccgagagtcttttgtgg

ctggagagttcctgaagtaaaaggtgttttaaaacgtgagacacttttga

actcttgagtttctgaaggttgtggtcttcagaatccgtttttgataatc

cctcagccaggttgggccctcaggtaaccaagcagtttctctagggcatg

cggcaccaggctgcaggtcactgtacctaggtgtgagcaagatgtgaggc

atctctggatgctcaggtgtcccaagccagcctgggaatgtcacatggaa

atgggggcatccttttagcatgactgtctctcgtgaccaaggccaagaaa

aatagcacagaggtaggagtggtgaggaacgaagacaagtgcccacgtct

cacagcctcaggcaccacagtccttaggctccgcctggggagagcacagg

tcttacgtaatcagattctcacttgacgataatcagcaatgaccaggaga

cctgttctttgcgcagattttgagtctttgcaagaagatccaatttccct

tcatacttgatttgtctcaagtctgtgtcttgcatgtgtgtgtgtgtgtg

tgagagagagagagcaagagagagagagagcaagagagcgagcgcacaac

tatgtatacatatatgtggagaccaggggttgacatgcgatgacttttcc

aattgttttcaatattattttttgagacagtatctctcactgaacttgga

gcttgcccatttgtctaggctgtctggctagcagacgctgagattctttt

gtctttaccaccccagtgctgggatcacaggtgcaaccaccacaactttt

tgggggggggcggcggcggtggcggtggcggtggtggtgggtgctggaga

tctgaactctggtcttccctgtttccatatcaggggcttcactgaccggc

catttccacaggctcatgtttgggtcttttaaaagctattgctgggccca

gtctgacgggtggatggacatacagcacacacagcctgggctgctatgcc

acagcatacttgggcactgagctgacaagctaccaaagtgcccatgggaa

gtagaccaggcccggagaataatttccatttaactaattattattcttac

tccaatagatttgaaatctatttaacttttcagggtctcctagcctttta

agtgtttcaagtgcactctctctgataggttacctttcctctccctgtag

gctataattagtgtcctttacacaaaattggcttagaattggatttggag

aagataagaatcaaattaccagtatttatcttgtaaattccaaaagatct

acttaagacactgtattaaaaaaagagaaggagagagagaggaaagagag

agagagggagagagagtgggaggaaggaagggagggaggacaattttgtc

ctaagaaaaagcaaattccttgtggtctgaacaatccatagacattttaa

tactgtatttccaaatttcccatgtatttccaaaattcccatgcagaaac

ctaaaacctatgcctctttttttatttctgttttgttttgttttgttctt

tttttttccctcttcagttgaacatctgaaatatgttgccgagctttgaa

cagtggagagatgtggtcccagctttcagtaaaactcggggagtttggct

gatctcttatctctgtcttgtggtgagatgtgtaaacctctcaagtatct

taaagagataggcacacttcttaaatgaaaaaaaagttgtgtgtatgtat

gtgtgtgtgtgtgtgtgtgtgtgtgtgtgtgtgtgcacgcgcgcttatct

ctctgtctctctctgtctctgtctctctctctttgtgtgtgtgtgtgtgt

gtcagtcagaggacagtttggcaccggaccttgccttcccctttgattga

catgtctcttgctgtttgtggctgtgtagaccagactagctggcctgaga

gcatgcaggggttctccgagatccatcatcccaccccgggtacctcttca

catgtgtatggcaagtgctctccctaccaaggaaatagacgggctttatg

ctagcatttgaagttaaaaccagtggcaccagatcttggtgggggcgtgg

ggtgtctctgtgtgaccatttaccctgtaaacatcacggtgttcctttgc

acaggccgtggagtttgaagatgtctgtgttagagaaaacaccagcattt

acagcactttctcaaaggtaccgggtggagagggcccgtggaaagcgttc

attagcaggctcaaaatatgctccgtactccacacgtcttttactaaatt

gctccagtggcgttgaactcttttaagttttcctctgtcaagttaggtaa

ggcgaagtccccgtcatgcggcagtggtggtgcgtctgtgaccacctacc

ttctgcgtgactttctggtttagtaacgtcatcgagtttcctagcctaat

tctgtccttttttggttttgatgagagtgttggataccaaagagagaagg

gggaaaaaaaggcaaattctttaaaaggaacaaaaatatgttttgtgagc

aattctacttctggatatctacccagaagaactcaggcaggactgtgaat

agaacttggacgcccctcgtcacctcagtgctattcacaatagccagaag

gtggaagcaacccacgcatgcttaccgccacatgggttcgcagacagact

gtgtggaatactattcagctttaaaaaggaaggacattctggttcgttga

atcccacagatggtccttggaggcgtgatgctaagtgagataagccagac

acaaagaacacatattgtatggctccctgaagctcagataccaacgttct

atgacgtaaactcatagagacccaaagtagaatccagacgagaggagtgc

gcgtgctctgtggggaccgagcttcagttggaaggcttatgctgttggtt

tcagagttttatgcatgtaccaaatggcactgaactgcgcactttaaaac

agttcagaaaacctccctattttatgttgtgtgtatttttactatcataa

aaacatgtttgatgctaattgctacacctggtgcttgagacaaaagtttc

ttagaaaatacttagggcatcatgctataaaaaaatcctcttgctagttc

acaggagcacataggcatacccccccccccaacacacacagatgcacact

ctctcccttcctgtttgttagttatcaccctttctcatactgtcctttaa

atgtcattgttattagcacatttattattgttgttggggtttttttttgt

ttttggtttgtttgtttgtttttgtttttttgagacagggtttctctgtg

tagccctggctgtcccggaactcactctgtagaccaggctggtcttgaac

tcagaaatccgcctgcctctgcttcctgagtgctgggattaaatgcgtgc

gccaccatgcccagcttactactgttcttaaaagtcagtgttttgccatg

tagctcaggatgactttgaattaactgtgatcctcctgcctcaacctccc

aattgctgggattataggaatgccttgccaggtctagcagatttaaagca

tttgcttatggtactggccttgttcgtttgtcacataagcaaatgtggca

ccctgaggctttatccctccctccctccccacccctccctgagaatagca

gttttagttgttttggggtgctgggggttgagcctatggtcttgcacgtg

ttggacaagcattttaccacccagctgcatccccagctcttgctattttt

tggccagttttatcagatatgctggttacggcatttgaactgtcatttgt

gtttatgaatagctgtcaggcaaagcgcgcaccataggaaagtgtgttat

ctctcaggccacactgaataggtacttgacctccaatagaattctaattg

gatttctatatttactccttgttctggtattttttaatgaaacatttttg

tttgcttgcttttgttttgtttcgtttttctttctctctctctctctctc

tgtctctgtctctctgtctctctctctctctgtctctctctctctctgtc

tctgtctctctgtctctctgtctctctgtctctgtctctgtctttcagac

agggttggtttctctttgtaatcctggctgctctgaaactcactctatag

accaggctggccttgaactcagatatatgccagcctttgcctctgagtgc

tgggattaaaggcatgcattattacctccccccacccccccccaccccgc

ccaggctgttttgctttttgaggtgaagtcttactaagcagcccaggcta

gcctgaaactcaggaatctgcctgcttggctttcctagcactggggttaa

aggcgtgctgcaccagctcagcccagcccccccccccccaagtaattaaa

aaggacattcaacccagtttgcaaagctacacgagaggtttaggagagtt

taggttaggcccctttgtgagcaggctttttccgggcttacattcccctc

ctgtgatagggcttacctaccctacaacctgttgcaaatttagcctgaga

tttttgtgagtttcagagcagaataaactcccccccccagcttttttttg

ctctttcttctccatttgatttccacttaaatgaaaatgtaaaaactccc

caaaaaaggcaaagcaaacaccaaattccccgaagtagttagaagggaag

ggtcccctccccagcctccctgtgcccccaagacacggaacagggagcag

tctcattctgggcttctggaggctcaatggccagcatgtgtaggtatggg

gctcaccatatatatagtaccaggcgtgtgggcgcacttgtggggaatct

gaaaaaacatcatttggcatttctaggtttattttaatcttacaaccttt

aacgccagacacatagctgctttcccacttaatgtaactatacattgatt

gcttttaattagtgttggatctttcttagtgtttcacagcttgggatata

taaaaagtctgacttaaattaaaagaagaagaaaaagaagcagctaactc

tttgacagtctcttgagtctgaagagaagccagcactggagtcctaacct

ctctgcctcttattcttgagttgtgtgggcagtggagccctggaagaggg

cagagggcctttcctcttcagaacttaattaagcacctgtattgaaataa

aggtggggaaggggcccccgttttcatagcctagccaactgatgagcgat

catgctggagaaatggggtcttgaccagaagaaaggggtggggtcttatt

ccttcaacactcagggactgctaaggctcatttctggtttctgaggcagg

attctgcatgtttgctcgaaagcctctgtggccctggcgaattctccagt

aaatatttcagacatcctcgggcctgctttcctctccctgcagtaaatta

catattaaagtagcctggtaactgagcactggttgctgtgagataaggcc

tgggaaatcaatatgtttgccatgtggctgcttgtttatgacacctgcct

ctagaacagttgagcaatgttctgtttgttaaggctgcatttcagaagcc

ggcatcctcctaaaacacccgagatcttaacggggagagagacgtgctgc

acagatatcgattttagtttttccaagagtagaattaagcttggaaacag

tgctcacagcccatgaacccccccccactcccctggccccagagttgtta

gcacaaagggaggtggatcctctgtcttgtgtgtgtgtgtgtgtgtgtgt

gtgtgtgtgtgtgtgtgtgaaagttatttgtatataagcatcttataatg

ttacaatgtggtcagttgacagaaggcatgcagctgggaatacagacctg

caggtgttgcttagattttttccccttccacttctgttacttccacccct

ctgatcttcctggtttaaaataagaaaatagcaactgggcagtggtggtg

gtacatgcagctgtgtgtgccctcaggaggcagaggcaggcggatttctg

agttggaggccagtctggtctacagactgagttccaggacaactagggct

acatagagcaaccctgtctccaaaacaaaacacccaacaaccaaaccaaa

ccaaaccaaaacaagaagcccctcaaaaccaaaacacctaaaacaacatg

aaaacacagaaaaatgagcacaccaacctatggctagtgaggtttggcct

acccgcccaccctccctttctttctcccgaatcagggtctcatggaggcc

acactggtcccagattcactttgtataccatggatgaccttggacccctc

cctgtcccctgctccttcttgagtactgagtgataggtgtgtgtcaccac

accagatttatacagtgctagggattcaaccttcatgcatgctaaacaag

aatgaaggtcttcaccaagatgtaagtctggttgtcctgaaactcactct

gtagaccaggagggcttcaaactcagaggtcctcctaccgctgcctttac

agtgctgtgattaaaggtgtgccctgccacagctagagctggtgggtttt

aacagtagataagactagctttgtgactgaatctactggtgggcaatact

tttgtttatttgctttttttttttttaaataaatcaggtcttgctgcatt

gatatggctggcctggaagttgtgtagtctaggctagccccaaacatctc

aaaatttaccatagtcctcctgcctcaggattacaagagggaaccatcat

gctccccaccaggccaggcttcttaaagccattgaataacagactacctc

aaggaagcaaaacgttagcgtgagtgtgcaggcacggttgtgcttgtgtt

ggagggtgagagtgcatgaggctggcagcttgggagagaggaggatggca

gcctgggtccttaacacttagccttccgttttgtaaaagagtagcatcaa

tacattgcctcttcttcatccaagtgactgagagggaactagtgtgtcta

aacaaactgcgcgggagccactggatgccaggcataaaacctgcattgtg

ccggtgtgcttgggtgtgccagcggtacacaccccacggtgttccttacc

tttgtaagaggcatgggcttcctctctcctgctccccatgctctgtcact

cagacaaacagacaggccttctcggcatcattgctgtgccaaaaagtgag

tttttaaagagaagcacggcctcctcacagagcatgccggaggcagctgc

cgctaaggtggatgccctgaatattcagtctcgtggacgtggacggcggc

catgatctcctaatgacactaatgtattctgaggtctcatggtggtgttt

gtgtgtttttaaaatccagattggcaggtttaatcaatagtggcaaaatg

tctcttttctttctactttccacgggatgggaggtggcttatcggatcct

ctctctgccgatgcagagtccaagatgacgcaaaaaataggagggagatg

gattgaggtaggagagcttggggagctaggaattaaagaacacaccctct

tccttaaccactggacagatccctgttcctccatattaggcatggtaatt

gacctatttaagcagatgaatgtatacttaatatttttgtctgaattttg

tagatctttgcctatctagatgacaaggtatttaaagtaaggttgagtcg

gacgcgtatgacacactccatttcagggaactgcagtcctacagtgatat

tggggtgtcagcaagctggcccctttcttcttgcatctactcagggaatg

ctggaaacagatggaaccccatgagaatccttgtggtttcagatttgttg

aggcttactgcgacttggggaagtgatctccttctttctctgttgggggt

caaaccctgggctctgtgcctccaacgcaagcaccctttcagcccagcta

catgcccagcccacactgatctttccgatgcgcccttttatgagatgtga

cacactgtgcttcatggaattactaacatttcacgaaactgggataatga

tcaactcagagcgaggctggatgatgtcacactctccactctcagaagag

ctaccttgggtacctagtcagaacacaggagtgtgggccaccccaaagtc

cttttaatgagatactcacatgggcctgaacttggggggactcagaagac

accagcaacaaggaacactatgaattgttttttgttgttgtggtttgggt

tttgtattatcttctgtgccaggatttggaacgcttgcctctgattcagc

tctctcccgacttttgacagctcgaccgtgatacttgcgactggtttatc

tgcttaaccaaatttcttttagagtcgtgacggaaatctgtgactgaaca

gggcagcactgagctacatgagtctaaattttggagtcaagatgagtctc

aggcaagagcttggctgctttggaaactgcatcctgtatcttacccctca

agcatcttaatgtcagactgagcttgaatgagactctacccctgtgagaa

gggcatgagatactgtctaactggtgaggtcattgtggagttcacatttt

aaaaacacatgtacatggactggatctatatacatggctcatctattaag

agcgcttactgctatgctaagaatagttcctcgctccgataccagacagc

tcatgcaactgcttgtagtttgtagttctggtttctttgggtccaatgcc

cattggcctccacaggagccagaacatacgtgccacacagaaactcattc

aagggtgtgtgagtacgggttgggagcacacatacaaacactcaagtaaa

caaatttcaaaccaaaaatgcatgtgtgtttatattcctcagtgttactg

tagtgcaaggtcgatgttagttttccaaatcttatcttgacagcagaaaa

tctgaattcagttctttgtatttttcatactgtggcacaaatggagatat

atatgcttacttgtttcttaggatatatatatatatatatatatatgtat

gtatgtatgtatgtatgtatgtatgtatgtatgtatgtgatgaaggaggg

aatgctgtgcccctgggagggaggaatgggggtaaggctccccccagtgc

aaagccttttcttcgcaaacctggctatgctctaccaaccagaaatctgt

tagactgacccattttccctgtccacagcctcagcatcgaacaccacttt

ctgctgggatggtcctctgggttctagcgttggtagtgaattcaaagcaa

aggctaggacagaaccattcacagacccctgagacaagaccccagctgcc

ctgtccggaggactctccctggcttttcttttcaaagggaaaaacaaatc

cttaaaacgtactcagaggaagcattttctccgttggctagacgggactg

gggagttgctggcagcattgggctgtggtctccggagcccggagagctgc

tattcctcatgtgcacttggctttttatgcatggactgtcctccagctcc

tgggcaaatttctcactcccgggttttcaggagaaaggtttgtcatccgt

catcaccctcctcatccatcacagcagacggggaggcactgcctacgccg

gtgggtaggtgagggaaaaggttatacgtagttttcctttcttccaggta

tagctctgtcccgccaacccccccccccttccccctgcctcccactttgc

tgagtgcataggcctgggcttaggggatcattctggtgtgtgtaggcaga

gggatgtgttttgctgttagccctagttggtcttgtcatcccgtggtgtg

gttgagtaagaggtaaacagccatttaattatttattgggtgcctggagg

attttataggcggaaaagcttagggcagtggaagaaaaggggggccaagc

ctagagcttcttgtgtggctccctttttgatttgagattgacacattaaa

aaaaagattcatctttgaaaaattctttttgtgtatatttctgtatgtgc

acatgtgtatgggtgcctgcagaagccaaaaaggggtcccggatcccctg

gagctgaaagtacaggcattagctgtctaatatgcgttctgggaactaag

ctgtggtccacaggagcagcaggtactcttagaccccctcctccccccgc

cctgcccccccgccatcgctccagctcaggccgatgtgttcaactcatgc

cggcatgccaagtatcctggcacgggaatgacggttgaaccacctacagc

tccctccttgctgatcaatcccacccaaagtaggcactctgcctgcccac

tgttaggtcttgcttttctccatggccggagtcaccagatggcacgtcat

tgtattgatcattattttcactgtgaagcacagcactgtggttattgatg

gctagaggctggggctaagagaaccttgaagccattgagagccttctccc

gtatggtgggaagcaggcatgagagtggagatggtgtggagtccaggctc

agcttacaagatagggagaggggaatgagacatgctcagcgagtacttac

tgccagacagacatcgcctcaacagtctgctgactcacattctcttaaaa

agaaaagaaagaaaagaaaagaggaaaagaaaagacaaacatttctcttt

agcatgttttgcttgggaggattaaagaagcatgggtggctgcttttttc

aattgggccaaagaggctcaacctgcaagagagttttggcgaccacatgt

tacagccagggctgcctttcttctcttctctttctctgaaccttccagag

agcccaggcccctatctcttaggggattaactccttgtggctcagggtcc

ttgatcctcccccaccggcatcagtatctaaccctctctgcaaacaggat

gttttctcggcctcttatcagatggcacggggagatgcgtggccaagata

gaggtaacgattaactcatcctaaattcctttcacgagctgaggccccag

accgacttaaccactgagcagagggaggccttgacaggagcaaaccagat

tgggcccttttctctgaaggaggtgtagggggctggctgtggggttgctt

actcctgcagcacccccacccacccctaccctcagccccgcccatgggtt

cccagctcagaaatcaaagagaaattgacaagccactctttactgagtgg

gggctaaataattgaatttgtgggttaaccactcaggtttctttattttt

ctcactccttgtgtcttcttagagtagcactaggactaaggggtccagtt

tgtgcttgtttctggagaggcaggaggaatgggtaggaatccatttaggc

agctgtgtggtcactttctaaagagcagccttttggattgcagagtagtt

tagattttgttggtttcatcccagttgtgcctgtgtagaaaacttgagac

taactctattctgtttgcagttaaatgtttcagctcgggaggctgacaag

acagaatcggaacagggagtggtctagcagagttttacaagtagacacca

agcaagtgtgagcctgcagacacaggctgctaggctgggagggacccaga

tacctggggaagcatgtcagcccttatgtctggcccccttcatttgagca

gttcttacaggttccaggccaggctgtgtctaccttgcttgccactcccc

aaagaaagaaagtagttcagagatgctggtccttggcactggactctgtg

cagaccccttctatttagtgaccactggccagcacaccttttagttatgt

ctgaagccacagagaaaggcatgtctcttatcatctggacttcagaccaa

tctaaccatgtggaatgacaagatgaagtccatctaggcaccagagagtt

tctcattgcaggttcccttgagatcaaggctggttacccccctgaagctg

tacttcccgtctggtctgttatttgcagagccaggtcctaattctaaccc

agatcttagccatctacccacacggaaaagaaacatttgcaaagaagtta

gacctcttggtgaatttggcacctgtaaggtgaacacagcggtatgggta

gaaagccatttgagtcagtcaaccttagtttaatcttgttatctgaatgt

atttcaggtttttataaacatgtgagctaaggctggccagtcagcccaaa

actcttgagttaattctctggggttgaaaccttgggtggggctgtacctt

gggtggaggctttttttttttttttttaaagaggtgtgggatatctatgt

aggtttcaattaaggcctagatctggcttgtctctgggtttcttgtttgt

ttataaactgtgcttcggtgaggtttttttccacttcccctcccttacca

tctctcttccttctcttatcctaacagtctcaatacttctccaactgtca

cccggggtgctgtaaaagaggcatttggacagcagttctgcagggattct

tgtcttttctcagagttaacacaaagggattgggggtagccccatcacca

ggggggagacggtttttcttcatttagcttttaattgctttctttcactt

ttatgtataaaagattgtccatttaattaactcaagaatgcaatttcaaa

accactaatgctttacacatgatagcttttaaaataaaagtagcattcta

atattgcataatgtttttctctcctaacacatctccctgcgccaccctcc

accccgcccgcctgtattaaccttttaacagtgcaggattttagaagaaa

aggctgtttctcttcttgatggggggtatctctgattccactgtcttcat

taaaagaaaaagataactacattttgtattattattatttttctgtgcgt

gcatgtgggtaggcgcacacgccatttgtcaccatgagggtctgaggatc

aaaaacctcaggttgtcaggcttggcggcaggtgcctttgcctgctaagc

catctcatcttggctgactctcctctttcctgatggaaaagagatcgggt

tccttcttatctctccttactcccttatcgctttaaaaaatgttgtgaat

gtctcaagcccttggcattttttaaaaaccatttttgattaatgtttggt

ccttgtccttgctagtcttgcctgcggcctcttcctcttcctcctttcct

tcctcctctttcactttcctcctcgtcttcctttttcttctccctgcctc

cttttaaggcaagatcttactctaggtccagtctagcttggaaccattat

agacaagagcctgccaaccttcctttcccgaatccacccccagtgcccca

gtcctcggataacaggtgggtgccaccgtgcctacctcgtctaagatctt

atagcttgctcatggagcccagcccagtccttctacctctgacctccctc

aatggttgtgtcttacagccagacttgggaagggccagggtacagaggga

gtggaatcttggtcttcttgcagggatcccgggtgctgaggacaccacct

tgctttgctttgcttttttaaaatgagagataagcccatggagtccctga

caggtcagtgacttaggccaagttgtgctccccaacttccagggttcagg

tgggaatcccatgacataagcgctagctggagcacctggggctcagcatt

ccacttgcctgcagcagctgctgcttatttctgaggatgccaagaaaaca

acattttaaacctaaggacacacgatgaaacctttcaaatccagggctgt

tgtaactcttgtcgtcttctgtccctctaggaggggacagttgttgctgt

gggtgggatccaccttacaaggagccctgttctcctgtggtgacttttct

ctaaagtgcatggcattttccagccaaccagtgtggtcgccatgctgtat

tttctcaagaacgattgagcagctagtagacgtgactaaaccgttccttt

ctttcactgcggccggtatctggccagtgctttccctgtgctgggtgata

cagatggccttgggagactcctgtgctttggatcttgtaccgtgatggca

aaaagggacccggaacaaatgttttctgacgaggatcgggactatggttg

ggtctgtgggggtagctaatgacagcctacccgtagagcatgtttttgtt

gctattcaggttgttgctgtcccctgcttatagagggcggattttcagat

taacctggtatggattcgatgagagatttgagagtcgtggtttcccctga

agcctgtcctcctcagaatgagatctccatctcctctcacctttgttttt

acagctcttgctgctttttccttagcaaacagtcactgtagaaagggggg

aatgttgcttgttttctgtgagccaaaaggctggcgggaaccctgtgtct

tggtcattcatcagttcccagctttaactcactgagctatttaaacttgg

gtgtgtaaggtagcagctccgaatgggctttttggtgccagctttagctc

tggaaccccaggttctgacagaggagacacagagcaactgaatgtacaaa

accttccctgccattagaaagctgagtcttgtgccttgggaattagaata

caccatctccagcctcttaggtcctttttctttaaacaaacaaacaaaca

aacaaacaaacaaacaaacaaacaaacagcccagtgtgcacatgtgaaat

aacgtctctgactgctatggtggccacagcatctgaattttatggactta

atgaccctacacttaccagcaggtctgtgtattgctctctgcctcttgat

tttggagggagttcactttaatattatggtgaaacttggtgctcctcctg

agatacatgagccattaggaaattgccagcggcgtttgatgaagggtcat

ggaagaagtggctcctttcttccttagatctggagaggcggccacccagg

gcctccaaactatgggatcagacacgtggcttcattcaggtctccaggat

aggcttcagcatcctttgtgacagatgagaaacttcatttctactgatga

gaagttcaaagtggttggatatttagcttacgctttgaagctggtagcta

gaattgagacttggggttttttgattgcttagtctatccgtgtaatgcat

gcttgtattctcccttggtattgagatgggcagactagtccagggcaggg

ccacctttatcatcagaccgtggttcttctttccccgagttggtagcagt

ggggtgcagactagatacagcttgcttgccactaagtagcggcctcaggc

atccttttttagagagctctgttaatccgaattttaaactttcagaaacg

ttcagaatgagttggagttcattaagaacacattttaaggtagactcgcg

cacggagagtaatagcaagaggaactgggggaaagagtgggacagaccca

gcttctcaagagtcgcacttccgtgatttagcagtagctgtgcatgcagc

ttggccctcttctgaagttacattgtttttaaggaccactcccctggctt

ttctgaatgagactataggagggtctctgaggtacactggttaagattac

ggctgtcagggtgaaaccatcgtcacagagctgtgtgtgaaggagctaag

acatgccttttactgtgagtgaagtccctagtcttgtttgtcatggtccc

agagaacgatagcttacagctgggcatggataaagcccttacttgatccc

tgggcctgaagcacgtttattactatttcaaaatttgcatttggactatc

actatataaaaataatactgggagctggagagatggcccagcagttgaaa

acactcgccgttcttgcagacgacagcagttcggttctcagcacctacat

tgggtagttcacaacggcctatgactccatctccaaggaatctggatctg

gcaccctcgggtggtgtacacacacacacacacacacacacacacacaca

ccccggttgggggagagctcccaacacatatacataaatagttaagagtt

tagggagatggctcagtggttaatagcacttgctgctcttacagagaacc

caagttcagtttccagcatctacatgatggctcacaactgtctgaaaccc

caattccagagaatttgacatctcctggctttcaagggcactagatgcgc

tcatggtgcatatgcatatagatatacagacaaaacagtcacacacataa

aagtaaataggtatatccctaaaaataattaaaagaatgttcttttaggt

aagcaaccttcccagcaaattgtgttaactgtgctcggattcttgactct

taactagactctgaagggggaggttccttgcctttcagaagtccccgtat

ctcttgttctttgtatcccttttcagtttgcttcatcggtgggcctgtcc

cactacggctgagggaagagggcagacatatcatgctaacccacgttcag

cttaacttagctaaaccctgcactttaagaggtggctcgggcaaaggaag

taggagagattgagcactccctcatctttggcatgtctcttttcttctcc

agtaaggttcctcggtcctgtcggtagctccccatctaagtgtgacaact

atccaaggaacgttgtttcttggctcacgggtttcttttgccctgtggaa

aatggctttctagagtcgtggctggcttgtggtgaggtcagactgttgtg

ccttgaggtataacaccatcgacttagccaggtctttggttagggaggct

atgtctgtctctttctctggcatttcagactctctgacccagcccgtccc

tcatgactagcgagaccctcgtttggagacacttgggacttgagggtgat

ggaattgcctgaggccagggttggggcgcacagggacacccagatgtttg

catagtttctccattgccctggttaggaatgtatttcctcaggatgggtt

ttagccttgaagtgagccgaagataaacatggagactgtttcacatccga

atatcatggggagagtttgagagaaaggcttaggagtgggcgggaaggtg

gggggagagaaagggcagagtaggggaagagggaagcccaaggagtggct

tctgagacacagcagtccagggcctgtggcttccactgctttcagcagaa

aataggcttcatagagatgactcggcgtgcgttggactgattctctctgg

gagtggcacccaggcagtgtcatgtgttggggtaaaatatggaaatcctg

gtttagttggctgagtgattagaatctttagggaaactgatgagtctttt

aaaaagcccgtgtgtgtgtgtgtgtgtgtgtgtgagagagagagagagag

agagagagagacagagacagagacagagacagagagagacacagagagag

agacacagagagagagtgacagagagagagacagagagacagagagagac

agagagagagagagacagagagagagaacatgtgcttgtcatacacaaat

acttcagaaaagcacacactcagggacttccaagtccttcagctttatct

cctcataagaaacacttttgaaaaaatgatcatttgggggagatgggctc

tcagaaatcaggggcagagtaagtgtggggggattgtggagggtatgtgt

gagtgctgtggaggtactttgggctctttgttggggacccccaatgccat

agaaaaccagtctctcgttctggctggcttttcttccgttttccttttcc

tctgggaagggcttatgcttcctccgtgattctgagctacagggtggtct

agaaaatgttcacacgggacaacctacatgcacaccccaaaacatgaagg

gcttccatggttttcattccacccggatgtcatcttctcttcctagggac

tcagggagcactctggagctggcagagcccagggcagtagactttcaggg

cttttgtcagctcatatgaaagaatgtgtggagatgccaaggctgctggg

ttgatttcaccgacacagactctaagcaaagagatgggcatcagggagag

gccgtgggcgtgggaaggggccgcagccttcctgttttctctcctgatac

cttcagatcttgatgctccagtgtgccaaggagaggagagatgtaaacaa

ttccaattgcctccaggatgcaacatctaatctaaacatgattaagctga

tttgattttatagattggaatttctaagtgatagaaacggatgaggttaa

tgtgaaatatttacttaataaaaagcctcattacctggtgctgaagggta

cagggatggagattctgggtaacgcgtttgatcttttggaaatgaagtta

gatgcaagttttcttggtaaaaactgaattctgtgatcttgtttttacac

atgctgtgcttaaatattaacaccaattgagcaaattgccttttaactgc

ctttatttgctttcattaacagtataatttaaaagctcaagaatttgcat

ggaacctgttgtgcatgttggggggggaactattcataaattaattaatt

gtgagaacattatcttttaaggaaggtcaccagacactaggccagtcttt

gaaaagcagtgacgtgtgttttattagtttttggagacatcatgtacttt

tggcttagagggggaggagggaggggggaagaagggagagagggagggag

aaagagatgtggggggggacacttggctggatcaagaaaaattttaatga

ctgggcatgttttaagttaagttgactgaacctaattacactgtagtttt

agagagctaaaggcaatatcattggtatttaagcaccagtttgaaaatga

aagccaacctctataattggctgagttccctaaactctctatctctgtct

cccctctcaaccctcttcctgtcctttcctcactcctagaccaatgggaa

gagggagaccctgccagcactgcccatcatcatggcatggtcctccatcg

gggacagctcatgggactgttcttgcacgaaagcactgtaaatatcttga

gatctgcccggccccctgacctcagttgcttccttctggacagatgttcc

tgtggttgagaaccttggccaaaacaaacgctatgaatgggatgaggaga

ccactgtgtcctagggaggccaggagccctgcctcaaggtcctctggggg

ttatttgacacatgctgttctgacacccctgtttggacaacacgtccctg

tttcaccctcccttcacatgtggcctttctcctgtcttctcttggtgctg

ttggcggttttcctaattaacttaatctgtagtctaccttttctgggtct

gcagatgccagcatgctgaatttatttattttgagtgtttttgttggcta

ctgcgaagacacatttttcctcccttcccccctcccccagagcccttaga

ccctctttgagaaagaatcccttgttttcatctgtcagatgtgttatttt

taggggaaagagatgaaaatattttaaatagtatatattttaaaagccat

ataaaagttaaggaatacaggggggaaaaatgggtaaaacagacccgcct

ttctcccttctttccttcttcttttcctttcctttcctttcctttccttt

cctttcctttcctttcctttcctttcctttcctttcctttcctttccttt

cctttcctttcctttcctttcctttcctttcctttcctttcctttccttt

cctttcctttcctttcctttccttctttcctttcctttcctttcctttcc

tttcctttcctttccccttccttccttccttccttccttccttccttcct

tccttccttcctttcttccttcttcccttccttccttttgttatgttctg

tgttttttttgttttgttttgttttgttttgttttgttttgttttttaaa

gggaattgccatgtagtctaggctgaccctgagcccacagaagtccttct

gcttaagttttcagagtgccaggattacaaacttgagtctccatgcctgc

tgtttaggtatgagtctgtcacctgcccagtgcataggaaagtattttcc

tggtctgttactttacagttttgattctgggtcattttatataacaatct

tactcatttaacgcaaaaagtgtcacaatgaggtttgtagaatgggtaat

ataaagcccatgatgatcacagtttagaattagaatctgggaagaatatt

tggatgagaggaagagaaggatgaaaacaggaggagggagtgtgtgtatg

tgtgtgtgaaagagagagcaagcatgtatctgaaccatgtgtgggtgaga

gagagagagagagagcatgtgtgcatgtggtgatatgatgtcaaaaggtt

gttttctcccataaaggctgtccttttttgaggacccctggctagaattt

agcttgcatctgaagagtgtgggtttccatattctgagacccgttcttga

gctgcaggtctagaaagccactccctcccagccttggggccttgctgaat

tgctgtctctaaaatgtttctgagggtacctcctgctttatgccaacaat

acatctgtgaatctaaccctcctcctttgaaggctacttgccatctcttt

tctttgggctctccagctgtctctgccagccatatttcttacccagttgg

ttctccactatctctatcagctagctgtaagtcactaagataccagtaag

atcaactcatccatccttctctcctgctctctcttcttccgaagccaaca

ctgcaggtattgggccctgagttaggtttagatattggcttggaaaccag

ctggagttgtgggaaggaggaaaggatgagtgccaagcgagagaggatgc

tagagtggtgagtgttgtgctcagtccctatgcttgaccccatcattacg

atcattttacagaggtgagctcaccagttcctctcccatatctgcctgca

ctttgcctagagttcggtcatgtggacaggagagtcagtcttcactcttc

gaggcttttgtttggtttggatcttgttcaggaacaaagaaacaataaaa

tacagacattctgcaaacgatccttctgggttatcttgtcactccgtttt

gcctgcccattttgtccttcctctgatcttggcacagttattcccagagt

tgactctatggcaagtgttaaggtagctatgttggctagatagatatcca

atttgtcttgagctgaagcttgccttctgttcttctgttcgcatgtacat

ccatccattcactcatctagtcagtcattaacaaaacgtcacacaattaa

tagtagccagattaaacatgttttatacatgtttttacgtgtatgtgtgt

ggatgtctatcatgtgtacgtatgagtgtgggagcacgtggaccgtcaca

cgtgtggatgccatggtaggcaggcagcagtcagagggcagcctgtggaa

gtctagcatgagttctggggattgaatcaggtcatcaagttttggtgaca

agcacctttgtccactgaggcatctgcctgttcctctttactttgcagat

agagttttgtgtagtccaggatggcctcactatgtatccaaggctagcct

tgagttcttaattccttcttctacctcccaagagctgggattacagaggt

gtgtgccaccatacccatccagctaatactatttctgtcaccatagtatg

tgttcagaaaataaatcaaattctattctttttacaagtaggtaaattta

aaattacctggctgtagtagtgtctctgatacctctttacctcaaggtaa

ggggcgatgcgtgagtgctttttgaaagacagaacagtggatgaagactg

gaagttgtattttcccagccaggagaataacaggttcaaagctggtgtgg

gctactgagtgagttctaagccagccttgtgtacttgtgaggcctgactc

taaaccaaaactacagaggtagaaatgttgttttggatcacagggcatga

gagggaaaccatctggtcggctagagactgggagcttctgtctacacaga

taattgttcttgatttttaaattaaaaagttatttgttcaccaggcagtg

gtggtgcacgcctttaatcccagcacttgggaggcagaggcaggtggatt

tctgagttcgaggccagcctggtctacagagtgagttccaggacagccaa

agctatacagagaaatcctgtctcaaaacaaaacaaaaacaaacaaacaa

acaaaaaacaaaaagccccccaaccccacaaaaaagttatttgtttattt

atttatctatagatgtgacttcattcttagcccaggctggcttcaggact

cggggctcatctgccttggttgcccaggtgctgggattccagacttgaga

cacccacacctagtttgagcttagtttctagacctgttggttgtctggtt

attttttttaatttcttgtaattcacttagatggtgggtcgtgccttggc

agtggggttggctgtggctttgtggtgggtggaagcccgtgctgatggca

gccacagagtttctgagtttgtttcagttcttatcggaatcccttccagg

aacagaacatactttccactgcatggggcagaggttggagtggaagtcac

tgtgtccctggaatgttccaggccagagctctctttttctgtggatgtct

gtgtgcagacaggacaggctctcagtgcccagaaacactgcatccagggg

cagccctttcctttcgcatcccctttcttttgtttggtgggagggaggaa

agcaggttgtgtgggaagaaagtttgtgtttttcacacccctttgattcc

tctcccccaccacctgaactctgaagcctttatctccatccctgggaaaa

cccctctagtgaatgaaaccattacttctatacacagaagggagggaaga

gagtagtcgggactgtgtgcaggtcctcaaacagttaaagtgtttggggt

ctgttgcctctctagaggtcaaaggtgagacagactttttgttggcggcc

acccccccccccttttaaaactccttttccttgttatctcaacttccaaa

tgtcagctttgatgtctattgttggaacttcaagaggactcttagcacat

cggcaggtgttggttttagtgagcgggcggagagttccacattcttgggg

gcaaagagatggatcccagcacattcctaccccaagagcaaagctcccgt

tgccgcgcccctcccagggtgtacttgcagaagacctctggtcctgatag

gtctggatagtaggtggcactgggttccttcttgctctccaggcctgtcc

aaagggatctgtcttcaagctgtggagcagctgaagggaggtcgtctaac

aaccacgtattaagatttcattaatcaaagacacttccatggctctttta

cagaaacacaactactttggctgtcctctgctgacagcccgataatttgt

ctttccccgaagaggggaggggagagggacagggacagagaagcatacag

gggtcctgctcacagaaaaggctccccatgccaattcttggcatgcacac

gtgggttcatagttgagtttagcgatctgcactccagactcacagataca

gtcaaagcagtgggcagccagccacatccccttgccccacggcccacatg

gtaagccatcactccaagtctaatactccacacacaatagatgggctggc

tttgccttagtgcaaggaagtgcttacccctgaatcccagctccacgata

tgctgatattgggatgaccatatagatgtgtgtgtgtgtgtgtgtgtgtg

tgtgtgtgtgtgtaatggagggtcggggccaggggaggggtgatgggaac

agtgggacctacaagctactgatttggttgtttttgttgttgttgtttgt

ttgtttgtttgttttttgagacagggtttctctgtatatccctggctgtc

ctggaactcactctgtagaccaggctggcctcgaactaaaaaatccacct

gcctctgcctcccaagtgctgggattaaaggtgtgtgtcaccaccgcccg

gcgggagctactgatttgaaatgtacagtcttcccgttctactttacaga

gcggttcacatacatggaggcatctgaccttctgtaactcttggtcctgg

atatggctatatgtacatttaattacctcccagtgtctccagagtaggtg

atagaacactgcatatgctgctgctgctcccaccatcaccccccctttaa

ggtctctgaaagagagagtagctttgaactcattttgttgaactcatcag

atattttccaaatacccactagatacatttgactttgtcgagagacctgc

aggtgagggaggtataagttgtttcttagctctttgggggctagaaccat

tacacaattagccaccataaaagatgcaatttagcgaagtcagtgggggc

tgcaggaaggacaagaccctttccatctggctgaagtgcccagcgagcat

ttatttagtcctgcctgtgtaccagtagagatctgaaaatctaaaaactt

ggttcttgtctccaagaacttaattatgtagcagagagagtagccatgga

agtgtagaagagacacgggtggtataatccagggggaaatgaagaaagaa

tgatgtatggtggaggcagaatggaaatagcattgtgtgtttgaggaggg

gctgtggacaagtgtactgggatgatacctaagcagggttctcaaggaca

gatgggagaaactgatcacccaggagggaggacgttctagaagagacagc

atgtgcaaaggccctgagggggacagagaactgaactctggtgtgttcac

tgtggctccagaggtggagtgaggcagagagcaggggatggaggtgatgc

tgagcttgcctggacgtctttcgtgcccagcagagaggcttgcactttcc

tttatatgtttggagaaggcgttgaaacagttttatcaaggaagtgaaat

aggagaaagagtgtcctagttgggacggctctgagagctgacttgaggat

gaggatgtggggccataggggcatcgctgggaagccggttgagcagttcg

ggtgagccaaatgatggcagtggcaaaggagatagggagaggacaatgga

gaatgggtcagagggttggcaggtacagggaggtcgtagagagtggacag

agagacttagaagctgtttgcttgtaagtctgtagggagataaggttgta

gatgttctgggtaggaagacactgggacatggtatttaaggacatcttag

aaaaagcgtacatgagggcctgtatacttacaacaggtcttgagggagag

gtgtgtgtgtgtgtgtgtgtgtgtgtgtgtgtgtgtgtgtgtctgtgtgt

ggtatgtctttctgtctctgtctgtgtgtgtgttgtgtctattcgtatgt

gggtggtgtgtgtttttatggtacggatcttttagtggggtatgtatctg

tgtgtggcatgtgtgtctgtctctgtgtgtggtgtgtatgtgtgtttgtc

tctgtatgtgtagtctgtgtgtgtgtgtgtgtgtgtgtgtgtgtatgtgt

ttgtctctgtatgtgtggtctctgtgtgtgtgtgtttgtctgtctctctc

tgtgtctctgtctgtctgtctgtctgtctgtctctgtctctgtctctctc

tgtctgtctatctgtctctctctttctctctctctcagttgctcttccac

ttattctctcactgaatttggagcttcccagttcagcccagctgccacta

ctggatttccgtagagggcccctacacccgactttttacatagggactcc

cagtctgaattcctttctcaagcatacacgacaaacacttttaccaactg

atccatctccacagcttcaggaaagatattttgactagaagaagacgtca

gcctacaaatccagttgtattcactgacttcagatgatattttcaagtga

atacgaacagaagtatcttaattccacagcagaaaatggtcctttggagt

tgcttgaatcatgagaagtgttttcatcaaagcctttattcatgggtgtg

ctgacatctggggagcggagatagatgttttcctgaggtccagactcctg

agccatgagctcgtccctcgatcctaattaatcctttgtagttagtgact

gatgaaatcacggctctatgaaattttaatcctcgctgtgtgtgcgcaaa

ctgactttatcacaggaccccaggtgtgtgtcagaaatgagtagtggaat

ccgctgcatatttgattgccttgtggaatccactggatctagagaaaaca

gggtggagcacttgaaggttgttttatttctgtggccttgcctaacagtc

aaatgtgatcatggccactgagagtgagcagttagctataatagcttacc

cgaggaaaccgtgagggcagggagacagggtggctggaacctgggccttg

gcatctgataagtcttcctctcccctttgctgggaggcaaggtgggagaa

tgagcggagtgcgctcctgatacaaggagggggcgtggctctgtgatgtg

acagtctgctctggcttccccactacctcaaaatggtgagctctgcactg

gaatagcaagaatgagggctgtacacgctgctcggagggtagctggggtt

tggatgcatagttcaggtcggcctttgtgacatagcatttaggggcagtt

gtatagctgggcatggttatgttcccaggggttacactccatcactacca

tccccctgtggcaactctggaattacctgttggatttctcaccaaggacc

tctaagtcggcatgtgctctaccagccctgtcctttcaagagaactgaca

ggcggatgcttttgtgcagtgtgggattcccgaagtgtagcgttggaagc

cttggtgacagtctcatggttcctctgcgactagtggaaagtgtctgttt

tagggggtttgcagtgaagggaggtgagtagctgctctcgagcaccatat

atgacagtccccgtacaagggctttaaaaaaaaccagattccgagatctg

aatacaggtgctagacagaagctcaggatggttatgtgcctttcttggtg

ttacacagctaggaagcagtggggctggggtctagattcaggggtcctca

ctctttccactgctatggaaggaatattcatgctctcctggcctctccat

gggagggcagttatcacagctaggctttgccactgcaggcagacctttct

ttgcaggaggatttctcggaagcagattgcagttttctttctttttttga

gacagggtttctctgtgtagccctggctgtcctggaactcactttttaga

gcaggctggcctcgagctcagaaatctgcctgcctctgcctccagagtgc

tgggattaaaggcgtgagccaccacgcccggctcagacaaacgggctcag

attgcagttttctaacaaacgggaaggacagagtctgctgaggagggctg

agcctggaggagggtgctctaggtggtctctcaggaccttcccagcagag

aactctgtaaggggagaagtgaaccattctatctgatcttgtaaaagtgg

ttgcctggggctagtgagcatctgctgacactttccttgcaaggtttctt

ggaggtcattagagccacaccaggcctctctctagagtgtatacaggctc

ctaaggcagagccagtggtccagacagggagggaccaggaacagagcctt

aggcagatggggagggacagagccttagtcatgaagctgatcgggtacaa

gaccagcttagaccagatttttctagctctccctcacccctttcccatct

cccctctactttctcccacactgtaaagccattcatcaacttgacttcac

cacattctgtatcacatccttggaggtcacagcttcttgcttagtaaccc

tccagtgcagccaccgatacaggagagtttctcttcttactcatcccaag

cactttgggtttctccccatgttgccggctttttggttcttctccatgcc

ttctcttcattcctttggatgctttcttttaaatctggtcaagctgtggg

gcagcagtgtcatattcagagttggaccaggatctgttgaggctctgaag

ggcttttggtaagcattgtagtctgtgatggttctctctcaggtgatgtg

gtttaagtgagacaggagaggtgttgtaaaaggtccctgtgcccttttca

gccttctgttcaccttaggactctctgtccctctcctaggaagccacata

gcattactctttccatggatattgcctccattggcaggcatagttgggca

accaagctccatagttgaatgttttgaaagaaaacaggagcacctgtctg

ggatgcccagttacagtcctgtgaaatacccctggctcgtgttgagagaa

actacagaaccacatggaatctttgggtaccgaaagttctccacatttca

tggtcctgggaagggggttgttcagcagatgtctttcttaggtggcatct

gttgactgatttttccagggcagtgaacattcttgacttgtaggggtctg

ctgattgccatgttgcaaatactcttgatgtagctaacttaaagctgccg

attgtgggcttgctaaggcagtctagagttatgagtccagttttgtaggt

cggagcctctgactgccaccatcaaagccctgacaaccctagatgggcac

caaagtggagctccagcccttgctggcccagtaggaagatagggtgttac

ctggggaaggtcacttttcctcctaaccaaaccacaaaattcaaacctgt

atcctttgtctttgcctttgctttttctctttctggtccttggctctggt

gagcaccatggaaatcaacaaaaaccactgctcattgctgacagtagttg

gttggttatatctgtccacagtttgatttgctaatcatagccgtaatcct

agagatgaggagtgttccatttctgttttatagatgaggaaaccgaggca

caggctagagaagccattccttcacagtcggacagtcttgatgtcagagt

gaagctttgaagtcagcagtggctgatcttcacatcccaagccatcccca

gtgccttccattgcggcattgcacagacttgtatgcatttcgaccttcct

cccaagacaggtgcttctagcatgacctttgcttgtgttgggagctcttc

atttcggactgtcacatggattctttgaggtgtgaagtgtcatgactccc

atttagggcaagtttggctccggggttcgggctctcctctagcatggttc

ttaagccccgagtccacagatttcacagagactgaaatggctgcttcctt

catagacactgggtttgcctgttccaaaaatggtgatttctatagcaacc

atttattctagcagatgtgtgcctggtagggttggcaagaagggtgggat

caaaaatggaaacggccttttattcttcacttcctgagcgcgtgccctgt

atatgaggcaaacagaaggagaaacatggcatccttttagcctgtgtaac

atatagggcccagcacaagaggatctgttgcttaaagctggtttctgtct

gtagcttaagctgggctgtgcttggtggtggaactttctttggcaggcgt

tcacgttggttccaattcagtgaacgtctgtcacttcagtttctgcaaat

gtagacatttatcatgaggtggttaataaatgcggagggcacagtagcta

aagatcgctaagtctaccatagtgtgtactagccaggctctctggatgta

ggttgtaggttgaattctgtgcatacatttgtctagctgaagccaaataa

aagaatctgttgtcataaaacaagagtttcttggagcccaggggcaagaa

aacagttctcccttgaaaggtgagagaacagaattaccatctagctgttg

gaaacgttctttctagtcctgctcagtttatgcatcctgcttaattttta

tagttttttgcagagtgaatccaagaatggccttcccatgacttccaggt

gcacaggtgacaaggttaggctcagctgtccatgttttctagaatctcag

actctagagaacattctgtcctctgcagctgggctaagtgtccacacttg

gccagttgctcagtgaggctttgtagtgaggaccatgggtgtggtctgct

cagactctcacaagagagctgcatggcatgacttatccaagagaaatgta

cttgcaacccgttttagtggttggatttctgaggtcagagtgctgaaggc

ttcctacagggtgttccttctggcttgtcttccctgtgtcctcaacggat

ggggaaagagttaagatctctctctctctctctctctctctctctctctc

tctctctctctctctctctccctccctccctccctccctccctccctccc

tcccccctcctcctctctctctctcttgttttttcagacagggtttctct

gtgtagccctagctgtcctgtcccggaactcactctatagaccaggctgg

cctcgaactcagaaatccacctgcctctgcttcccaagtgctgagattaa

agtcatgggccaccactgcctggcttcttttggtctcttgagatctcctc

ctcctcttctttttgagatatgatttctgtatgttgtctgagactgagat

tatagtcatatgatcatactacaataccaggctgggaagggggacgtagg

gttggagggtgtctctttttataaggacactaatgttatgacctttgttt

aacccaattgcttccttgtaggtaagtcgcatcctgtggtatttgaggtt

aggctttcagtatactaattttaaggatatacagcttagttcacagcagt

tctatagtttaaaatggctgctaggatcctcccttgccttttgtggattg

gagggggtttacagtgtcccctaaaaggagtctaccacaaatagaaagga

tgaatgtgctacaagaaaatcctggatgccagattggagtctcaactatc

ttctttctcccctccttcctccccccacctctcttggatagtctgtagcc

ctcactagcctgatactcactgtgtagtgtagctaggtttgcacttgcaa

caaacctcctgcctcagccccctaagtgctaggattatagttatgggtcc

ctgtgcctggcttcaacctcctcaattgcactgcatttgtagctaatagc

tatgttgtgtgtgcacacatatacatatatatgtacatatacatacatca

aacatgtacccacatgcatgtatgtacacatgtctacatatctatttgtg

tgtgtgtgtgtatatatatgtgtgtgtgtatatatatatgtgtatatata

tgtatatatatatatgtatatatatatatatatatacacatttacagaaa

tatataactgtttagaaagagattgtttacagatgcctttccaacccctg

ttccatactgaaaccagctgacctgtttgttttcctataaagtttgaaat

tgagtaaagtacatattttttactatcacagcccccatcattctcacagc

cccctccatcattcttgttgagcaacacctgaatttgcaattctgcagtc

ataagttctgtgtgatagaagattccccacaatggccttaagataaggtt

tttaatgaaatgagagaaagggggacagagagatggggggaggaaggagg

aagggaggaactagggaccccagactcttgtcaccactagctaagtggga

atatgggcaccgggtcgttccagggagcctcctgtggcatacattcctgt

gccagtggattacgtgctgtgacagagaccagatgatgaatatattttta

aacttggaagccatgaaccacttattatttctgaattaggaatcaaaaca

tatgaataatctcaacaggggatcagcagatgcagtctgtccttggggtc

actttgctctggggagatgctcggggtgatggggaaccaagtccagccca

tccttcttgtgacgtgtaggacacggtgtggtgttagctcaattaaccag

gctctgtgcgcgtgatgcaaggttgtactacaaccgtgacatcttggggt

cagagcaacacttggaaacaaggagccatctttatttctgtaagttgttc

gtgtcaccaggtatcttgagaagaatataggatctggtggctgtggtgac

tgaaatggctgtggcttgaaatggtgtttttagatgtatcacacacatct

cttacccggtctttccttttgcctccctctgcacctctacccccatgccc

ctcccaaacaagatctagaacggaggtattctggaattccacccttgtta

aactctaggatcaagggcagttatttaggaaggagatggaggaagaagaa

aacttcccaggtaaccctttggccctccctgtagctgacagagccttcac

cgttcaggggcagacagaccagcgcctctcctaagaggccttgaaacctg

agcttcaccagccaggagacagctaagccaagcccagctaatgacagtct

ccctgttcatatgatgactgtgcagagagcacctgttccagcctctcgcc

cattcttcataaccaagtacacagtgcttgctacacaaggcaacacatag

gaggctcatcggtggcagaagagccaatgtcggctcggcttgctctctga

agggagagagccaagggtgggtactaactgggtttgttttctttcttctg

ggcaaaccccagactcttcattttctctatctacctgttttccaacaggg

tttcaaaggacaacagtggtagcatccattttgagaagcaatgcagcatg

cttagagagctaatgatcgcctctgcctttctccatcccaacccattttg

tttttatgaaattgtttcagaattacagaagagtttcaggaaaataaaaa

tacagcatgcccgtccccctgcccctggccattccctctgccaatgtggg

tcatggcagtactgagccggtctgctcgttgactcctttcttggtcatag

atagccacaagacagccctttccagtaggagggaagctgtgttgtatgtt

cccaccttccgtgctacggagcttgtgctttctctttggggcagagcctg

ggttagtgggagagccaagggtgtgctgactgtaggggtggtctccatgg

ttcagaattgtcgagttggagaactaggactccagagagaaaaatcacct

acagtggcatctggttttccaactcttgtaacagatgcattgttatttta

caaggggagggtccaagagccctggaaatcctggcctctgtccttcacct

gctaagtacgtgctcctagatgggtggcttggaacaagatacacatggag

actgccctgttgaggatcctgccagctctgaacctctcagtgcagtctag

tgcctttacctaggaatggctacagcttttatctaattacctctgcctga

tctggtcttaaagtgaagtgaatctagtgctccgtattgaaatatcttca

gagggaaccacaaagcctcgttgtggtggtgcaggtcttcaatcccagca

ctgggaggcggaggcaggaaagatccttttgtttgtttgtttatttggtt

ggttgctttttcgagacatgatctctctatgtagttggctgtcctagaac

tcaactctgtagaccaagctgaccttgaactcagaaatctgcctgcctct

gcctcccaagtgctgggattaaagttatgtgccaccactgcctggctagg

atcttttgagttgaagacttgcctgatttgcagagtgagttccagaacag

ccggggctacaagccctgtctttaaaaaatacaaaacaccaaaaatactt

tcctgttgctcccaccactgccgtcagagagcagcagccacagccttgag

ctaactcgtggctgtgggcttcagtaagagccataagaagaactccagta

agctgagacccaaccgggacagcaggtaccttaccaaagcatgtcaagtg

cgtgggggacatgatccgggaagtgctggtaactggtacaccaaggagct

actcaaggtgtccagggacaagcgtgtgctcaggtccatccagaagagga

tgggcacacacatccatgccaagaggaagcagaaggaaataagcaatgct

ttggcagctatgaggacatcgctgctaagaacaactgatggccctgcccc

ctcctcagtaaacatgtatgctgcatggaggtggagggccagggggaggc

aaccacaaaacaggagtttgcgaacctccttctcttggtttccttgtgca

tggattggttccaggaagtgtggtggtaaagggctgagctggcgtgaagt

ggagtgcagtggagtgcagaagccttagggagggagccgagtgtagctgt

ggggtgtggcccggctccatcttcatctgggggtgaggtcaggcacccgg

gatgctgtgatagtggctaggtgagagcctggctgatcggtcagacatga

cagcttagagggacattctgcaagggaggcccgttggtccttaagtacat

ttaggggtccaaggcaggtgactgtggtctgaagacccagtaagcaaaca

aaagagttctccgcctttccagtctcactcctggtagcagcattgtcctt

ccctaagagcatccattcccagtggtttgaggtacagcatcctcaccttg

tctttcccagcaggagaagtctcttaaggccaggaccctgtctttttttc

ctttctcaagtggaagccaaggaaaagtggcccctcagccctgtcttgtg

acccaaggttagcatcctccaggtccccctcctgcagaggactagggctg

ctcccttgcgtcacagagcccggtgccagtggtgcatctccctctcagga

tgaaggcaggggtggtagatgagtgttcagattcctgcatgagctcactg

ctctgtgtagcttctcttacagaagcctggtttgcaccccccccccttca

cagttgctcacaggtgaaaaccaggatgacctgccgagccactggcatca

gaatcccatggggtccatgtgtaaaactctcggcctgcttcgatggactg

taatccaaatccctgggggtggaagcatagctgtattgaataagcttcct

agataattgttacgtgaagttagaggaccactttttgtccctgtttgatg

tctacatactcacacaaacagaaacatatacatacctataagatggttgc

gtttcgactgaagatgtagctcggtagacgagcagttgcctagtgtatgt

aaggcactgatgtttgagccccagcaccaaagaagcaagcatgtgtgcac

gcatgcacacatgcatacaggcacatacacacttttgctatctgggatgc

tttgttgtccaggtgtttgaaggtcagtggaggctgatactggaggctgg

gcttctgactagaaatgccaagagccagaggagggaaaggttggaagagg

gtatcagaataaggtttcaatagggctatttgggtggcagagggctcagg

gatggactcactctgacattgatgaggtctgaagcctccacctatttggc

cagctcacagagctctggctctctcggcaggagttgtgtgtcctttaaca

gtcaagaagtgagactttgtggaaagttacagtgctactgttgtgtctga

gagaacgcggataagcataggtgacttagagtagcgttgcagactgttgt

agtgttcctgtgaggtgagcatgcagtcactcttgattgcccacacgaca

ggctctcgaatcaccacagagaaaccactgtgcgtgcctgtctgagagtt

tctagtttaggttcattgagatggaagagcaaccctcactgagggcagtc

ccatgcatggattttatggtctgagcaaaagggaaaagctgagccgaacc

ccagcgttcatctctctctgcttcctgactgtggatgcagtttggccagc

tacttcatactcctgcggccgcggcttccttgccacgatggactgcaccc

tcagattgggagccagaataaactcttcattaagatgcttttgttaggtc

atttgctgtagcaacgagaatagtttctacagtggggtctgggcctgaga

ggaggaaagtcttccgccctccacccagctactctcccccgcccaccccc

accccttgtgggtgagttcatgaagacctggcagtcagtgggaatggggc

ttcccagctttacctggttttagtcaggtttacagggttttagtctttct

tgctcagttaggtaactgggaaatccttttcttgggatttgagtctcctt

ttgtcattgtctcagcagactgctccggtaagacatgagaaactgtgaga

agtgccttagagagcttaggatctctagatggaggtgatggggaggggca

gaccctgagagataagcgctagcttgtcctgcgccctttcccctcctgac

tgagcatctgggagcacctcttcaggaggacctttcccagtctgaggcag

agggatgggtcacttggcctgtggacagctttaccagtggctgccaccaa

gtcactcctgcactggccaggtaaaaggcgaagggattggaccatggttc

tgcagagcaggcgtcatccatgtgggcacagtggtacaggggggtgactt

gtgtcctgcagcccaggctgggtttaagtaagtgcacatgtgtgcctcat

gcctgcagaggtcagaagagggtcagatcatctagaacaagccatcatgt

gggtgctggaaaatcagccccgatcctctgcaagatcagcaagtgctctt

aaccactgagccatctccctagccccattgcggtatttgtaaacgaagtt

cagactctcagagacaagactgtgttgagtcttgattctggcgatggaga

gcctgggctagtgtgagagtctaggctactttaaaatggatgtgaggtag

tctagttagctcacctttctctgaagtgcccatgtgcctgtaagctgctc

acctttccatgtgccatgccattagcagtttgtagatttctcttcctggg

gcactggagaagcctggggtagaggtagggagcagactgaaaatggggaa

caaatagtgtctacagactttacagagtcaagaagtggcagtaggaagct

ggcctcagtgtgggagcaggtcaggtctctgcaggtatttagatgtctgg

aaccttggtgacataggacatgccttgattgtagctgtggtctagatggg

cagtgctcagggacccgggcccactcagtccccagtatggtttgtccttt

acttcctgtagctgttttcctttcattttcttcatttggaaaataatcag

caaaatccttatttccataagcatttttttcctggctgtaaaaagctgaa

tcctaaacccattattatagctgttcatgctattttaatctactctgttt

agaggtgagagggtagcagcgaatctacaattactgcgtacatttgatcc

atggcacagccaccggagccgggggagaaccctcccgagtagggtggtac

cacgtgtggcaaatcactttcaaatttactaaaaatatgcccctctgcag

ccatgtagatatcataattttcctccccaagtcttcttgaaatctccgtt

tgcttcccacccagcgctggctcatctgcctgctcgctttcattcttttc

cttggccttcgtgcccacacactgccccccatgccgtgtactgtccgtgc

ccccctcctcattttcatctcctgctcagaaatgtttcctatcctactac

ccagcacagtagtggaaaccatttttggtttgccgcggtcaagaaggaga

atacttagaattttaaatctcctagctatttcacatttaaacaagctggc

gagctggagtggtgccgtgcctcctacatacagtaattgtcacatcccta

ggctccccctttctatctctgttcctacttcattcatttttctatttgca

gccagtgcagaatatgggccgctccccctcgcttgctcctgcatcccgct

ttccatccagtgaccttggcttccttgagtgccgcactgtaaatgaaatg

tggcattgccacatggatcttgataaaatatatcagtgcatgaattgatt

ttcatattgatttccttgggccagcaaggtgtgtgtctgtggaagccaag

cgtcttgtaatgaaggtcctagaaagtggtccagatgccgagggaggggc

gggctttgctttttttttcacctaggaaaactttgtgcttgttagctcag

gagagagactcttgtgctctctgggttcactctttgagcatatagcctca

ccgaagagagagaaacattcatctctctgctctgtggcctgggctgtgcg

gggctatgatctcagtgtgtccagctgctttgttgctagagagacctagg

aaaccatcagggtagctactgaaggccatgtgcacccagcagtccggagg

gtctgggttggggaggtgtgtgtgcatccccccccccccccggtcatttc

ttctttcctttctcagccaagttgagatgtggcacttgtgaatttaggac

tgatgctctgccctaaagggatgtatataattcatgatgtgtcaacactg

atgtctgagtgtgcattggattgtgctcttgtagctccaagcacacacac

aggcctgtgtgtggtggagtctacaggatatgactgtgacatggtctcct

gccttacagtgacgtttatgagtaagctacggacaaccgcaagaacagcg

tcaagacatgattaaagctttcctttttccctcctctgtcaccttcttcc

cagtgccttctctcaactacttctgtgtagacgatgccctcagcaatagt

tcagctgatagttgataaaattaagtgagcatagcttcacgtgatcccat

gtgcctgtctgtgcttatgtagggagagtaaggcagttggaattgctgtg

gtcactcattgtgttttctagataactagaaagacccaactagaaaacct

ttctagttgtgagctgtgctgagatacactggcagcacggcatagtgtat

ctatctcctgctctggtaaatgccaggaggagaagtcattgagtgctttg

gtgtcccatgatctcattgcgctcgggaactagtatcttggttcccagtt

ataatggcatcggaaagcatagagctggcttgggttaatatagtcagtag

ttttcagccagggacaaattcatgctcacgggtgcatttagtaaaatccg

gagacagttttggttgccacaatgtatgtaggagagttgctgctggtata

tactgggcagagctgggaatattcctcaccttggtttctggtttctgcat

gcctttccctcacctgctataaacacatacttgctctaaaatggcagccg

tgctgggttggaacatccttgccccaagacagggctttgagtatattttg

ttgttgtttttgctgaaaagtttgtattgaatcttaaatgtaaaaatcac

ttcccccatgggcctagattttcttgagtcaggtgttggaaatgatctga

cataatggaggtttctcattcactggcagttattgttccagagaacaagc

atggaagcctgccagcatgcagatcagcaggactgtccccagatggccca

cccagtgaatgcccaaattcattgattcatgtatggttagtcaaagccac

aaggatcacatgagcttaaagttggactttgagagcccatggtctcacaa

ggggacatggttcacccttgttttctttgcaactgaaactacattttcag

aaacttctgatagaactttcctgagatgcctgccaggcccacgggactga

ctgcgcatcagaggcagtttggccgtgggtgtcaaacatcttcaatgtat

gtagacctgggcgttgcttcttggctggcccaaggtgagtccttggcaga

atgctttggtcttggtgagacaggtgtgtgtgtgtgtgtcagccaaggca

gatattttgtgtgctttgattgttgaacaacattattggaagccaatgac

tgtcatatgatcagcttatttaggcagaatctcatcaggaagggcagggg

ttagtgaataggaaggacctcctttgttaaagtaacgggaaagctaatcc

caaacgaagctcccgcgttgaatgttctagaactatcgtgtattggtttc

accttcacctctgccctctctcatgctggaaaagagaagttaaatggaat

tggtgtgtttgtctgtgcttctggcaaacacaggtcagatctttaagaga

tggaatgccctggttttgattgggtgtgccagttgcctgtcatggcgacc

tctgacagggcccccattgtttcatacacactgaaaagggaaagaaaatg

ctccctccctccccccgccctggaagatcagttttccaaacctttttgaa

agggcagtgccacacttccttcggaagtaagttcatcttccatgctatat

tttctcatcttgcctttgctgtctgccagcagatggaagggggagagagg

gcttgttggtggactctgggtgagactctggagtgactctgcagtcttct

ggctgtgcaactttgtgcaaatttctaacttgcgctgagccttataggaa

ccatagcatactctcttgagcctctgcatgtttgctttcttactttccca

gaactgcgcatgtttcaggcgctctggattgttatgtggggttttgcgta

catcctaagcattgttcctcaaagacgtaagagcttcttttccttctctg

gggtcacctgacttgttgttaggtagcttaatcgaactggaggcgtacct

ggactctcccttggtactctgtagactcttgggtggatgcatctcctgtc

ctgtctcttgatagaagagtatttatagctgggtacaagtacagtcagaa

gattcttataaaacgctgttgttactgcagcaccaagttagaaatgcttg

aggtttcggcttcttggctcttccatgcggccatgaatattttatgggaa

aggaaatacttagtgtctgactctttttatagtgcagctactcacgggct

gggaaccttccagatcttgccaaaatcaaaaatcactgcccgtccatcca

tccctctactttcttttcagagcccaccagagccccaccgtcactgaggt

gtcgagctctcttggtgaatgaccccatttgtaaaactcgagaggtgcag

tttccagtcctaataactatcctaagctggaggggaggggtgagttacca

ctcagttctacccagtccttctggcctggggttgctggttagtacctggt

tggcatccacatggaacataacacagactgcttctaggtgcccatcagca

ctttcagccagagacttctctgctcagggagaagtgtttcggaatcccta

gagtccactttgatcttggaacataaagatcacagttaatctggatcagg

tgctatccctctacagtcttagcaattgggaggctgaagcaagaagatct

taagtttcagacaatctcaagaaacaaaacaaaacaaaacacagcatcta

cagcagtagattaaagtctggcaggcctcaagaggcttctcctttcacag

gtaaaggtgggtgagatacctttcccaggacttctttctcttcccttcag

tgacccacgtgtaatgcaggaaagtgaggcctttgtggtttcagctgtag

ctagcttggtggggactcggtgaagaggaagaaaggccttctggcctctg

aaacatggtgggagttcttggttggcatcagtggagccctccaggagtac

gtgctcatctggcttggatgcacagtctgtagatgcctgctatgactaca

gttaccttagacctggtgacaggtcagatcacagtgtctaagagcactct

tactttgggggaactagcccagtgtgacttctcagagggggacagagacg

caactgtctgtctctcgcaggtgagctaagtggcctatatttttaccaga

ctccccagactcccaagcatcaagcatggcttctgatatactcaggggta

caactggtttctcgtgtgccagatgaagacatacatctcttaaagatgta

tttcatttttgattatgtgttggagccccccctggagctacagctacagg

tggtttgagctgcctgtcgtggatgctgggaactgtattccagcctgcta

gatgagcattatacacttataatcacagagccatcccttcagcttcccag

gacagattctcttaccaagagcaagtcggaaataagtgtttgatatagtc

tgaatctcagctttacccttgcctgctgggctctgggagctcaggcctgt

tctacatctgcctcctggactgcccttggcgttctccttggcttttgatc

gggtattgttttgcataccctttgtggttacgtcactatgaagcagagct

gtctctccttggggaagctctgtaccttccccaccccttgtcttggtctt

cagactcaacagtagcaaaaggcatggtagggaatagaaggttctggcat

ccttgctcctgggctcttgtgaggaagagccttccagatggaagtgcctt

gggaaagaaaaggaagtgaaaagagatgaagaggctacccaaggctctct

gcacatctcaccctccctgggcaggaacttggtggatgtattggttgggt

acacagaagtgatacaaagaggatttcgagatgacaattacgtatttata

tgatgagacgggaggattttcaggcttctaaatccttgcagcttgcttgt

ggagagaggttggaggcttccaacaaggccagtgcctttgagttagactg

catgtgtctcttgggagggagatacacatgcctgctcactctgttacttt

atttcagcgagcttggctgtgtccctctccatggtcagagcagggtgggt

tctgtggaacagacagtaagcaatggcattggaggacagtgttgtgagtc

ttgctgaggtttccctcctggcctcagagaacaggccagaatcgggggtg

aagcactgcgtggcagtctgagcacatcatcttgttctggtgatgaagta

tagaccgtgttcttctctgcactggtgaggaccacttaggcaaacacagt

agttcttcatgaagtgggctcatgaagaaggtagcaagcaaggtatgctg

tgttgagtacaaaatgaggaacctgaagggtccaggctaggaggatgcac

ggcagctgttggctggcgtcactctttatgcctgtgcccctaagttggtg

gtgtggggaaggcaggaggtggctcttagggccaggtatggattataact

gtgggggtgggggacagactgtgtgactcaggggtgggtagcatttgcta

tttcagtcttggtagactttagaagtttgcagcagaggccaaggaaggat

gtaagaactgaaagaaggagggatagaggtttggaggtgggtgtgacccc

agatggcaataatcttacatatagaaaacaggctattttttgcataggtt

gcttggagcttccacgcaaagctgctggtattcttcctgcagccttaagc

ctcggaactccctcttcttgtcacctcacctctcccttgttcctgttggg

tatataatgtcttgtgttccctacttgattatagactcttggcagacata

gcccagtatgacatgttcctttgaaagtccttcactgcgattggccccat

gccttgcgttattagacggcttgaatggccagaaagaccactgctcattc

ctttcttctcaggggagcggctcatgtatactcccagttttcttgcctgt

ctcaggccagtttcagtctcaaaaagaggcagaggcccgcctcttcctct

tctcagagtgattctgtaaggatttataacatgtgcatcaagaaaccgtg

cgtggtgagcgtgtagagatggagcagttgctgctacgtggaagatcata

ctccaacaccagtggagaaacttggaggcctacacttaaaaaagggaaag

aaaagtggtgtgtgtgtgtgtgtgtgtgtgtgtgtgtgtgtgtgtgtgtg

tgtgtgtacaggcatgtttgttctcacacacatatgcaggtacctgatga

agccagaaggggacgtcagagtcctctggaactggagttaaagatcgttg

cgagccacctgatgcgggtgttgggaatgaaccccagacctctagaagaa

cagtaagcactcttagccattgacctgtctctccagcctgccggctgcca

cttttgattcatagtcttttgctactgactggtcaaggtaactctccagt

tctaaagaagaaacagggccagcatccaattccctggcgtttcttggttt

cctctttcctactggttattgttactgtacttcctgcctgccggctcctt

tgtgacggtggatagtttgtccaggtgcctgtttgggttttctttggaga

tgggaaaacctcagtttactataaaacttcctaacgcttctctaaatgga

acgtgcttgcttcgtgggcccctccacgtttcaaatattaaacagctatc

tcctggagacttctcctcccctccccccaccccacccctcagaaagctca

accagatggtgagaggtggtgtgtggaggtgggtgggggctgctgacttg

accacatgtgtgtggtaagggttctagggaggatacccatttctgtaata

tatgtatgtgtgtgtgtgtgtgtgtgtgtgtgtgtgtgtgtgtgtgtgtg

tacatatatatatatatatgtatgtatgtataaaacttactaggttccag

ctacagaacaagactgagagtgttggcaagtggccaagcacagtaactgg

caggaagggcctgtcacttcaatattctcgaaagaaatccagttttccat

gaatgggaggctgtggaggaggttggagaagcaaatgcctggcttgtctc

caaggccagatgttctgtagtgccatatgtcatgacgggaaggctagcat

tggagcttacagagcacgtcaggcccatgctcttaggctaccacagccct

tctcgagcttgctcttcctgctttcctggcccgcctctgtggtcggaacc

cttccgagccactcagatctgctgcagttggcgagtgtccagagtgctga

ccttagccgggactgattgctatgctgctgggcagtgcctgctggatcct

tgatatctgctcagcccagtcttgagaccacttcaaaggaggcctttgcc

aattttatttttcttcctcagcctgggttttgtggtcattaagatgcggc

atccgaattgcgagactcttgttttcaacggtgtcttcacttcttctctc

agtgtctgttttcccctctttctttccatcttccctccctctctccctcc

ctctctccctcccttcctcccttctgtaggtggtgtgtgtgtgggagtgt

aggtggaggcccaaagttggcatcagaagtcttcctcagtcactctttac

ctcatattttgaggcagagtctcacactcttcagctagtggatgaccagc

gagcctccgaaacctcctgtcttcagactttgtaggttctggagagaagg

gacgccgagtgatcttgcttcccaccatattgaattggtagcacagtcac

ctgctaaagggacaaagtaacatgtcatggcccttctactgcacactcta

gctgacatctctgctgtgtggtggtggtggcatgctgaactgaataacag

agcaacaacaggataacagacagaagcaaaggctcttttgggctctttca

ggttttctaccatggtcgtttataatccgtggagccaaggacctttagaa

atagaagcatcttctttcaccatcttaggagcatcttgtgttgccatttt

gttctgcaaggctgacctaatgactatctttggaaatatcgtcattctca

agttagaatttgggtacttcattcctttctccgcttccgtggtccctgcc

cacaccaaaccttctgctcccagctaactccgcggttccaagaaaggttt

tggtggctcttcagtggcgtagcagtgtgagcaaagtccccatcctcttt

gccaggatcctgcctaagccagcctaggttaccacttctgttcctccttt

tccgtgagaccccttgcgatgagtcagactagtctcctgagtacttggct

accatggctcaagtcttagacatacaggggggtttccattcttggcacta

cctgggtagagagagctattccgtgtagaaaccagctctgctctgctctc

ttcagtgccatttcaacttcagcacccaccctttctctgttcctcttttg

tacccccataacatgttggacatatatggatttttttttttggcatattg

acacatcaacacttcctcctctttggatcccctctgtcattttctctacc

gtcagtatcttttgtccaggtctgtgtcttggggctacattactggtcca

caatactgggacttcaggctgatcagggagcaagggtgtctcttcctgtc

cttcagcccacggttctcatctctgtgcttgctcacatgcggtccttcct

caacagcctagtgcagtactgctgctgtttctgccccttctcctaggtga

acaaactgagactcccagtctaccactgtagacctggggtctgtgtgtag

ggcgttgtagcaccagaatctggcttcttagcttattggactgccttaat

ggtaccatgctctctctcctgatcatctgcactgctgtgggcaagcagag

ttttttgttttgtttttgtttggttgttgttgtgtttcagttttctcctc

agcaaaatagaaataaaaataattggtgcttcatacgattgtctgaggac

tgagtgttcagagtagggcccttgtaagagcttagctgtgtgtcatacaa

gcatctattcgttcttccttactgacatcatctaaatattattatttgta

atttttttttaaaatcacctcttgtgtgtctctggcctctttgtgatgct

gtggctaggacaatccaggtctctgcacatggtaggcaagtcctcttttg

acagagccccaccagaggtgcctgggagacaaactcgattgtgatgtctt

tgtagcagatgttgatctcaggcttgactttggcagtgtccggcctcagc

aggctcagcagtgtgtggcagagggtagctgggggtaaaacatggcagtg

ctgggtagaggctgcagtcctcagccctcctgttgctggcaaacttttcc

cttcccacccccttcccggtagctctgtgccctgccaagcatgtgcacaa

gccttgaaatgacaaagtaccctttagttaattgcacaacggattgccta

gagttaaaagttctgatttatatgactatagctctaactgctcaataaca

caggcctgtaatctactttcatttcaaacagaacggtgatttatagggca

gcatgccgctttagctcagaaatgaggtgttcaacatatgctctgttgac

ataaatttggaatttatcgctgttgtcaaaactgcctgttggacaaaggt

gggtattaaggcaataaatggctcagaatagttttctgtcacatttccta

accattgtattcagtctatttgagggaaaaatatatacttaaaagtaatc

aaatgtccaaaatccatgcatcttgctttgaatggagaggagagggagga

tgcaggggtggtgggggtggcggggacgtagagacaccgggagggggagg

ggcagatgtgggcggtggtgaagggaaagcggtttgtgaagttatggtcc

atttaagagatttcactttgctccctctcccatccccattaggaggtagg

ctttcctttctcatttacaaaaaaggggaaagaaaaatgaaagctcatta

aataaaaatagtaacggacatttttatgggtggacttaaagccagcaatg

tcagtgaggagttagtgagcggggcttggagggaggcaggcagggatttt

gtttatggggaaatgaaggcaccagctggggttgggttctctgctaggtt

ggtgagtgttagccttactcccaaatgcccccttcaagcttgaggttcct

gtggactgggtgaatatttaattggcgactgatagcagttaaagagctgg

tgggcattgtaccccacccaggtcctgagtgatttattatccactgtgtg

atcaatacccaccaatcactcagccatcccctcaacaatgtgtcttctca

tagctattattcctatgccctttgttggacttgagaattcagatgaataa

ggcaaagctgtcttctggttgcccatctagaaggtgagacagagaagaaa

gcaagtggagactcaccagtgcctctcgtgcaaccgtagacgcgcaagcc

aaggcaagggatgtggttctaggaggggaggcactaggaaaggtgataca

agctcatgtggccctttcagagcacgaagtccctcctgagagaacatgag

gtcttgagaattatcctaccatggtaggacttcatttgtttgtttgttta

ttgagacagggtctcactgtatatcgctgactggcctggaacttaatgtg

aaaacaacgctggctttgaactcacagaatgccacctacctgtacctctg

gagtgcttggattaaagtcatgtgccaccacacccagaggctctccactt

tgttaatgtatgacatactgttggcatacaggtgactactgacatatgat

cactgaatttttttgtattgtgtcataataatatattaacaacaacaaac

attattgttgttgttgttgttgttgttattgcagtgctatgaattgaacc

tagggccttgttctgctactgagccacgtcctcagatctgtcatatgttg

taaaaagcagagtcctgaagcctgtcaccacagcatctcagcacagacta

ccagggcaggcagtaggcagaacagggtgacttagtgaatggtgtcagtc

aaggttaaaaatcaatctccctttttcacccctagtttcaagataagaac

ctgcagtcacttgcgggctggtgtgcaccatgctgggaacgaaagcaggc

agatccagagagctggagcagggctctgtggcttgggccgcagctgctcc

tgggggcccgcgctctgtctgggtattatgcatggcccatgagcttgtca

ggaggcagaggggcatgcgtgctgcctggtacttctcatgaaaacgaggc

ccagtggggaagaggatgcatatttgttggctttattttatttttccttt

tatctctgcccacatgcttggaccgtcttaacgttgataaatgtgcgcat

tctcgctcattctctctctctctctctctctctctctctctctctctctc

tctctctctctctctctctctcacacacacacacacacacacacacacac

gcacgtcttcattctctggctcgtgtgtgtgcctcacatattatacagtc

ttgtgcaccctgaatatggacaccaattgtacccccccccaccgtgtggc

tcaccccaccaccacccctttgccaccaactcctgtccttttcacacaaa

gctccccgttgggaaaactcataatttttacaccacacagtcttgactgg

ccattgcaggaattactggctctttgaagtgctttcggttggggccttta

caggctgttttaattacctgcagcttgcaaagggccttgctcggtccggc

ctttgtttgtttgttctttgcaagtccgtgtagaaagggaacctgctgcc

ttcttccacacagagaagggaacaagaagtttaggacccgtgcacagagc

ttctctgagcttcctgcttcagggtgctctgcctcctcagtcaagctcag

acagagagggcaaactttacttgtggcttgcccagctgccaactctgtgg

gcccctctcttagaatctgccaggtattttgtttgtttcattttgagcta

cctgtgcatgtgcccaggccatgatgcagggcctgggtgcgggggaggca

gggcctgcttggaggagttccaccctcagcaccaaacactttcattttca

agcatttctttcaaggtctaccttcagacctcccagcggagacaacaggg

ctgtgaaatcacacagcccactgttgaccaaatcaaaattggtcacactt

gagccaggatccctttcgatttttcagctaaatctgtttagggaatggtg

tcattcgattattgagtctggacttcagatgcaggaagggggttgacata

atatgtgtgagagtggtcatcattgtgggctagctggacttttcttccct

tgtcttcgtttgcttttctatttgatgcctttaaaaaggactctttcagg

ggctggagagatggttcaactgtgaagagatcttgttgatcttgcacagg

ccccaggttcagttcccagcacccacacggcaactcccgaatattcataa

ccccagtttcaggagatccaaccacccttttgtgacctctgtgggcacca

ggcacacacatcatgcgcatacataaatgtaggcaaatgctcatatgcat

aaataaaaatattaaaaatctttataaaaaaattacagcgactctttgct

gttcctgttgagcctgagtgtagcctgtcctctgtgcaggtaatgaacta

gaacgccaggggagcatggggcttcgtcccttgaccactgcctacatggc

agaaggactctttattcatttagtttaaagccatgttgtgctggtggaag

ctgtgagcatggctgaaggatatgtagactggttcctcagtgtgctgagc

atgggtgatgcattatgacgactgccagttccgtctctaggtatgtaccc

aacagacgtgaaaatggtctgtccatcacaggttcaaacttgcatgctga

tgctcatagcagccgtgtttgtgacatccacaaggttgaaatggccgcct

gtccatcatctggtgactgtatatacagtgtgtggtacagacatacaagg

acatgtattccagtgtacagaggaaggaggtagcctgtgtaggtctacta

tggagaaaacttgaacaagtgtgttctccgagagaagtgagatctaaagg

ggcccatcttatatgatgtccccatctgtgacatgttcaagatagccgaa

tgcacagaggaaggtaggttagctagaggctgggcttaaacagaagagag

aagggcagccattgttagtgtagggaaaatattctagaatcagatagcag

tgacgggtgtacacttctgtgaatgtactaaaagcaaaccctctcaacag

tactcattaaaaaggtaaattgtagggtgtgtggataatatctcaataaa

gttctgtttagaaatacccactgactgtgtcccaggcttcctgccaggtt

ctgacagcgcgctggatagcttctgccttgacaaagcttgcagtctaggt

gagataaggaatagcaaatgcccattgggcacctgcagggaatgttagac

cctggaaaggagactgagaaagggcagttacagagcactgttggtataga

agctaccccggcaggctgtggctctggcacagttcggcttatgtgggagt

tggcacacaggtgcgtagcagactcagcatgggcattggtaaggaaagag

tcaacgtgtcctccctgaggtgcatcctgaaacctgtgtgaggtgatcct

ggaagtggagtgtgggggccattggtccacctctttccttgggacttctc

tgtgctgctctgtagctcaggttgggaagtctagtccagtgtatcttttc

tggctggtggttttcagaaagccccttcaatttgaagtaaggtacacgct

ccctttgggactaaataacctgagatgacctgattgtgtatttctccccc

tcctcctcctcttcctctccttcctcctccttcccctctcctccctctgg

gtgtatgtatatatgtgtctttcttaatccctccctcctctcttcctctt

tcttcctccctcccccttcctttcttctctttccttctctttctttctcc

ttccttccttccttcctcccttctctgtctgtctgtctgtctgtctctgt

ctctgtctctgtctctgtctctctctctgtctctctctctttctttcttt

ctttctttctttctttctttctttctttctttctttctttctctctctct

ctctctctctctctctctctctctctctctctctttctttctttctttct

ttctttctttcttccgagtcttactgtgagcagccctggctgacctatat

ctctctatgtagaccaggctagcctcaaagtcaagagatcctctgcctct

ggagttctaggattaaaggttttattattattttttccctcattggtcta

gaagaaagtggtgggatttttttttagggggggtaggttttgttttgttt

tgttttaaaataacaatctgtctcaaaactgacaaatctcaatctctatt

ttcaaactgaaaaaaaaagtaaaaaaaaaacctctgtattcccccttcat

taaaatgacaataatttccccaccgtgatttgagtgtgatttgatgtcca

tagagataatctgcccagctcattgctggtgttcataactgatgtaagag

agcctggctttctagctcagaatcctcgtagaaaagtaatttaattttca

gtgtagtaagctggaagaatgaggcaatatacattaaaagggttttcaga

gtggtttaattccgaaggcatgtgcttgcgggggctttaagagacccccg

ttggtctctacagtggtccgagtttgcagatgggtccctaggatttacat

cagcatcctggtcccctggctgaagtggaaacccaagaaacaattcctga

taaccaagaggcaaagacatcatctcccatactctaacactgggagatcc

ccaaagtgtctgcagagtgttcaaagctgttttcatagaaactggctatt

tgtgctagagaaaaaccttgtatgggagacagagagaaagattaaaaaaa

aatcaggccctgctcctccataactgtagcccgattcccttatagggccc

cgagggactggagagagctttggatttattcttgtttcccctgggctgac

accatttagcatgcaccacagtaccctgttttgggtttggaagaagctca

ggcacgctataaatcttgaagctttttactatttttcctctcctctcatt

ttctttttcttttctttctttctttttttttaaacacgcacacaactttt

cttctttgttaaaagaaaaactacaaacgcgtgtattgattcctgccttc

agatcttaactgccttggctgtaataccttgcttacatctgccaggtttt

tgtgcagtgttgcaacatctccttaatccagtgagcataaatagaacatt

tcagtcatatttggctaccagcaactccacagataagcatgctataagac

cttgttgaaagagactgtatgtgattgtgtgtgataccatcagcccacag

cccagagcccaaatgaaaaacttttgttcttggtgaaatttgttgataga

gatatttcaaataaatgacttgggagaactcagtattttaaaacaagggt

taggttatgtaacatccaggagcccaggccacccaatgctcagaggccct

gagggtccatcttagaaccctgtctcagggagatagatcaccgagttaca

ttgtcacttcctatcctgccctcccctctctttttcttcagttttctttt

ctcgagaaagctctcattaaatacagtcttgactgtatttaatcaggttg

tccttgagctcacagagatccacctgcctctatctcccaaatactgagat

taaaggcaagtgctaccaccacctgtcttccgtggtggtagcacttcctt

aaaacagggtctcactgaatccagagtccatcaactgcttggatcagcta

gcccgtcatctctagggatccgcccatcttctccatcttcccagtggagg

ctcacagatatacaagaccatacccctctttcatgtaggagctgaaggtc

tgatctgagaggtccttgtgctgcaccttacccatagaaccatctctcca

ggcctcagatgcttttcaaagcataggactatcccatcccctcatcagtt

ggccaggtaggggtcagtgatgtatgaatggctttcatgctgagagccgg

taatagggatggaggagagatttctctctcgggcaatgggtacagttgta

ctgaagtgggtgggcatgtgtatagagggaaggcaggcttctccatctcc

cacatttcctcctcaaattccctttgaggtttgtttccgtcattttcata

aggtgcattgtgcatttcatgaaaccttttgcatgtgctgtggacccctc

atgagagggatatggtacagccagtgtagcctgcgtcagccccaccacct

tgcagcttgtgaagctgactatcaagaatctatgagatcatcttgatcta

gtcgtgcctgcctgcaaagatgcaaagggctggagagacaagagatttga

ccgaggatgtctgctatatcctaggatagctggaatgaaaaccactgtcc

tgctcccagcccagtagacacataagcctggtcaatacatagcttctatt

gagatgattcttgagaaagcatggctccttggtattccttctccttccct

tacagagactccaaatcaaccagaatggccccttgactctcatcttctgt

gagcctgcatccctagcaaacctgcagcagggaacgtgttaaccaatact

gttcgttctcattgtttttaaaatcgcttctttgaaatccttggctttta

ttagtatatttactttatggcatacataaagatctgtattaaaaaataaa

ttgtgttgtaaaagcattgggtgatgtttattaatattgactagtcaaag

agccatggactatattgctagaaggataatttgtagtaataccagctatg

catgtgcagcttgtcaaatgactgtctaatctccacatttttaaaaagaa

gcggttgaaaagacaatttcccttctgtcttgagaaaatgaaaggagact

catgcctagctgagtctggaactgtccttgcccttcccctgtggctcaag

caggctttcaggcaccaggcaggtacacgggactacgtggcagtggccag

gtgtgtccctttgtcttagagcagttagagaccacttcactagaagttct

gtgtctctgtctgcttccgtctgcgttaaaggcagagatggaaccatgat

tcctgtccttaagcttggagttctgaggaacatggaaacagcttagtctt

gagtgatgtatagcacaatgacacaaggagtgactaatgacccctcttgg

aatatgacacggctgctgacattaagaaacattctctgggaagatctaat

gtcacctttagaaggtagggaacattcatcaagtgtcctcgggccatctg

aagaacttgctttggtttagggtggaggtctgcggtagcactcatcgttc

aaaggttttctttcccatgaagctccatgcagggggctgagcatgttagt

acatgttgtgatcctggcattcgagaggccgaggcagggggatcatgagc

ctggagccagcctgggctacagagtgagttcaaggtgagtcagggttgta

aatcttgtgtcaaaaacaaaaaaacaaaacaaaacaaaccaaggaagtgg

acagacaccaaacctgcagaaaatgtaggaggctgggctgggtagtatac

atagcctccatttccccggaagttggctttgtagaggaactctccagtgg

gctcatctttgtgccagcacttcctcagaataccgttgactggttgacct

ttgacctttgacctgtgaaataagggtctgtgagcctcagacttaagact

ttaaaaaaaaaaaaaccaaaccacaacagtcttgagaaaggtgagaaagg

ccaagattgaagtctcttccttctctcagtttagctgctgtttagaaagg

tgaccgagcccagctagaaggttttgcagctgtgctgagagtctgtttct

tttggtgctggaagctttggcctagttcatgatgtccccacagggagaga

ttggctgggagtcagtgtttgttgacagagaggcccaggcgtttcgcttg

gggttagttagcttcgcctgtgagtagaagaccttctgagagggttgctg

cttgcatgagcttttgagagtgcatttagaccttggtctgggtgtcattc

acatgaagtcttatttccttcttgaggctgccacagaatcaccaaatggg

tcaaatcctgtcaccttgacatatccagaatcccctgagataaaaaggct

caaggagagattgtctccattgggttggctggtgtgcgtgtctgattggc

tgtcttcgtttatgtaaggatgagcttgtctatgtgtgagtggtgccatt

ccctaggcaggggatcctgagctacctaagggtagagaaatggagctgag

caaaggcaggcaggcaggtgagcctgcagggttcatttctccctgctgcc

aactgtggatgtgcctggctgcttccagctcctcacgctgtgacttctct

gtgacggtggacttgaaccaggaattgtaagcagaaataaactatgttca

ccccctagtaggcttcttttgccacagcatgttagcatagtaacaaaaat

gaaactagtacatctggtcgtgaattagaaggcaagtgccctagctgggg

tttgggttgcagacctgtcaccctgtcccctggaggctgagaccctagga

ggacagcaggagttcaaagcagcctggtgacctagggagatcgtgtgtca

caatgaaaaaaaaacgaaagggggagcgtgctcaaggtttttagtcccca

gtaccaccaaaacaaacaaacaagaacaaaattgccagtgggactgccat

aaacagtttagcctctgaccaggccctgagtgaattgaaagaatcttttg

ggccttattggcctggtctgagcattagttcagaagatatatcaattaac

caagaatgggtcaaacattgtccagtgtctggctgggcattctctgccag

tgacttgtcctagtttccaggttccctgttctgatctcttctccccaaaa

ctcacagatcacactatttcagcttcctctcagcagagtgagggagtaca

gcccttgggctactgaaacctcgagcttttcccttcttgctagctagtgg

ataagccccatctttgccctttatccctatacaaaacacgagaagaaatg

tggtgtggccagtaccaggccttgagctccttcatcccccaatccacaag

ccaaagcctctggtcgttttcttttggcaatgccctgaagcccctcctca

tcttgtctccttggtcctttgctgagtgggtggaaatgtacaacttctgc

agagggcatgcattcagggctctcttaaggttagggccagctgtcctggg

gattgaggtagagaagacatgaagagtcactccttgataacatgcatgtt

cttgtgttgtccattttgaactgggttgatggaggcctattctggatggg

tgaggttggtgtgaggctcaacatttatctttccagaaaattaccatagg

ccttccttgtatggatgtcaagagacacaagataaggtctggttctggaa

tggaggttgcctgtgtagctgttgtattcagattctttctaagataaaga

gaatatagacctatacctgcttttcctagaggggtttctttggctttgaa

cagtccctttaaaatgggtcttagaaagcaaaggcaagatagacatagca

tctgtcattgagcagttagtggtccaaaccagacagaatctccatgcctt

gcagactcattgagcacagacatggtcccagtgacaacctggccagagtg

gttttataagtgagagctacgttggagagaacaatgagagaaaatgagcc

cttctcttgtgttactaaaaggttcagatttgtgtagatgtaattttctc

acagtgattctctttcttgaggaaataggtagatgtgttcaggaattaac

ttttttatcttcttgagtataagtgaacttctgttccctgcacagtgagt

tacttttcggtcacatgttgtcggagatagatggaaccactctattacct

gatgactttcaaaatggcggccatcagtctggcttactcctggagaatga

acacgtgcctccatcatcaagggaggcagtgcaaagtaagaaattaagcc

ttgtgaagacaggacaggattgggtagatgtgttgaacatagccaggaga

aaggtaaggttggcattttggtcaaaaatctctgcagaatgtggcgacag

tgggtaaagagaggtggcctaggacaaaggttagactctgaagactgttc

cggagtcggctggttgctgggtctgtagaatctactcatttcttctctgc

cacttaggctttcttggaattgagagaagcctccatgttagatcttcttc

cttgctgtgtaaacaataacttttttatttttttaaatgagcaaacttaa

tatagggcaccattgacgttccaggccggcctgtgaagttgcccccctct

ttctgtcttgaactttgtatgctttgcgtggttctccctgtcaacggtga

cctttgtcctggctacatgtgatctcccacggatgccatgctgggtctct

gggaacctgtgtggggcactgagttagtaggtcagtttcaggacagcgtg

ctacccccgagaattgggtaagagagaatacacgtgttactgaagaaacg

gtgactgaaacagttcagaggaaaggctcctgatgacttctagaaggttc

atttgctaagagaaagacaagataaaggcgctctggattagaaggtttta

acgtgcacagattgctgggttgggtttagagttttgtttgctatatcata

gcctatgacctgggtgtcacagtacattacaataggcagcgacacgaaga

tcgtggcccaggaaaataactgtccctttcaggaattccttggaggcaaa

gtcaaataggatatgactgagacttttgaataattaaacttatctccgcc

tacaattttagttactaagctatgtcattttacaagtgagaaagaatttg

atagctgggtgtggtagtgcacactttggatcccagcacccgagaagcag

aggcaggcagatcttttgagatctcaaggccagcctggtctacagatcga

gtttcaggtccatcagcccttcataaagaccctctgtctcaaaaaagtaa

aatataattcaagtgggtgtgacaatatgacttgtccaaggtctttaaac

cagccagggctgggtcagggtgagagccggttttacatctgtgtgcttgg

ccttcctccttgtgccctgcaaaacgtttacaggaaacaggctggaatcc

atcccctttcacacagagccagtctggttggcaactggaagaaaagccac

tttcagcaattgctgagcaaatcttcccatatccggggaaaaggcaaggc

tgttacttaagagtgttgatcggggtgagagatttgatctggaactctgg

aggaggaatcgagtgtaggctgttgggggtgctccccagatctgaagact

gaccatccacaatcctttggaaagacaaatgcatatgggatcctcctatg

cctcccaggcagggacatccctggaagctaagaaatcataaatgattaaa

aggtaacaatcagttggacccaactcctaatcagctttcaggaaagcacc

tgggaaaccactcttgaagagtctttgtgttgtgtagatcccttagaaag

gcaaatatttatcatgcccaggcaagggacaggagtgccccactgtgccc

cacccctgtgtgtgtgtgtgtttgtgtgtgtgtgtgtgtgtgtgtgtgtg

tgtgtgtgtgtgtgtgtgtgagagagagagagagagagagagagagagag

agagagagatgcctgggtacctatttaaaaacacactttgccccaaacac

tattcggacatgccccagtgccctgacattgtgaaatataatcagagtaa

agaatgttcagttaaaatgttccgtactctttctcaatttggatagatca

gccacagccaagagtgagataacgcatgggggatgggtggggcgccctag

agtgttgtgggagaccgtgtgtgtttatgtgtttgtgcttgagaaatcgt

tcgtctttggcaagggggaagaggaaggcagcaaacagaacaatacgatt

gagaaagagttctgcaaagaactgcctgtgatccctatcgggaggctgta

cacaaggcgtcgggtgccgtccacatgctaaggtggccccattgcttggc

atgctaccgaggctcccctgggggccactgaatggttagtacagcgcagg

gatctgtaagttggtagtagcagtgtgtgtttgggtgtcatttgtgattt

ggcaatgccagttccagtatgtaatatcacctggttttccctagcatagc

cagaaccagttctgcagcccaccatggttttatagcacaccgtcgtcatt

ctgcagcagtagtgaaggctctcagagagccccatgtctcccacctgtcc

tgctgcccccagatcctgtctgtctgctactctctactctggtccttagt

gacaggggagaaacagagattggagtagatgttgtagctggatcctggat

cgagcagtggcctctgagacttgaggaaaggagtgcgatggaaggagagt

cagcatttggggccagctcacataagtagctcagagcccctcccagtgct

tggctccctacttgctgtcccactgagttccttgaaaatgaccagttcca

tggcaaagggtctgagcagaaacagcccagcggagacgtaggataagagg

tagcattgtcttagagcttttgaccagttgttgccatggtgatgacatgc

cacttcatagaattctaccggtggtaaggagatgacaggtatggaaggat

cttggcaggataaaggaccttacttattcttggtggaagctgtctgatgt

ttttgcaattcttctgtctgtcagggttggaggtggggtcatggctactc

taagagcatctagctactgagagaaagagagaagcatacttgttcataga

acctgttccttcttgggcccatttggaaagacccagcctttgggccttag

agatgaggagaaagacaaagacgtcagtgtacccttctgagtatggcaga

tggcaggagcttgagtctggcacagcccagtgatgggtgggagagggcat

gagttaatgctgcctggaggtgagcaaaggccggaagggcttgatccagg

atgtgtatagtttgacagtgaaccctcggtctgtctgtctgtctgcgtgc

cccatatgctgaaacctttcttctgaactcagatctgatttccgagtctg

agtaccagtgttaaagcaatgaggggtgttctagaaacatgattaggaat

ggatacagcaatccatgggtactgtatccagcagtccatgggcacaggac

atatggggattgtcccagaatcaggaatgggatgagactctttccagtac

caccccttaccaccacctcctgttccccctggagattactctgaaagcct

gtaggcgtgtgtgtgtgtgtgtgtgtgtgtgtgtgtgtgtgtgtgtgtgt

gtgtgtgtgtgtgtgtgtgtttaggagggtgagccttctgtccagtcaga

gcagtgacaacctgtcttctcctttcccctacagGAAACTGCACAGTGGG

ATGAAAACATACGGGTGTGAACTCTGCGGAAAACGGTTCCTGGACAGTTT

GCGACTGAGAATGCATTTACTGGCTCATTCAGgtaggcaaggtctccttc

agtgggccccctcctgccgttttcacccttaactcagagctggccctggt

ctagttgtcttcactgccaaccagggctttggtccactgtgtcccttgga

tcttccttccagttccatgttgcagaagagccaccctgagagggtgccca

tcagaggaacatagctgtatgctttcccctggggttcccaggccatcact

ggaacccatgccctgtgcgatctacctcctaaagtagactgcagtttcat

tagcagaaacagttgtggaattaggttctcgctgtctaatgaatctgact

attgggttcacattatacaagagcctttaattaaatagatatagaaaggt

tggaactcaaagctagttatagcgagctgtcttttgcaagttaattttat

ccaattctagtggggttgagtatacggttgccagttattttgctcatgtt

cgggcaaagctgttctgccccagagctgggtaaagaggggagaagccctt

gacaccctttgtgcctgatgttctgggtactgggaaggatcctgcagact

tggctaaagcaggggcagttattggtagtcacctacataaagagagcaag

accaaagaagatgggccgcttctcccctaacatacgagtgcaaagattaa

aggcgaagctactatagggagaaggttatgacctgacagggaagtcactg

gtgccacagaaggcaggctgcaccctcagcttaatcaccttagcatgtga

ttccaatccccactcgtgacactaatttgacactgactgtctgactgtgg

acaaattcagccgtccgtctctgattgtcagtgcagatgaggtgttgccc

tcacatcgaggtggtttttctgctttgagaattggatgaaatagtactac

acgtctccatagaaagttccctggcctgcatgcagtaggtgattcacagg

tattgataggatggcttctacctagacttccttccgtgttgcctacctct

gaaactgtcaccccggggcatgccttatcatcttatcctggatgcttcca

gcctggtgggttgtctgtggatgatggtgtaagttctcatcggcagctct

gtggtaccccactgaaacttagctgtgtggctcacgcggcttcattcaca

cagtggttaccgagtgctttggttctgtttgtcttctacctgcatggcag

aaaccttctcccacctttccaggtttttccaggctctttttcaaaaacaa

gaccacctcagccactccccactttcagtcagaggtaatcatgctctcct

ctggcacccatttctgtagctgatgctttgagcacaatccttcttctgtg

tcgctgttttctctgggcttgccttatctcccctcttgagaagcaggata

atggagtgcggggaaacttgggcacctacttcctaatgaggcagcttggc

ctttcggagcctcagcatccttgtctgtaaagcaggaacggttaactcaa

accctgagagcttagcttgtaaggctcacacaacctttttgcccctatgc

cctcggtgtggtgggtacttggtaagtattaaccgacagcaaactgctag

ggcacaggatcaggccgacgttacatctatcattgcttggcatcagcaaa

tgcttagatgagctaatgagtgcagcctaggtaggcatttgatgagcacc

tcgaagactctacggtcctgtctggcctcacctgcagcagaaatcctttc

atggccgaggggcttcatggaaaggggacaccctagacccactcttccag

gcaatggaagcagacgtgacttggccaccagctctgttgtgaacccgtgt

ctacatacagtattggggagtgtggctaagaggtgctgagagctactgag

ctctttcttttaggtgggtttggtgtccctacttctgacaagatagtgac

ttctcatcactttgctgcaagctggctcgtcccgagggggtgtccacttt

ggtcccatctaagtcaccacttgagggtctcatggtctttatgcttctcc

agcctcaggaagtgggctgtgttttcttagttttccattatatgtttggt

ccctctgaaacccatctgccaatgcccttgtgagggaggggttggtgatg

agttaccattttgttttaattcaccccggcacacagctacacagctcata

gcctgcagagtctgagactttctagtctgtttttttctttttcttttttg

tcccattgccttcagtcacctcactcctgcaggatgtcttgatgtccgag

aagcaccaaaagccagggagggtagaccacttcctgacctttgccccctg

tagagacttgaatctgctgcaggttaaggtctgggtctgaggggttcctg

gtcttctagcaaccacgacccccatagcagataagggaactcggttgcat

tacaggatataatctcagcacttgggaagtagaagcaaaggggtcaggaa

ttcaaggccagctacagctacatggcaaattcaaggccagcctggttact

taacaccctgtctttaaaaaagggcaacttttgtctgtctgtatgtctat

ctgtctgattgatctacctacctacctacctacctacctacctacctacc

tacctacctacctacctacctacctatcaatcgtgataccctggaacata

ccatagacagcatagtttttttttttttttttttttttttttgctttctc

tggtttgaaggctttgtgttaggaactgacagtgaggcccacctcaggat

ctgtagtgcagacccagtttatagacggtaaagggttggagctgggaaga

tgaatcactgcctagagcctagggagcctgcttttgtccttctcctttgt

tttttaaggacaggctgtgtggagacttgaactccatcctgggcaggggt

tctgggggctccctcagtgtttccgctgcggcaggccgtccttgccaggc

tcatcctggttgttccttctgctccaccaggctgcatgtctctcccttcc

tccctctctagctggtgctggccgccccaccccctcttggctagcacagc

ctgtgtttgttattgtagacctggcactgagccaggtctggctgcgagcc

agacttgtgggtacagtatgtacagtgtgtgctcagccaagtttgtcatc

gtacgcgattatttttttatatttttttttttctgatctggaaggggtgg

ggtgaaaacaggagtttttgcgatgccagaggagagaatgcactgaattc

catgatcttatttctgtaaggaaggactcgggtccggcttgccttacctc

ttctgcatctttccttttcctcctgtcctcttcctcctcctcctcacttc

cctatcagatgaaatcagcctgaaggagatcaacaaggcaagctcttatt

tgcagtgactagaatactaaatttactctcttaaatcagttttcattgtg

ttgaaattgacaatttgtttaattagaagtttgccctgtaggaggccctt

gtgagaggctgcgatttctcccctcacgtctctcttactttctccttttc

agttgtgctgggttttaagaccctaaatggtggtagtgaatgttcacttt

aattgaagtttcttgcccgatttgaatgctttaactacccaagtgtttat

gaggtatacctgatgataacagtatgtttaaattgaccaccatcaacata

actcattaactaatggccaatctggtccaaatctgccttttagagatggt

tttggggatcacaagggcttgaggattggggatatggctcgtagagggaa

gagcaatgacttccactcgagtttttgccaaatggtaggtatagggctgt

ggcaagcagcagaagagtcccagcccactgtccatgtccagagatgactc

taaaacctcgtacactgccatgttcccatgggagtcatccaggaagcatt

gacaggtccccatctctttgcatttgctttttcaaaagaggtgatttccc

gctgggatgtgaggctggacagaccacagaccacaggctgtgactcagtg

ggttctgaacctgccctgtataaaccttcccattcggcttatatgagctt

gccacaccttggcttgacagactgccagcttataaacacactacaactgc

ttgctctgttttggacacagctgtttggttggaggggtctgggtgtctag

gtggtcaggccccatcctgggggtatcgtttgcttttggaccgagtgcat

gcttaatgtgcactactgggtgaggctgtgtgcattgagcagtgatggat

gccgcagcatccttcccctcgaggaacttggtctagcaggaggcatcagg

ttaattataaaacagtggtgaaagagtagtaccaagtgtcaagaaggaag

ggttggagagcttcagatgatgcagaccctgcagagacggtgcgggcagt

gttccggagggatcacagaaagccagactgtgagaatggtgggcatcact

tggggtggggtctgtgggcagggtgtgtggaggacagaggaaggtatgca

gggcccaggacagaggcatgagaaaaggggaagggtagagtgatttctaa

ctccccagcactggaagactgcatattgagacaggaatgaccgagaacag

tgcttttccttcagaagggctgctgggctttggctatcaattggcctgga

ctctatgatatggggagacccttccagagtcacgataggtagctcatgat

ggccatggccatggtgtgggtaccctgagctccccacactagaataacac

actctttcccttggcctggtgctgtcagccatgctggttgagagtctact

cctgtgaccaggcttgtctccacactcagagtgtagaagaggaacgaagg

cttgggctgatggcttttacagttttcgtgggagcttggggtagcctgag

cttattctctgaggcctgagtcattcagggatcaggggaaagtgctagaa

aagttgggaccagaagcaagattggttttcttaggctgttgtgatggggt

ggagtgggggatacaagttggcaatgatgagtgagttgacctgcccctcc

ctccctctccctccctccctccctccctccctccctccctccctccctcc

ctccctccctccctccccatccatcacttagcttttggcttcagggttct

tacagacagaggaggaaggaaaggagggcagctggcaagtccctgtgact

gctaaatgaggggtccccacagtgatcatcagaaaggctgccttaagagt

tcaaagatggcttctcaggacctgcagctcggaattggccaggcacagga

gagtcctagtctataggcactccattcaccctgcctcctctctggaggat

actaactttcctttttgctgtcagggtaaaataaaaaaaaaaaaaaaaaa

aaaaaaaaacgggagacccttggatatatcctctctggggctctttaact

ctctcctgggtcttgagaggtccccaccctctgaacattccttttgacag

tggggtgtctcggaggtccacacactggggtggcagagaggaacaagcag

ggaaagtgctccccctcctcctccctcttgaacacactcttcgctcttcc

tctccctcctctggatttggccagcttcctgaactttttcccagagtggt

gtcgtcactggcctgccggcaggtctggatgttctgccagcattccagag

agtttatgatcgcttgcagatgcgtctccaagaacacactgtacccaaag

cagaaattaatgcaggcttgactgcggtccaagcccctacacgggagaga

ggagaaagaaaacaaaaactgtttaatgctgtttataaattatacactgg

ctgatgaaaaatacatttctttcctgcccccttccacgctggccccttcc

ctgtggccccctcctcatccccccaaccccgccccttctccatttttgcc

tccagagcctcagctgagcgcaggcgaatatggttttgctttttagctcc

tgttttggctgggatccgaaaccctgcctctgattcctagcctgagtatt

ttttcccctttacctgatgaaattcactaatttttccagcccacatgggt

tgttttgggttttgttgtttgggtgggtagtttttttttttcttttggcc

tcgggttttgtttacagcatgtgtcatatgcaatggcgcagggtgtcagg

cttactcgtccctggaggataagtgccccctccccacatcttcccacctt

agagtcactcagaggtcccaaagccttcaaggcttcacctcaggctaact

gaactgagaaagttggggacattcttcacaggtcattgtattaaacagta

tttttccaccagttctcttccagcctttcacctctaacccatgtgaagaa

ggggttacatagcccccagtacacacatctctacttacatcagtgtagca

ggaccggagaactcattcctttgactcttctggaccccacccagcttcat

cccacaccctacaagccccctcccatcccaatttattctataaattcgtt

tctacattatgtctatccacttacatggcttttgtgttttttaatactgc

taatgtttatcatttgcccaacaggctcgacagattttgcttcctgtaga

gcattgaggctttagagccccctcgttgctaagtagcagtctctctgtac

tgtaatgggctgattttgtattctgttcagcgctgtatcttatttttgta

tgcaagacaaagtgttttgatggaggacgattgcagcagtgtctggaggc

tgtttggagttttatgggctgtacagtacactgtgtgctctccgaacaga

acacaggctgttgaattttggattggatttgtcatccccccccccaacac

cctcagagaggagcctgtcagatagaacatgacttcaagggtggaagaaa

gaaagtgggaattatgtaatatcttctttctgtctcccattcattctctc

tctctctctcccccctctccctccctccccctctctctctctgtctctct

ctctgtctctctctctctctgtgtctctctctctctgtctctctctctct

ctctctctctctctgtctctctctcatctctctgtccccaaagaaggtgg

cacaccccttccccattgcagcatgattttgcacagctaggtggtgacgt

ttctattaggaatgaacagtgccctagaggagagggtgggacatctgtcc

gactgctgttcatttggaatctttctttgcttctttccatcctgcttagg

tcccgggtgcctttactagcctttgatttttgtctaaggatgagtagcat

ctcatggggagctgcaggacagggctgttttagtgggatggacctgaaag

caatgaggtttacatattctcagagtcctcatatcttggttgggttttct

gcatacgctgctgccacccacccctaacagaaatgtgtttctgggtggac

tcccgtagccatcttttcctctagcttctgctccctagtccttgagccag

gcagggccagggttgttggagtccaggtctgtgcctgccctgccactggg

ccgctgctgttcctccccgtgctccacgcaggggtcgggtctctgcctcc

ctccctctgcaggtgtttattttgtctctctctgctcctgcttctccccc

aatctccttccatctcacttcatctctttggcaccgtcctgtgtctatgt

cagccacagccgctgtttgacactgctgcgtgttttctgtcttctttcct

gtgtcttcatctgtctctgtcactgactgccttagcctgccttctctcat

ctgtctgttggtctgtctgtctcctttcttactactctcagtgttcctga

gcttagaagttagacatcagtcagagtagggaaacttaggtcattctggc

ccaggggcctggtaaggacacgtgactggagagagcctgaggcacgtgtg

catgacccttcacattgaaacgacaagagtgggtctgctcagccatgact

tcggtccttacacactattttttaagtggagttggtagacagaaaaatgg

gcttaggtctgcgaggtccttgttggacactgtttctcaggatcccatcg

ccagagcccgtgtcgacacaggtgtgcgctgtcacctaccagcgcatgga

agcctctagccgacccttgctaattctcctgtacttgtgatctgggaaag

ggcttggcacacagaaggcttgggttgggatttttgaatcagtgactatc

atataactaaaagtaaggtgtctcagtgggtaaaggtgcctgctaccagc

ctggtggcctgagcccaatccctggaacccgtgtgaaggtggagagactg

gaccttttgcagaaaatgtgttgactgtctctgtgcctgagagaggggac

ctggaggtgatagagatgcctgtagagctcttaaggtttgctctgtggaa

ctcaacagattcattgggcccaggctaggttgggggtaaccttggacagg

tgtttagttaagaaggtagctccttgttggaaagtctggagaaacagcca

aaaatgaggggtagaatcttcaccagacaccgagccccagcatgtgtctg

tgtgcacacaagtatgaggggaaaaaaagagatggagacgaagcatttac

atgaaatactgaaaattgtttctttgctgtaatgggtccacacaggccat

taccttgaagatttaaaaccaaaatgagctcatgatatctactgactgcc

acggacatccatgcctgattgcaaagcccgctttacctttgaatttcggc

ttcattgttggttgttaatctttggatgttggtggcaacacacacacaca

cacacacacacacacacacacactccttagattcctacaggaacaagtta

atggatggatagataggagtgtttctcaacaagaccccagtgaagaacct

ttccattttcaaaacggcctctgacctctcaggccagctgagcagagttc

ctctgaggaatccagagaacagaggccgagactctgtgttgactttgcgg

ggaggggggggggagcggaagttcgaatttttttaatttaataatttggg

tttcctatgaatggttctgtgcagtgtgcagtagtatgcattttcataac

tgtctcttctactttggataaaaggaagggagggggaaataacagcagca

gcgttctgtgggcaaaggaagaaaacaagagaatggggcagagaacaggc

taaatttaaaattgtaattggctgacttccactggcttgcgggtgtttta

atgactcataataacttcatttaaaaccagctgagcagaaaatagattgg

agaggagcctcgggccattatggatttgttttttttgacaagctcggttt

tcagcagccaggaaggctctcagagagggctcggctgtttctctctctct

ctctctctctctctcatgactgagattgtgtgcatgtgtatacacacaca

cacacacacacacacatatatatgtatatcagagatagacagtttctctt

tctttttaactcttcccccttccccctttctaatttttttaactgattaa

aatagatgcctcggggtattattttagcattttagaatcgctgctcccac

acccacttttgatgtggtaaacagaacataaataaaattgttttggagat

gattgaattaattaggtagtaaacaactccaaagcataaattccctgggc

tggtttgttcccaccctcatccatattttctccaaccccctcctctccct

cagcccctccttccacaggacgagaccctccctttgactgggcatgtctc

ctctgccacaggggtagctgtttggtgattcacctgcccggcacaaattg

atgagattatgatgggactgggtctgtccaggaagttttaggaattgcca

gaagaggctctgacccgcctgtagttgtttctaaataaaatcctgtccgt

ttcccatgctaggaacagagccaggaccagagacctgctccctacttttc

ctgtcttttgaaagtcagcctgctcagccccagggtgagcctggccagac

agtgcaggaggcctcttccatctgattggtctcagtcctcctccatctga

ttgccctcagccatctgttctcccaaccgccccccacccccgtaacctgt

caccttcttggttcaggagtgttttttttattcacttgctcctttcatgt

actagaacctaggtttcttggggacatgtgattcatgtatttctgcgttc

gtccatgccacctagcctaggaacacctggcctgtgtaaatgggtggaag

tggggcaggaagtgcaatggaggctctcctttgctgcctccagccctggg

aatcagccgtctggatccgaagagggaggcagagaccgtaatagtagcct

atttgcttgcctcactacagtctagcacacagtaggcagtcagtaaatct

tgcctctctacagcctagcacacagtaggcaatcagtgaaaatctactga

ataaatggctgactttgggctgatggtcctggattgggacacagagaaag

gcacatttacttgtgcctgctatgttccagacatcgtggagtgctggtac

tctgacagctctgacactcaggtgctcctttgtagatgaggacactgaga

cttacagcatccactgctgagcctgtctgagatcagaaagctggggtggg

gggttggccagggctgactcccacctagatctctctactgttgattgtca

ggattcttgcttttccatctgttgggctcctggtgccttaaagattgggg

aggtgagactggagagctgagaggaggaaggcctagtctttcgagactct

gaggtagggagcaccttattggatgctgctccatttggccagccaccagg

agcaggtgtggcctattaacctttcctgctgggccagggcagcatgtgtc

cgctggttgcctgacctggccactctttggagggctggaggaaggtggtt

ctgggcgcatcactagatggttcgtaggtcagacagcatgcagcttcaac

ttcatggagagcttgacagtggcatgccctttctcctctccatcaccatg

ccccactagagaggagggagagaaagacagctacccgtggtctcatatct

tttcttggggaaggcaccctggggttgggagtaaatggcatagtcaccat

gtgctgccactctgaaaagaaggagcgtgtggcatacacccagcacgtgg

agtgtgacttatccctggcctttcagaccttgtccagtggagctgtatcc

tttgacatgagactgttttgtctactgtagaacaaccttggtgtccctcc

aggcacacactgggggtgctggaaccttctctggaggggtaaaatcgtta

cctagtggtaaccaggtttagaaggttggccagcagaggaacttgcctga

agtgctattctggaacccacctacatgtacatgttttagagagaggagag

cagctatcacatgtatgaatttggctgttagcagctggactgaatggcca

gtgtgtgcaaacccaggcagggcagatgagagttagtgctactgtcctga

cctgccttctgctagtgagccagctgaaaggatcacatgagcctgggaag

ggaagggtgttcactggccccacaagggaaacaaatggctgctatgggag

tttggagctatgagactgtcccttggatggggttggtagggggagtagtc

cagaagagggtgggcagtatttaaatggaggaagagcatcctctggcagc

cgtgtccaggaggagctcagccgtgaggaaaggggctatcttcattttcc

atggtgaagagagcaagaacatctttgagctagaaccagctaggcaggca

gcacctctgcacttctgcctctggtgtccacagtgaacatcaaaggttta

taactcacctgagggtcctccagtatagtggtcctccttagtggccagtc

gagaagcactccagtgaaaaagaagactgcctggtgtgggtaggtgggga

cctggcatccagcacctcagcacttggaagacagaggcaaggggattgag

aattacaggccagctttgcattagaagactgaggactgggagttcgctta

gtagtaagagtacttactcagcgtgcacaaggagctatgggcaatctcaa

gcaaaccaagagagggagagataggtagatatgagagagggggagagggg

tcctgcaagcttcagcctgtgaatacagtgcatacagcattgcccttggg

tgaactgaaactactaggttaccctaaggacaaatagaggatctatgagg

ccttctcccaggtgaacgtcaccccaaatcccagaccatctctttaggtc

acatggtaaatgcccttatgtgtgtgtgtgtatcagtaagactgggctgg

ctccctcgggatgaaggcagcaccaagggcttctctaaggacttggctga

gtccgggatccagtatggtacacgtgcctcttctagactcagccccttta

tcctggcccctagtagttggcacactcctttctctttcccctagccaagc

ccatttggaacagaagtctttcacttgccagagaaaaagaaaaaaaaatc

caagttctatacttttcattaggtagttgggataaaaagccttctggggt

aggcggattaaacctcgtagctctcttgctggctattagaaactaattga

gcacctagcgctcgctcgttggcactgagttctgctggcgtaattatgca

aggccacagtcatttcagtaacaaggtaactccaggtaaagagagaagga

tgccctgaaactttcagatttttccagagtcttcctcagttgctttcctt

aaatgagcctgggacagacaaagcccggatatgagacttttggggtaagc

tgttgtcttgggtgacgactcaccccagcctgagttcttccatggaaacg

gcccaggatgtgtggtagagccctgctgtgagctcctttggtcttgattg

aggttggggacctggagtcacacgccttggagtagaggatgcagagaagc

ctcttctgcttggtagacagacgagccggcagtaaaggggctgaattaga

gagacaatagacccccagggcagcgccttccacaggcatggagggcaggg

ccagacacgagccctgtaataaggtagatggttactttgtgtctatctgg

acatttgtccagtgtagatgggtaaggtgttagtctagaagactagaaac

agctgggcatttaacgatgcctgaaggtttctataccgtaggcttattga

tggtgatactttctctgccaggacgacatctttccattgaatcctgcctc

tctccagagtcaggagctgtgagtagggcccgcttccctcggggcttggg

ctcagtaagtgccaattggaccttgactatgttttggaaggagccctagg

gcatggccatgatcctgtcctctgggtggccctgcctggcctctggcctg

ccacccatcagcattgaatcatgttcctaggagaacagcatgtaactagg

cttcccaatatagcccctgcctgcaaggtgacaggaagtcattgtcactt

cgggcactccatcttctgacaccacatggctggcctggctgtctcccttc

acttctcagtcctgcacagccatgaattctgctctggggggcctctgtaa

agctgggttttcttttagtttttaagtgagccatttccccagtgctgttt

tcaagcctctgccttgagtctccggcctggactgaattgttcccaccgtc

tccgcaattataagtttgtcacagtcagaggctggctgaatggaggcaga

aatgtggtgtttggttaaattgtaaaattagctacgaaagttaatggagc

atttctgcctctgcctttcaaaaggcaggagtcttaggaaggtgcttcag

gtaccttgcctgagaaggaaaaggggtgggggtggggtccagccccgcat

ggtggcacatgcttagaagttgagacagaaggattatcatgagtttgaag

ctggcttagactgcataatgagtttcaggcttaccaggattgtcgtaaca

acaaatagagaaagaaagaggggcaggagcttttggctcaccataccttc

ataagacctgctttcctgtccatagttcatgtccattttatggatggagc

catgctcgttccttgtctcagctttccgagctaccacctctccagctccc

aagttcacgtggtaccgcttgcccacttgcaaatgctgaccctcatgctc

tggaagtcacagtctcagaggtgcgtgcggaagcgctgccaacagtctat

tttctgcacgcaccaaattcgcaggtcctctaattacaccaaacaaaccg

ctctgttttcaagcagcaattcgatgtcattttagttgtcaggagcactt

taataacgctatgggcttcaccctggacatagtcccccccccacacacac

cccttgaaccttttgtatagtgtgaaccagaaactgccagtcatgttttc

ccccagttggggagatgaacattaattgtgacaggcaatatttttgtttt

gagaagtgtggcgccttgagccttcagaattttgagcccgaagccggcag

tgctggggttgtaatgtaacacctcaattatggtgcagggggccagaaag

ccagactctgcaaccagcaacgttccttagatccatggctgcagccacaa

ttagggtttcaaaaacaccaaaggcaagccaatgagtgtcatttctaacc

ggtttgaactttttactactccctaacatcagaagcacccggctagagag

ctcacttcttgtcctcaataaagcgattaagttgaatgggtgttggggag

tttttaggctgagaagtgctaaaaatagtactgctctgttttctgaaagc

cacagcaggcctggaaaggctggcagccgcaaggtgccactgaggggttc

agaggaagggagagggatggcggcagacccttggggtataaggtccgagg

aggttgttgggagtagaggcaaggagatcagctgcctccaaagtgctgtc

acaacagcaaagatggcatggttacttctctcttcatttgaagaatcctc

caggagagaggggatgaggagagaacatgaaggtattaatcctaaattag

attggcagatccaaagtttagaatggattgctaggcctaagtttaaccta

tttagcacttagcctgtgacatcagctgtcctagggaaagggttcagtta

gggcagtgagtaaatgttctggcctggtatcagagaaggtgggggtggga

gaaccctgagagccaagagggggatgctttcaccgaagacttggtgtacc

agggatgcccaggaatggaacaaaatgactttttggtaattgacggtcaa

ccaagacaatcgtgtctgctgtagtcctttgagagtggggggtctttttt

tttaaacgtgagagcccatgtctctcctttaggaattactgttgtttctt

gacagggcattttgctgccatagatgccggaaggagaatgttggatgaga

gacagcctagcctggacactggggagatctgggtgttgcttcctggtggc

atttctagaggagaagagccatctttggggcttgtagagctcacagtcaa

gggaccggagcagaatgggcctgcctgagccctggcaagtggctcctcta

agtagtgctgtggcgatcacactggagatagtgtctgaagtcttcagaaa

gactgtgtcatgccaagtgttggaacatgattactacttaaggggaattt

aacacacacacacacacacacacacacacacacacacacacacacacaca

cacacagcattgcgagtggcattggagagagatggaaggagtagggtgag

aatggaagattctagaaagagaggggtgtgagagaattgtctgtacagag

attcccaaagagatcaacaccttgtgagaagacagggcatccttctcggt

gagaaaagaggaaactgcctgaaggaagggaggaccagagtgttcctgca

ggaggaaggtgtcccacttttggaacataagtccaaacttggtgggagtg

tcagggagtagcaatggcacacacaagcttgaaggtttatagggtaggca

gggtcctgatcctgacttgacatctttgaagtagccctggtttccttcct

tccttccttccttccttccttccttccttccttccttccttccttccttc

cttccttccttccttccttccttccctccttccctccttccctccttccc

tccctccttccttccctccctccttccctccttccttccctccttccctc

cctccctccctccctccctccctccctccctcttttttcttttcatcttt

tacttttttggcaaggtttaactgtatagaccaggctgccctcaaactca

cagggatccccctgcctggccctctgagtgcccatacatgactggttttc

tttttttctactgtcccagaagccttgtgagtttgaccccgggaagcatg

gtccatgcaagcaggtgtgtttgtgtgctcacacgcatgcatcagaatat

cagcctgggaccaagcagtctgagggttcggcagaaggagtgtggtgttt

aaggaatgataagaggaggtcagaggctcaggtgtcagacagcatcgggg

atgcttggcgccaggagaagtccctctcacttctctgatcctctcacctg

aacgtcccaaagtgagctaatcaaggaagatgtgtagagtttgcgtagtt

ttcgtatcccataaaatggccgaaattcccgttgttgtttccctggttct

aaaaaagaacctagccaagatggcagtgccaactcccttagataaaactg

ctcccaacacctgaaagtcctatgcccctggtgctcaggatcagtctcca

ttgtggcctttttagtgcatcctgggccctggctaatgattgctccagca

ccagactctggtccctctgttccatcagtccacctgatagatgagagtat

ttacatcagggagaattccctgccaaaaggagcatcccagccctcgctga

gtacagccagtcagccttcccagtgccgaggctgtgagcagtcacattcc

cttttgtatgcatatcaagctttctttgccatcctaccatcatacctcac

tcctttcagtttttctcgtaactttagaactcacagagttctaaagagtg

aagcaaggctcacccatgctctaacagcctaggcagcgacagagatccgg

aatgaattttcttcatagttttgggggatcatcttagttgctcttgagct

gttgatttaccatgctgtgtagaagtgtggacttgtctaccctccccagc

agtgggatgtgtttacaagagcatcaaggtagggtctcatctactcaagc

cattcactttcaggaatgggccttcccctcaggatttgaggacacagggg

aggcacaaacctaggggctgttattcctcagcttgaggacatgaatctag

tttgtcaccactctgtgatgacgacttggacacctactctgcacccaaag

aactgtgttcttcacagtttcttatttaaatggttgtatgaaggggaaca

gtcccctttcaaagggagatctaaggcttcagagtttatgcggtgccact

gacagtacaaggaacccgacgccaccgcactgtaggacttccctgggctt

gaccattagcttcggtccccacaagtatgatgtgtgtggaaggatgtctc

acccttcacagcccttagagtaggagtcattcatggggggagtgggttgg

agaatatagttaggtgagatgctccaaaccggcactcttagggaggagga

ggaggaggaagagggggaggagttgtgtgtgtgtgtgtgtgtgtgtgtgt

gtgtgtgagagagagagagagagagagagagagagagagagagagagaga

gagagagaaagagagagagagaaattggtttaccatcagatctggatttt

ctttaaagtaattcaacagtcagtggacttgcatgtaaccttctgggtgg

ctagaagctaatgatgatgttaaaaattgcccatgatgggaacagactgg

agaggcttcttgtctctgacgtcccagaccctatctgtccttgtaactgt

tgctttgccctggggtcccgtaggaagcacagtgcttagtgacactttgg

ctgaatgaagaattatcagagtggacgacttctctcctttcttttccttt

tcactttcctgggttgaaaaaaatgtatcctaactgttaacagtttgtcc

ctggtacacataggaaacagctcagggtgacagctaggaacagtagaatg

gagacttgagcctagatttctttgattctagaatgcaagtttttaataac

attactagtcagcttttctctctctctctctctctctctctctctctctc

tctctctctctctctctctctctctctctctctctgtgcgtgtgtgtgtg

tgtgtgtgtgtgtgtacaccataaagctagactctgatactaagaactat

ggcagaagttctttcaaagcctttctggttcaagtggaaagactgtggga

caaggtgctgttcttcctatgggacctgagacagagactccaggattcct

gcaaaatctgaggacactgcaccatgctgcccctccagctgcagtagaac

attagttgggtattgtcccctcagatctagcagtgcattggtgttgtccc

caacccaccccaaggtttcaccttatgcagagtggaccctgagctgtaca

cagagtaccagcattttcccaactcaacagctggatctacacatggccac

ccttaacccacagaacacccaccataccctaacaaacatttggctttctg

caccctcccttagatttgagaccttgcatccctggcctctctaactcctg

ttctacagtgagtatagccagtgtccccactgcagtgccaactgtagcac

tatagagccacatcctggaccaccctgagccccgtgggcttccactcagt

gccatctgcccacctgtttccccctttctatcccgtgtcccttacaaaaa

ctgatccttcccagttttttactttgttgataaaggagaaagcgagagct

agggagagccaaagcgggtgtgaggctgcaggcagctctctctgggagta

cacagctccttgagggaaggaacacgcagtgctggcctttggaactggca

gccagtgtgctgttctctgtctgataagaggtactgtaaataaaactgta

caccgtggcctgttgtaaaatgcccgagtctgtacgcattgttctggcgg

cttaggctttttttgggtctgctgttttggtacactctgtacttccttat

gtaagcaggcatgcatatctcatcagaacattcaagatgtttattttaaa

attgagaggaattaaaaaaaaaaaaaaaggacacactactcaacatcaga

tgctggcaaggcgctaggtgtccccacccccatcctctcagtggttcctt

tttcaaagattattattagcttgggggttggggaggctttgaagagggag

gggtttttgaatttgtttctttttttttttttttttggcttcagccttgc

ttccctagagagacatcccgctgcatctgaatgcgtgcagtatcttttac

tgatttctagatacccaggattctaacccctactcccagacccatttctg

gttaggaagatgagggagaagattctatactttgccaaaaagattaggcc

agaggaggaggccgctatggggagtgtcgtggctttcactgggcctccct

cacctctgctgcccttggagtactgacatttagataaagctttatgtttt

tgctaatgcctctgactggccttgatttgatttcctgtgatcctgaaatg

tgggggaggggggtggttcaggttggaattgtccccctttccctttgcag

aaaccttagagtagctccagcctcactcaggctgttgtgtctacatcttt

tcttctcaccaaacctttacctgattttacgtccacctgtggcttctgtc

taacctgggtggtaccaggatgtcagggcacagaagagaaaagcgactat

ggaaaaccctggctattgaggcaactctacttcagtgtgatactgagagc

tttgggtatttggggtggggggtgggaatcttgcaggagcaaagatgtct

gtttgtctgtctctaactatcaggcttactaacccccaggtaaacttaga

ggtgagggcgtcccccaaagcagatcactgccagccagtgagtgtccatc

ctggtttgctgagggaagtctgcctggggatgggaatatactgaagggaa

ggggcttgctgttgtgaggtatacagtaggccaagaggaaatgggagccc

cgcttgtctaaccttttcttcttttcctctgagcttcttgagtccctgcc

agaaacagacatgtcctagagtttctacaaagagtctgtagagaatccag

aaggtagagtcaggactttgccaccctccaggtaacagcctggcagtggt

ctcaatgcccacagtctccttcccaggccagttgtataccccagtctacc

ccagtagagcttccagtgtgctggggagggcaggagagaaaccagaagca

gacccattgcaggcggctatatggactcatccctggaagatttcagagtt

gaataactgatttgttgttgatgctgaagtattggtcctatgacagattt

ttctgttcctctcaaaacaatcactcccttttaaatcagtgtgtgtgtgt

gtgagagaactttgatatacgcctttacaaacgtgagactcaccttatac

aaaataaatggccctcaaataccattgcattgaatctcagtaaaatcaat

catagtttaattgcaacaaaggcagtgcttgaaagggatgctgggtggtg

attgtgtatgggtcctctgtctctacaatccactcgactcacttgtgagc

ttgctgagggacctgctcttccgatcccattttacagatggcatggcatg

ctcacggttagctcccctgtgtcatagctgctgaattcctctgccaaatt

tgaaatactctgtcagacccagcttcgaatccctttaccataacacagcc

accatgggcagcattaataacacctgtggtctctaagcaacaaggagtct

tgtttttgttaatatttggcatctgcctcaaagaaacaacgggtcagcga

gatggggcgctgcctaaaggaacttgtcaccaaacctgaggacctcagtc

caatccctgggacccacacagtgaaaggagagacctggcctccacattcc

ttccatagcatgcattaccccacactaataagttaaaatgtaatttgaga

aaagtaaagagaattaggtagatagggtcaaacccatttcatagccaggg

agccataggctcagtaaggcgggaagacttgagtctccgggtgacatcca

gagcatcaggtgtctgattcaggcctgggccctggctctccttgcttcac

tgtgtttgacagaaagaattggatgggtccagctcctcgtccttcctgtt

gctgcccctcttcaagcatctctttctcacctctgcctgacattcctctc

acagacccccacctctatccggcctgatctcctggttctttccctgccaa

cagacaaatggccttgtttcttcctgcttcctctcttctacttacctcac

aaagatgccaactcctctgaggctgctgctacagcctccctaaaacatga

gctctaccctttgtccctccccacccagcttggctctgtatcacacacta

ctacctggcttgtattcttttctttcttttaaactgcgtttgcatcttgg

tcacaagctctgggaggacattgtcacccacctcctggctttatggatgg

gtacccatgaaacagcctcatgtgggaaggatgtgacagccatgaccatt

ttggagctgtgaccctggagttcttttggctctgggctgagccccagggc

attttatatgggtagatgttgctgctagacccatatgcactcaccctggt

ttggctggcatggccagttttgacaccccatcctccagcttcccattgat

gctttctaaatgtatcagagctggatgatgccactgtagtgcatttcagg

aatggccttgtggtttatccacatttgagttgctttaaatgcaaacctga

catcataccgaaggcagcagtgaacaacatatgcacagtaaacaggcaga

gactgtcttattgattaacagatgaggcctctattatcgatctaggcttg

gcgaggaaatttatcacttttaatttcagggctgccaaatatttctgcct

ctgagccagccattagagagacaatttaatgtacagcctacttgggagag

gggaattcatctgatgcgttctgccctggtctcagatcctcaccctggag

agcctggggtgcaagtgtctccaggaggggagccagcagtgggagccatc

gtctgtggtcccaagtggaacttgaaggctgacttggagtcccttgggaa

gagcttaatatcgtgactttggtgagtgcggagtgagggggacattgaag

gccactggatatggtcaggctgggaccgaggaagtagtcatttcgtagat

gctgcggtgtgcttgtctgttttttatcttcatctgaaccccgggtgata

gtgtatgttcgccctttgtttcctccctcccccttcctctctctgtgcat

ctgccatgaaagtgagatgctccctacagccccattgatagcggtttctc

agaacctcgccatgccacatccctttgaagctcacagcactattgggttc

ctaactcgattcggattatgactacggggctccaaagcagtggtggagcc

cacgaggtgcccacccctcccattctttcttcagggagcggtgagaacag

agagccaaagagaatcagatccctcgcaaatccacagaaaaatcattgat

ggctaatgattcaggataaggggaagttgctcattgtgaggggctggaac

tccatccagccccagcccccccatcctgaccctctccgctttttgaagca

tttcctgtgtacccccccaacccctgtacatggatgaaagtggtcttggc

tgtcacttccaaacacacagacactgtttttgccaccgtcctcatagacg

ttttcttggcttgattttcaaagctctgtaagccatgggccaggtgcttg

gcctgaggatatcctgtggctttcagggacactccaaggttgttgtcaca

ggcttcctcctgcctccaggtaaaactcagcgccagtcacctgaggcatg

caactataatgactctgaagtcccttcaggaccatcgtatctgccctgga

gtgctttcacacccgttatccccatcagctcttgtgctgagcacgactag

ggtacatatagcctgtttgttacttgggaatagagaagtcagtgaggctg

ggatcgtccttgttctacaggtctctactcatcattgacaagaactgttc

tttggtgtttaagtctctgtattttgattcttactttttaaggcacagat

ttttcaatttattcttttgacacaaaattctttctctgtagcccaggatg

acctggaactcaccatgtggcccaggctgatcttgaacttgcaatccctt

ctgcttcattttcctgagtgctaaccttacaagtcagacatgtccaatca

tcctcgaatttttaaaacaagtactggaacgatttctccaagtgcagttt

aacatgaagccctgatacataacatgggtaacggaatgtggttttgttga

gttgttacacgggaatgagacacttgcccagttatccataagtcttcttt

gcagatggaagcccgaagaggctgacaataaggaggatgaagagttcaga

agtcaggctgtggatctgtgtcctccttcgctctgtgttacaaccggcac

atccctaacctctctgagtttgtggttattcatctgctgagtaggacaca

gtagtggtgtagacgccaaatgctgtcgttaggacttgacgtagtcatgc

atagtctgcattcagcatcatgcatggtatatagtaaatccatgagaaat

cctgagacagagaagagcagtggggactgcaaatgtgcaaaaaatattgt

ccggctcttacaaggctataaggtatgcttggacacttactaagaccctt

gaacacacataaggaagagctcagatactttgtacagagtggatcggaag

ggatggaacagctgtaactggagggtgtgcccctgatgcatggtcttgaa

catgaaatggcattgaataaacagtagtgagaccagaacagagacactag

cggagtcaggggccgtctaaaaacggtgctaacggacagtctaagggacc

gtctggctgtggcgttgtttggctcactgggtcaggttatggaggaccct

gaggtacaaatgaagaaattgagaaccagtacaaatgggaagtttcttat

ataaagaagtatgaggagttatatgaccttcaatggagaatcggaccatg

taaacccaacctcgagtcttccagtgtctttggatctttcaggcacgata

ctgcaaaggtctggataggtgagggtgatccccacacagtgaagcgatgc

atattgaagacattttgaagaaaaagccccgagacttcaggctggtgacc

cttagatggcatgcagaaaatgactgggctagtggtgtcatgtttcacgc

tgacatggtgacgccattgctgtgagctttgtaaatatggctcttgcttg

gctctaagaacattgatggagtttaggttgtcataggctcccactatgag

tcaggcagatacatctgtagacaagatgataatggcttctcagagagtca

ggctcacagggtaacagatgagtcaccttgcccagccctgaaagctgaag

cttcagtgatggctggagtggagtgttaaggtctaagggatgcagggatg

cagggaggcatggcagaacgggtgctagtgtggaggaaggcaggcccttc

agggagtcacctcacttgaggtgggagatgaagcatggaagagaatgcca

gcaagggctggtgtgtcctagggaggagtttgggcaccatcctcaaaatg

cagggaaagtttacagggcgttaagtggccaggtgcaagtggtagttagt

gggtcacagatgagttaaatttgcttgtactgagtcagagagtgtggaga

cagaggtaatgcatactgccttgtgagatacatgtctctccttgttggtg

tcttttgttcccatccagcagagctggcctttcatcgtcatggggcctct

tacctagactctgtctaagttcatcttagtcatgctggttttatttctcc

ttctctgggagacctgggtgggtagtcacctgtccaaggtagtctgcact

agctactggggctatggagtatctctgaatctgaatcttcaccggtgtcc

cgggtgttcccctagtactcaggacatgccccaccctctggactatggtt

ctaacgttaccatttctttctttcagCGGGTGCCAAAGCCTTTGTGTGTG

ATCAATGCGGTGCCCAGTTCTCAAAGGAGGATGCCCTGGAGACACACAGA

CAGACCCATACTGgtgagtgtccctgactccattccccagaccaccaagt

attatagcccaaaggaggtcaaggtatttggtgacatcctcttactgccg

aacacggtactatcatgccgagcgtctctgtggatgggaatgggcttgtg

tagacgcccgtccatgaggcttgagcaagcattacagataggacagttct

gagaccagctgttctgagttaggccaagacactgtctgtcataaggcaac

gagcttccatcttcccacggtgtcttgcacgttcttgctgaggttctaag

gattctgtggtgttgcctaagttcccctcgctagtggtcggaagcctctt

catactcaactctggttaaattcagaagccctggtgtagccacagtggct

gacccgccctctctaacaggttgagccgtcagctctaacacagccatcaa

cacgcagggatgtatttgggctgtgttaaccgccacagtgctgattgcag

tgtcagcatcatctcgtcaccatgatttgacagctctgttgccatcgtgg

ccaaagttcacactggttggttgctcacgctgtgccaggcactgttctcc

tttctttccatgatccctcttggccagtctctcaggactcctgcagacta

acagggaggctactcttaggtcagaactgaagaaagggactttttttttt

tttgagacagggtttcaaaatccgcctgcctctgcctcctgagtgctggg

attaaaggcgtgcttcaccatgcttggctagaaagggactcttaaaagag

gtcagggacttgccaaagtcagaagcaggagtgtgatcgttcagaggttg

gggtacccaggtttcattctgactggggcagttcctaaatggaaccgggg

attcattacttagtctctcttcatttgggtttcctcatcgattaggaaag

caggggcatgatgctatgggtctatcagatagtgctttgtgttgattcag

taaaattattttacctgcagtgctccccagagtgtttgactcctagtagg

tatttagtggatagtagttgatggatacttaatcaagtactttttgcaat

cccacagtgtgtccagactgctggtggaatttaggatatggtagcacacg

agacgcaggcaattctgactctcagggaactgaggtggctgctgagacat

agtatggaggacaagcataggatacgatgtcaattcaagtgggtgtcgcc

aagaaaaaaatagagcagatgtgcgggagaaaacgagagggagaaaagtg

gtggtttggataaaatggtcagggaagaaggcttctttaaggggtgaccc

ttgatcagaaatccaaggtgaggggcgagctgcttagactcccgtgggag

cagagggacttgcacttggaaaggtctggaggccaggcaggggctcataa

tttcagaagagcagagtgaagaacggtgagcagagtgtccagtgagatgg

gaaatggggccggcctctccgccctagagcctgggctttactctgtatgt

gctgggaattctcctgagcggtgtgttagttactcgtttcattttagcac

tgtgactgtgtacttgagaaaagcggcagaagaacagagagtttattttg

actcactgtctgaggttacaagtctatcatgtccctcttcagttaaccct

cgttggaaacagcttcccagaccggcccagcaatgtgtctcccaggcgat

tgcaaattcaggcatgttgacactgaagattaacgatcatgagtcaaggg

aaaatatttgcaaatcacatgtgaaatctatagctaggaaatacaaaaga

gatcttacaagctgaaacccaatacaaataaccagagaagaaaataggta

aaggaaatgtcacataactccaaagatagacgtagccgaaggacacataa

aaagattctccgtgtcattagccaccagagagatgcagatcaaaccgcag

tgagataccactcagcccatcaggatgtggctctccgaaaagacagagat

agtggcaagtgttgctgagggtgtggaaaaaccagagtccttgtatgtca

ctggtgggaggggagagcaaggcctcgtcgatttggaggaacacctggac

gctctcaagaggtttaaacatagaatcaccagattacacagcagttttgc

tccttcctgtctaccaaaagagatgaaaatgtgtgtccatgcagaaactt

gaatgtgaatgtccatagcttcagtcaagtagaataagctagtgtcaatc

aattaacaggtcagtaaaacgtgaggtatacacggtcaatgaatccgttt

ttgttgcgaaatggatggaggtattgatacacgctacagtgtgtctaaac

ctgaaaaacatctgttggaagaagcctggcacaaaaaagtccatgtgttc

tatggtggccttcatttatatgtaatgtccaggatggataaacctgtaca

gccctggagtacattagtaggagtcagagagcacagatgtcaggaataaa

taggggctgactgctaagggatacacagtttcttttgggggagacaaaca

ttttctagaattgatcgtggtgatgactatgcagtgtgtgaagatccggt

aaactgcatcctttgcaagcatagatatgcatgctatgctgcatgccatg

tgtattgtagcttttaaagctgggtgaatgtgtatgtgtgtacatatgta

atctgtcattgtcagaaacacttgactggtggtccgtatatgccacctca

tgtattctctctctctctcccccccctctctctctctcacacacacacac

acacacacacatggatatgtaagtctgggtagatatataaacagaagctt

gtatagagaaagaggaagatagtccagtgagggatcttagggcgatctct

ggagattaaatctagggcctcatgcttgctatgcatacagcccaccactg

agctgcatccccaagctgcactggagaatttcgagtgtcggtgacttgat

ttatacgtgtggaagagcactgtagaccttgtgttgggaattgatcagag

agaatgttccaggagctagttgctgtgtgagccaccactccagcagaccc

ttagaaaaaccaagtgttctgagagttggttgggctcaactgggtggttt

tcatatagagtgccattaggtgatggctggggctggcaccttctcaacag

ggccctggggcgtgtatcatggtgatctctctgcccacacatcttactta

gaactccagcttggcctgtcggcagcagagcagcctctgcctgtttctac

agaacaccacctccggctacagaaatcagggaacgagtgataccagctcc

gttacaatcgcgaaaacaaccagagacacagtctcacctctgctctgtac

cgctgttttaaattgtcccaggtcagccccacatttagaggaatagaaat

ctagacttccctggaacattctctctgatctcactgggcgagtggtaagc

acctataggtagtaaagagaattggtggcagccatcttgcagacaagcag

ccacaggtctggagggacaaagggggactatgttgcattctggtaccgtt

ttcttattgttagaccacacacactgaggaatcccactccaggaatgctg

gccgggggtaactgggaggaattttgagccctctttgtcatgtacctttg

ctttgtccatctggactgaattcacagcagcaggaagcagccttccttct

aaaccagaggttggggaagcatcagtgtagttacgctcagccacgcccag

gggctctctgctgaccttccgtgtctttggcacatgtctgggaggtagta

acattatggccatgtaacatggtagtaacactggggacccaagggtggtt

agggaagtttacttcctctcagataccttgtggctttctgccagtagagg

ggctccatctgaggtaagtcagctcatgtggagattaagcgatagatatt

tagtagacttcgtgtggctctctcagtcaagactgagaagactaagggtg

aagggtggagaagttggagtgagagaggccaaggttgaagctgggtatag

ggtgtgcgcaaagctaagagcgtcagctacctactagtacagaacaggca

ctcggtgaggagcctaggcactcggcctccccttaatgcacggcagggct

cccttatcatgaatggtaggcacttcctgctgatccttgtagtacccctc

aggctgcccttactgtcaatgaaagaacatgtgaactcccatctaacatc

tctgttttagaaaaggggtctcaggaaccagctcagttggctgagtgctt

accagcagcatgaattcctgggttccatgcccacccaacactgcatacac

ggggcatgggggggtatatgcctgtatggaagcagatggaccaggagttc

aaagtcattttcagctatatattgaatttgagggcagcctgggggcatat

gagcccaccaccaccaccacacacacacacacacacacacacatatacac

acatgggggggggcagggacagcctaggagaggggttccaagggaatatt

gaaaataatactgaccagtacggtgatgcacagctttatcccacccagca

cttggaaggcaaaggcagatagatctctccgagttcaaggccagcctggt

ctacatagcaagctccaggaaaccttgactcagacacacacacacacaca

cacacacacacacacacacacacacacacacacactcacacagagagaga

gacagagacagagagacagaggagagagacagagacagacagacagacag

acagacagacacaggaaggaaggaaggaaggaaggaaggaaggaaggaag

gaaggaaggaaggaaggaaaaagaaaaagaaaataagactataaagccac

aggagccgggcaggggtggcgcacgcctttaatcccagcactcaggaggc

agaggcaggcggatttctgagttcaaggccagcctggtctacagagtgag

ttccaggatagacagggctatacagagaaaccctgtctcaaaaaaccaaa

aaaaaaaaaaaaaaaaaaaaaagccacaggaagtatcaaaagtatcaggc

caagtgaggtggcaggtgtcaccagagagctaacagaacagacgagcaat

ctggaggaaagcaacagagactgtggggagagaagactccgctgatgact

tgacaaactgcctcagcagaccagttttacagataagaaaatggaggcct

cgaactaggtaccgagcaggaagtgtagagctgaaacctgacccagctcc

aagttcagtgcccttctatagctcctcagcaaccggtgccatgctcctgg

gtctgtgaacctgtggcctgagcaggtcagggagagcctgtgctgccgct

gtttctctcgtggccctgtcctggccatcctcgtgacccacttaacttaa

gaattcccagtgctctgatgcccacagcttcatgtcaggataggatacaa

tttccctgattcaggttggcctgggcattccctgaccacgggctccacat

gaggagggatcagccaacacaggatagataccacacagggtaggaggtac

agtgtctggaagattattatcgagcccctgaacgtagtagaagctggctg

tcgttccagtgcaagctgagcagatggtcccatcacctctcccccttccg

actccacttagcctggatttcttcttttcctggtgtgcatgatgccctct

tccccccagatctccatgggctcaggctgccgcttcagggagaccatcct

taaggcgggggctgggctcgggctttgttgtcacatcctggctttctgtt

tgggtacagcaaagactttgaggaggataaacagagatcattttacacct

tttatattatgacagatacatactgcaaaacactgctgcttccaaaatgt

ttgggttgcaggaattaaccgctaggcagttcccaggaggctcatacttc

atttacacatcagaagttttttttttccgtttttttctttttttaccctt

ttcagtcaaaccagagtgagaagacacgtgcatgtgtgtgcacagtcata

tgcacagccaaggcatctcctgggttcagtagcaggagcgtcttaattta

gtaagtagttctttaagtccagataaactgataaataccattaatggctt

aatttatactctgggtttgaaatttgacactcggacactatcttggctcg

ggaaacagaagtggtcttgtgcccgaggttttaatgaccgatcagtaata

taaattgctacatcactgagcagaaatagcctctccttgtcatttgatct

ggaaacttctccggccaggcagcagttaataaatttaaaaataattaccc

tctttattcaaagtcaaactaattaggagtggtttttttttacaaatcat

aatgttgttcaagtcactgtatgttccctctggagacttgaacaggccta

gggacaacagactgtactggatggcggtaatgagagagcaggagtgtcct

ggctgctgggctcaagggggagtggccggggcagtctttatcatgtccca

gggagaaggcaggcactcgcagggaggaggctgggaagattcacgtgtag

gaaccttcaattccggcgtccatacagaaatctgagctactgtggtgtga

atatgggggagttagggtcaaggctggtctgtatgggaaccaggctgaga

gacaagagtcaaggagcagaaggagtgggcataccggtgcatgaccgtta

caggagactcaacacactaaaggcagatggatgggtggaatgacatctga

aaaaaaaattgcttctttgccaagtgtgggtcctagtggtttagctttat

gtcatacactatagagagaagttgcgcgcgcagccttaagtcttaggtgt

agagtatggggtaccacggcatggtggtccaggacttggtctctagtgtc

acacaatagctttgtcctttataaagtttatcatcttggttggattattc

atcttctttaaatcacattttgctcagccacggtatcaggaccatgtgat

gcttggtttgtttggggtaccacggaagcgctcagcatggaccttgcata

gagcatgcattcaggaaatggtggttattgttactgcatcatccaagtct

tattagagatgaagagcctcatccccttactaggtaggtggtaagaggaa

gggccgtgcccgtagtgatgatgataggggaactaactgttcccctatca

tccagctgctttttcaaattggaggtgtaggaacttctgggatcgtacct

atgccctccagccaagagatcgtctcctgtgtgtctgccatgaggaaata

gtagaaccttctagtctcctttgccctaaaaccaggaaacatcctttgag

caagtgtcctccagtgcccactctttaagtgtgcttgagaggagtaaggg

catcatcctaccccttcctcaaaatggaagagagaaagaggcaggcaaat

gatgagtaaggccatttctgggattcgcctccccctaatatagggagcta

ggttcaactaaaccatgaacttgccccggagaagtctttaagtctctcct

cctgctataaccttgctccagaagatctccaagttaacagtctcattttc

tgtcaatcagaagaggttgtccagctgggttgtccatcattcctttgttc

gcttgctctctgagttttagagcattatacccagggaatagcaggtgctt

tgtggctataactcataatggtgactaaaatttggttcctaccttcctga

tgaaggccatagtgctggaaagacacgagcaaggaagctgagcttggcct

taggtatggatacttgaagagatggccttgacagctctagctgtgttaac

tcctcatgactaaggtagatattatttctctctcatctttggtggagaac

actgagatcttgggggttagggaacaccagtccaagacagacctctaatg

agtggtgtcttgtggttctgtgtcccagaatcccttctttaagtggtgtc

ctcttcctgtggggcctggcattgaatctaaccagaggcttaaagcccat

gcttggaaaaggtacccagttaagaaccctccgctcaaaaagatcaaact

agaattaggtcggacttgtatgacatttcattttcagctcaagcttctgc

tcttctcccaacccccacccctgactccaaaatacagactttctttgact

ggcctagaaaacaaaatgtttagtttctatattgccatgtgacaggggaa

gccacttaaacaaactcaagtgcttagatgctacttttcatggacctaaa

actggcttcaagcaggtctctcaagccagaagccgtggacacacccggca

gtggaggctttgctgtggactctgcctttccctttcaacagggagtgggc

gttgatggacactcactcaaaggacccttcctgctttaggggaaaagcag

actagaaggttctatcacttttctcattgttatgtccaaaagaatcccta

acaagaaggaccttcatggcagggttctgactcacagtttgagggctgac

agtccttggcggtggagaaagcatggcagcaggtagctctctgggaacag

gagtgtgcagctaggactccttacttcctgacaccttagtaaaataggaa

gcagaaatacgggaaagaggtattcacctgtttttcacccttcccttctg

gcttttccagtttgaaactccagcccagagagtgtcactacccactttca

gggtggatcttccttcttgagttggtcttcttgagaaatgccatcaaaga

catacccagaggtatctcaagttatctcaaggccactcaagttgaccatt

gagattaaccatcccagaagaacacacataaccagcagctgattggctcc

tgggataaggcaaactgtgtttccagctctgatgttggcatcctgccttt

cctggcccgtgtctcacctttgatatgtcagtgacacttaaaaaaaaaaa

aagattgaggcgtgcccttgactctgatcttcaggtggcaaagagagccc

ttgtttgaagggctcctcatggaggctggctgtgggaagaatgcaggaag

gtagcaggtaaagccggaggtttaaaggggaaacactaccatgcctcctc

gggatccctgccagccagccttgccctagacctagctttagaagggcatg

ctggacacctagaagccatgtcaggcggatgggatccaggcctcaagcac

tgtctccgcctcccaaagcccaaccccagagcagacaggagagagaagag

gaggtagatgatcagaaataaccttgatctccctgatactttacatgaaa

caaaaaaaaaaaaaaaaaaaagaggaaggaccaagcctcaggctcctgcc

caaagtgtaaggtacgggggagggtaggacagtgtgagtcatcagccgga

cagacagactccgtgctggttggcaggttctcacccttgaaagcataata

gcgtttgacttttataaatatcagaggtgattcgtatagcgtttcacatg

gtgtgccaaggaatttaacacgaggaagctcaaatcccagaatggccaca

gaacgtcataggcacttggggagggggggggcggggtcatgtgctgccgc

ttgtacagtcagtgcccagcatagatttactcttgacctaatttcatgct

gttactataaaggcatgcttgatgcctaatatagagttttattttccaag

cacagatctctcggctctgcggaagctgcctgctgtagatcggggagggg

ccacccagctgtcacttgctggtctatacaaactgcctgctcccctgcca

gtccccagggtccctccagccaacttctggaggtttccctccatggtgat

ttttttttaagctggcattaatggtcatctttagagcagttttcggcttg

aagcaaaatgtcctaaaaggaatagagacttctgagagtgcccgtgtccc

ctcgcatgcagagcctccactgctgttggtgtcctacagcgccacggctc

atttgttaaccctgtggacccgaaaggactgagcatgccagtctgaggcc

cacagctgacatgcagctcagtcttggtgagcatagagcagcgtataatg

acacaggtccaccatacggatcagtttcgctacctgaaagtgcttttgtt

ccctccccccccccgccctcccgcttcatctccccaatacctttaaagag

ttattttggattatgtgcgtgtgggtattctgggggatctgacacgtatt

ctggcttcctcagacacctgtacttatatgtaaaaggcaaacgtgggtcc

tgggacctgagcttgggtctgctgcggaagcagcacatgttcttaactgc

tttgacaattctctagccccacacccaataccgttgcccccgtttttgat

tgtaaaagattgtatatgtgactctggagggggaacctgcctacggcctt

gtgcatgctaagcaagcgctctatcactgaactataccccccaaccctgg

cgctgggggctaaaactgataggcttgtgaagcaattacttctacctgac

aacctcagtatttctttttttctccttcccttttctccccctcccttggt

tccctctctcattaagcccacactggtctcaaactctctatgttagctga

ggctggccttcaacttctgatacctcctgcctgcacctcccaagtactaa

cattgccggtatacagctccattcccacactagtcacatgaagaggttac

tgccttgggcagtgtttgggcaaatcactggtctttgatggtctggagat

gggtaaaaagatgccacccaagacaggctagagacgactcatcaccttga

acatccacatcatagcagacattctgttgggaccctgaggtaggggagga

gttgtgggcaggacccacacgggcctcattgctctgcatctcctaccatg

gcaagaatgatgttggacactgctgttgacagaggaggagacagtgtggt

acttgaaagggtcaatatcacaggaaaaaggagaacaaggcttttttttt

taatcatcccatttccaccacaaatgtgctttatgacacagaaaaaaaaa

agcttcatttttttttctaactctcacatgtatagggtgggaactggaca

agaaaaattgctgtttagggactgtgtgatccagggaagaatctaagatc

tgatcttgtattgacattgttgtgaattcttctcagctagtcttcatatt

cagcaactttgtttagaaatacggaggcggcagtgaggggcgtagcccag

tttgtagagtttctggcatgcacaaagcctccctccggtccagttcccaa

cattgcataaattgtgcatagtagtgcatgcctatagtcccagcactcag

ctagtggaagcagaaagatcagaagttcaaggtcaccttctgtgatatgg

tgtttgggactagcctgggctacatgggcatatccatatatacttatata

catattatatacatatttagggatgcaagtatctgaggaagccagactat

gtgtcagatcccctggaactaaaattcaaggcagatgtggctgctgggaa

ctgaacttgtctctttcaagtaccacactgcctccccctctgctaacaat

actgtcgaacatcattcttgccatggtaggaggtgaaagacaatgaggcc

catgtgggtcctgtcctctgttcttccccctaccccagagccccaacaga

atgcctggcaggaagtggatgttcaagatgatgagtcaggaagcttgagt

cttctctaatctgtcttgcgtcaagtgtttttacatacatacatacatac

atacatacatacatacatacatacatacatacgcacgtgtgcgcacacac

acacctgttgtaaacaatacaacagtatctggaaaatggtcaggactcct

agggaacagcgtgtgtcttagtggtggtgactgcttttactctggatacg

cagcttcttttcctcaactgtagatggtgtttcttttgctttctttattc

aaagctctatagctttgcattcagtgcatactgtttccgctctaggagcc

aaaggggaagagaagtgtttgcccctctgtgaggtcttaggttgccttca

gctagtgtgcatgacggtgggggtgggggtgagggtgggtgggcagtcat

aggtcctgagaagtttaaatgatgtgactgatcatatggtagaactacat

gcaggtggcaccccaacagtggacaaaattgtactggagacttcccatgt

ctttcttgcccagagtttccattcagattggcccaccatcagcgtccctg

tggccagccttattctccctacaggtctcctctgtctctcccccattcct

ctctctgctctctctctctttccttcccagcaccctcccctctctccagg

gctgaccctgctgcctgctgacagaaaggagttagtaaaaacctatgtgt

aactgaaatctgaaaagccataaatcgccttgttttttctagaatgttgg

tagcattttgatttaatagttggacaaacacaagggcctattttattcag

agtttacaggcctcaaggctgttattcctccaaatttaatagcatcgaaa

ttgcagggttcgctataatttgcctatcgcttgctgctccgccattgatg

tttattgtcagcgagatggaatctcaatccgactcacttagataacatgt

ctgttaaacatttagataattgtacaatcattaacttgtcacattatttt

agagattcaaaacagaggcagggtaagtagaggtacccaaggggggtgga

ggtgggtggtggaggaggaggggggaacctctttggagttagataactgg

agatgagagaattgattacaagagctgctgcatcatccaagatgtctgag

ttatggaggtgtgtgctgcagagataggactatgggttttttttccttct

ttcaggttgccttcttaatcatcccacactaccatggtagtcaggctgct

gtttgctggcctctgggacaaggtgccagatgctcagaaatctccggtgc

tctgtccccaactttgtctaggaaatatgaatgtgaggtgacttgtaact

gtaaagcattatgaatgaactaaatggccatagaatctgtgaactgaggt

acctggcctggaagaatgtctgtgatgttttgagggcttttttaaaaagc

cgattttaaagtaaattttaaagaggacctggggggaaggatggttcagt

gtgtaacgtgcatgctgtaggagcatgagagagccccgagttcggggatt

ctaaacactcacatatcaacctgggttcatacaaacagaagccagcctgt

aatagtctgcgatgctctattgatattaacagaaactatttttagaaaga

ggattagagggtactttacttcctgtttttctatgttttagtaattattg

tgattaaacatttgccacttggataacgctaaaaccctaatgtcacttta

taaattaggaaaccttcatggttacatctcattcctcccatggagggaga

gcgatcaaggatattcactgccctgtcaccttgacaggtgcctcagttcc

tctactgtgaagacaggcaggtgccaaacggggaccccagcaacctggca

taggcctgtgccagtctctggaggtcagggactatgggaacaatcacagt

aacaatagtaggctggtgagagctaggaatgtagacgtgctcctcagaaa

tgagcagtatacacattcttgtctgagagggtcccggtacaggggagatg

cgactgtaggtctgtcttgctttggttagacagttatgccctgcatcccc

agagtatacactgtgactgggacaagagcagcactgccatttccagctta

cgtgtccccagatgacatagaaaagttggggaaaacacaagtttagcaaa

ataaaacaaaaacaaaacaacccccccagcttagtatttccccaaccata

ttgtagataatgcaggtactggggggtaagaggataaacaatataaatat

cttataggttgtaagctaaataatgcatatatttagaagaacaacgtaaa

tatttcataggttgtagtatagattcttgccaaaatgatagaactggctg

ctgaggccagcgggttgagggtgagggtctatgaggccagaatggagggc

agcagggtgagataaaagggagcccggcacttggtagaagaagttagcca

actgtcactagccaggaacccaagcatgccgggagctttgatttctcctc

aacacataataccattcgtttctaatgtggaagagaagccaatgatggaa

taaaacaataatgaggacttcttttctaagatttacgttgtgtgtgttta

tgtgtgaatgccacatgtatgctgtgttcctgtggaaccccaaagaggtc

agtggaactgctggaccgagctcctaacacagggactacaaccagactct

gggcttccagaagagcaaacatactcttaactgctgagccatccctccca

tctctaagaatttattctcaaaaaagcaatttcagccaaagagaattgaa

aagcatgcactcattcaaacacttacgagagattcttcatagcagccgcc

gccatgtggtccagaggtgggaactacccaactcttcatcaactgatgaa

tgaataaatacattatggtatatccacaaagtagaatattattcacccat

aaaaagtgaaacactcatacatcctatgcaaaagaggtctaaatgcaaga

atgcagaaggaaaaggctttatgttgtgagatccaatttatttgaactgt

gcagaatagggaaggtggcagagagaataggttactgatggccggggggt

ggagaggaggcagagaggtggtttattactaaatggctgtgagttttctt

tgagaggggatgggcacactctggaattagtgatggtgatggcttttggc

tttgctgaacttattaagaaccattaatagggcaaactttttacatctca

actgagggcaagtggttttaagtttgtaaatcacataagaactaaagtaa

aagtaattttcaaaagtgagtgtgtgtgtgtgtgtgtgtgtgtgtgtgtg

tgtgtgtgtgtgtgtgtgatttttttaaaacagacagcttaatctcacat

ccctgatgtatcacataagtgtctgcacaggtagcactttgcattcagtg

acccttaggaaaaaccggaggctgtggccattctatttgggagatcaaga

tggtttagaggtgcctctttgctgcctccattcctctttcttcaaggata

agaaggaagacatgcctttgtactggtacaatgtccacaaagtgagaaga

ctgggcagttgtgtgttgggcggatctggagcagaaaagccagcccacag

ctggccttggaggagtcatttataaaggataggacaggacaggattggct

cagcagcttatacagagaatgagtcccatcgatgatgggagaaggtttag

tgtgggagaaatcagaaaacaaaggaatatgccccgagtccctcatgcaa

gcatgctgttttgtatccaacctgtggcttgatggccactttaatggctt

tcctgtattcaccctggccgttcattctcttctgtaagcccagtgagcct

tacatgggcagtgtggaccagattcccctgtaaatgctgtttctggaatg

gtggaagcagtaggcagcagacccggctattacctcttacctatctctgc

tgctgtcaacaagacaaagctcgggaagccagacagtgctgcggccgctg

cacgggccgttccaggcaggctcaggatgctgggaaggaacgcatctcat

cctcaccttcaatcttccttctagctgtttccttccgttttcttcagcct

tgcaaatggccactggccctgggcagggtttaggcaagtaatttatgtct

tctcctcacttaacaaatgaccctgcctcaaggttgggcaaacagaggaa

aaatccaagcatcaatttatttttgccaggctaaaatctgtagttaaata

gcaaaattaggcatctccatacatactcaagtacagctaggggagatttt

atagagctgatgaactccaggcaggtggtaaggggcggcggggtgcgggg

tgcggggtgcggggtgtgtgtgtctcgaaaggcttttgttttagaatgtt

ttcttgtgagcattaggaaaagttacctcacaaagagattgcgggtttgg

gtcagatgaaatgtttcctgcccctaccaggagaagatccattacagatt

ggaaatgactttgtctctaagagcccgttgtcagatgggctatgccttcc

cttcagccaaacaaatataaaatgcttccagcctctgcctcgattttcta

ttactgtttcacgggactccagagagttttaagatagcaatttgattaag

cttacggggaagattctatcagataacattcttcttccgcaggtggaggg

aatgcgtttgagtattaagtatgttttaaaggtaattatcttttccatat

tcattttagttacaggaattgaaaagggggagacctcgcgtctgtaagtt

gccccttgggccccagagtctctgtgacttctctctggggagcacagtga

aatgtttttgacaaatgaataggcaccgagttgttcggaatttgaaatga

ctcagagggtccctgcctccagagcgctggtcttaaaggtaagggaccct

gtcttacctcccagcttacattccttgttctggagcacccctggatgtct

agagggggcttggctggtcctcagaaagacaactagtctcattcaaggac

atgatggtgctgccggcctgaaattagcatgtatgtatacctgaagttaa

tatgtgtatacacctgaaattaatatgtcccaactttagagtgtactcaa

ctgagagatgtcagaggtcctaggagtcaaaaatctatatatgcctaaat

catagaatgggagcatgggatcttagagaatgtgcctctgtgcctcacag

atattctgtgatttttttcccatgatgccacagcaagtcatacagacata

ctctgtgattaggacagaaacccctggtcttagagcattcctactgtctc

atagtatgtctacaactttgtataaccacaaacaaacaaacagtcctttc

cttcatttccttcaatgtccagagcacgaaattctgtcctgggtgctatc

tcggttagctttttgccaacctgtcacaagttgagtcatttgagaagagt

gtgcctccattgagaaaatgtcctccatcaatctggcgtgtgggcaggcc

tgaggggcattttgttggttaaatgattgatgtgggagtgcccagctcac

tgtgggtgaagccagccttcggctgatgatccagatttgtataagaaggc

aagtggagcaagccaggaagagcaaagcagtaagaaggatccctccatga

cctctgtatcaggtcctacccaggcttgacttcctttgatgatgcactgt

gatgtgatgccaaagtgtaaacccttttcttccccaagctgtttctaatt

gcggggtttggcaacagcagtagaaactctaaggcatcttccaaagtgct

tttggagaatgaggtcttcatggagaacctcgagagcctgcaggaaccca

caagcttggtattgacattgtgcagttcaatgacaccttcgctgcccatt

tgaagtccttctaaatattgtgcatttcttcaacatgggaaagcacaagt

cttatattccccatgatgcttccctggtacatggtctactgcttattggt

ctgaagcttaaaaggaatggacccaggttagacggttaaccaggtatact

cccatctggtacactagcttgcacagtgacctccgaatgcctgcactatt

tatgtgagaggttcttggcagtggaggagcagaagttaggttggtgggtg

tatggcatcacaggctgacttctggatataaggaatgttgacatccatgg

tgtatgtacgtctgtccttcaaagcctccctgctctccctcccccgggtc

ttatctctagtacctgttagaatcttggtgatgtagaaggcacacatacc

cagagatgcattgtaaatacattgatggctgccgctgccatccgtggtca

tgataaaaagagtcttgtgggggaactatgtgagaaaatgtaacaagcaa

agacttagacatctctccagtgaaattaagatcttcagagatcctgagag

gattcatatgtgccactagagttcagaactcctgagagggataaatcaga

cttcattgtgtggcctgaagcttaaaaggaatggacccaggttagacggt

taaccacttatactccatcaagtgtcccctgctccccctttattcctgga

cctaattgtcatcttgtaatgcacctacacagactactttctcctgtttc

tgccctgtgagttgtggatgatatctggaccattggcatcacagctcttt

ggctatcttattgcaggtagtctctcaacagcgatttgctgtgtggttag

ccaaaggcagtgaatcatgctttctccttagaaggctcttctgtctggat

tcagatttctgcttccttctcgactgctgcaaggctttggataagtcacc

cggccttcacaaatctcaggatcttttaactgagaaattaggacacaaga

gtcaggggaagactggtcagtcaggacagtgtttgctatgcaagcctggg

gacctaaatgggattctcagcaccccagtcaaatccagtgacatcactgg

ggaggcgggagaattccttggacttggtggacagcttctccagctgaatg

gatgagttctgtgggactttatctcaagaaataaggtagagagcaattaa

ggaagatacctccatgttgacttccgacctcctcatgcatgtacacatgc

atacgtatgtccctacggtagcacacatgagaagcatgagcacacacaca

cacacaccatacatacacaaataggataccatacttggagcacatatact

aaacttaaaaaacgagacaggaggagcagaattaccccccagaacaaaat

atactgtctgtaagctcaggtgtgaagtggcaatcacagggtcctcaggg

gcctgggctctgtgtgctctctgaggctgccgctctccatgtgccctccc

atcccaaccctctggtcttttttgttccccctgtggctggcttcctgcac

ctgccacgctctgcctctgtgcataggaggaatgcagaacaaaggccaac

ccaaccactattatgctctgaatggtgaagaaaataatgagagaagtaga

ctgagccatttaggactgaagaacccagtgtcaggtggtggacagaagtt

aggaggacatgtttcctaggattgtgaccctttgtcatctgaacaggcgc

atgacacagacccctcataatctggcaggttgagtcctaggaaaaaagag

atcgctcacatctgccagctcttctctatcagacgtagtttgagtgccaa

acactggcacctggctggccttccaggagctggcaaatatcctgaaaggc

tggaccagagatgctgcccaaggtaagtgcagaaagagtgaacacctaac

cctgatggcccgggcaggcagggctgataacaggctctgctcacactgaa

tttccccccttaccctgcaccgcctgcttaggacaaacatattttactgg

ccatcacttggggtttggacctttgtgtgtatgcctgtgtgttctcaggc

ttttactaatccccttccttggtttttgaggttaggagcgtctgggattt

gtggagggaggaagggtatgaaagggtaaggcaggagaccctggtgcctg

gattcaacctcccacattccggagatccctagaggcccctctccccagac

ccttcagctccgaaaagccattgtgctcccttctggcctctgaccccgag

gctctgggaagcagcaggttgttgacacacggacaatcatttcttgcaaa

gcacaggcctccctctctccttccctccctatctcagtgctcacgagctc

gctctctctctctctttctctctctctctctctctctgccttgccctggt

gactttttcacagccaggaaggcaagagaagctgagcaagagggaaatta

agtctctatgagagaggaaaagaagaagggaagaccctcccaattttggg

tcccccaaacatcttagttgtctgagggaccctcctccggtagtctacta

ctagtgcagaagtgtgagtccccctcccccctgtacagccacgtggatcc

cagtagtatgtgatgctaccaggaagtggctcaggaggagcgcttctttt

gagagtctcacttatgtgaggagccctgcatttcttcatgaagaatctct

ctgtgcctgtcctctgtagtcacgggaacaaagagcttcctagccctgtt

gctgctgcttgtgaggaggagtaagacttcagatgtcatttgccctgtca

gagggttagtttgctctttggcagaagtgtgtggaataagctacttgcct

catgggcctactgtgcagtgcacatagaggtttctgtcagggagtgcttg

acaagcttcagagcgctcaacagatgcacgtgtgggttactgatggttcc

ccatacagctctggctagtagtttgtattattctcatgggcaggatggga

gttccaagctttttgttcctcctgttctgtctaggccatttcaaaagcat

ttttaggagaacaggtggggtgggaaagagagtcctttaaaacttcaagc

accactgtgtatgcttttcgccttggttggctagccgggaaaccagttag

cccgaataaactcaagtgtgtgtctctggccactaggtagccccattcag

cagactgggtttctccaggaaagaaaaacagaatggtttgttaactacag

aaatatctctcccttgtgctctcattctgaaagaagtggattagtgtggc

catatttaacccctgaccccgagacaaaggcattatcaaaattagttact

gagagagtccgtcttcccctctctgcccccacttccctgtttgcaggggc

ttcactaggagctcggtaaacttcagagtctgagcaaaggtttcggaggc

catggttgccttggagatattctaaagcccgggctgtggagatagtctaa

tatggactgaaggatagagccgggatctgtactcatgtgtgggctctttc

tggcttcagatgttccattggattttagaaccgtattttcccagttttta

actatatgctcaactgtcacttgagcctcagatcttgagttgatcttaag

ggtggcatccagtgtctttctccgtccatccctctgtccgtcctggagtc

cagtccctttggaggtggagatgcagctagacctaactgattctcattct

ctttgttattgagtgtcttcatagcctgggctaactcaactctagactgt

ctttctatctgggacaacacaatgacaataagcatccccttgagctacgt

ctgaggttagatgaccatgggacagttgtcaggggttagtgagtattatc

tgtcatggtcagtattgtccttgtcatcagcatcctagccacagcttcct

catcacctccttggtccccatcctgtcctcctgggataatccaggccaca

ctggggttttctgcctggacttcttgtttcctgactctgctttctccctg

cacctttttttttctggctgtttacagacaccccgcctagccttcctgga

tggagtttaaagtttgactttctttcattccaggaagtccaacttgcctg

gcaggatcgggagggaaaggcactttctttataagctctcgatgaaagct

gtaaagcaagccggtcaagggtctggccggccctggctcaggattaaggc

cagccctggaatgctgagggggcctctctcaagaagctagtatctattta

cagtgcatgtctcataaacccgaaagccagggtgagggcagggggactcg

ccctcctggcatgtgattgattatctggtatttatgccattgaggacttg

tcagcggtctggggtcacagggtcatctcgtgtgtcttgccgccatcccg

ccctcttttgtacagtgtgggccagggtgcactgggcacaaaacagcttt

ttcttttccttgggaaaagggggaggggggacccggggcagtgtcaatga

aaaaatgttttcaacctttctcaatagtgctcttttaattctaaagaaca

tttaattaaaagtagcaggaaaattcatcttagaaggtttaacttacctt

aacagatactattgtgaccaaaaaaaaaaaaaaaaaaaaagtaaagaaac

gaagtaaaaggttttgtatctcctgagtcctccgaggccacttttgcttg

ccctgtggtagtgtggtcctgacttaggaaggagagagagactgggttat

tgctaagcagggttacgttggtgtttgttgtctgtacagctttttcttgt

ttaggatggaggctccaagctttggatggaggctccgagttggatatgtg

agcatagtcccctagaggtggcagcctggccctggggtcccctcatgatc

ccctttcccagggctttttcagcactcagagcttgggacacctggagaaa

actactacatcagtgccagtgttttgccgttcaggtatctcaatcctgga

aaccaccctcttgtccgttgtggtagagggggcatctctctagggaagct

actgcctctgggacgacagtgtgtgctggggctcagaggatctcctcttt

ctaaaatacttggagagagcagggcttggtttcccacctttgtaggagga

acaagtccccgccccagcccatgatcagcagagaaatctgcctgctcact

gacgaccttccgtctgctgaagttcaggaatggacactatttgtccctat

ccccatagaggatcttctggagctggggcaagccagaacactagtatgga

gtgctgccccacagtggtcttgcggtctgtgaagggggtgtcagcttcag

agacccacccaggctcccgtctgtgggacacccagggtggggaagcatga

ctccctcctgggacgttctgtctgccacgctcagtttcctggatcaccct

gcaaggctgggcaggggattatgtgcacagagttggctatgtgtttgctg

taaagccccatgtccccatagcacaggggtttgtctccctgtcccaccca

cgcaccatcctccacaacaaagagtcgttgtctacagaatagctttagaa

atcccaattcttttctatttccttcattcccattcaagttaaattagtga

atgctaatttgggttgatgggacacactgggtggaagctgaggttctggc

tcctgctcagggaaggaccatcgtggcagcatcctcaccagggtcaccca

gtgcccggtgccccatgctctgggaatgcttggtttgggttactttctta

gtctcagatcagctgcgcaaggtgaacattgttgtgtctaaaagacaaaa

actcagaccgagcacaagtagacttccctgacgttatggctaatgaccag

cacggttaattctgaaatttgctttaagctattcggtaacaaacatttcc

ctcttccctgaagctgcttagcatccccgtaagacagttactgtcgttgc

atttttctcagagatgaggcacagaggagtcaggcaacttcctcctggcc

acacagcagcagcaagggttagaacaagggtgtggatctgattcatagtg

agtgacacaaacaagaccggaacccagagccttctgccgccagggctcgg

tttaggtgtgtttttcctcactatactgacatccattactgggagacatt

atacagagacatacataaatcagccagagactggcaaaaggtcacggtgt

aatcagacgccatcctgggtggtgtatggagaggaatctggaggaagatt

agggattcaggcttctctctaggctctcatagtaggaggacagcagcttc

ctttctagtaactttcccattatactcctctgtattctcattctctctct

ctctctctctctctctctctctctctctctctctctctctctgtgtgtgt

gtgtgtgtgtgtgtgtacacacatgtgcacatgagaaggtcagaagacag

tgtgccctagctaactttctttttcagcatgtaggttctagggatcgaat

gcagctctccagacttcacgacaagaaccatcactcactgagcccaagag

aaatgtcttatttgtgccccatctctggatcatctcagagttgataacat

taaataaatgctaattaaatgactaagaaagattgaatttggggcctgac

ctcactgtttcatgacagtactattgatggctttcttcctctcaagtgta

tgttcactgcatcctaggggctctttcaggatccctatgggtcttgatat

cttcaagggaaactgaggctaaatcttactaaaggagtcaaagcctttgg

acttaccaaaaacgtattattttttttattacaaaaaataaaagtaggct

agggaaatgtctcagcaggtaaaggattcaccaccaagcctaaggcccat

gtggcagacagacagactgactcctgtaagttgttctttagatgcatgga

acctgcacagctacacacgcgcgcgtgcacacacacacacacacacacac

acacacacacacacacacacacacacacacacacacagagagagagagag

agagagagagagaataaacaataaaaacaaaacactaaaacaatgaaaaa

tacatatttttctgattacaacaaccttcccttttcctggaatgacagaa

ggcaggagcggagggacaaatgcagaagtcagcgaggacagcagagccac

tgtgggcacagagcgtacacattgactgcatcatctggtcgttcatgctc

ctagctgggagttgcctctttttatataaggatttctggatctctgatgt

taaatggtagccagttgattgaaatgataattcagaccccgaatgctaaa

aagaatagtcttttatgacccttaatgagtgctataaaaataagaggtga

atctaattttatgaacgatcctttatgacttacctgataactagtacttg

ttcagcacaaagaacacataactcactcaggccctttgctggctgcctgc

agccaaatgacctgtgttaggagtccccatggcactgaacttgaaggatg

cggacttgggtggtcccaacacagctctctctggtggatcataagactgg

ggttcctcattttgggaaagtggtggtattactctcattaatgaagcaga

atacttaataaaagtaatttaaagaaggaagagttcttttggttcaagct

ttaggagggagcatagtgcatccgagcgaggaagacatgtcagcagtcat

gaagcagggaggtcagtgttggcgcatacctgagtccctcctgtttcctt

ttctcgtcttttcctgtggatggcatcatcacttcacttcacggttaact

ctcccctaaaacaccaaaaaatacgtgatcagctgtgtctcctaggtgat

tctaaacctagtcaagttgacatggagattgagcatgtttgggaagtgaa

gaacgatccagggtgaggtggggtagggttagagagatggctcagcagtt

aagagcactgactgctcttccagaggtcctgagttcaaatcccagctcat

gaccatctgtaatgggatccgatgcctcttctggtgtgtctgaagacagc

tacagtgtactcatacacataagtgaatcattcttgagaggagggagaga

gggagggagagagggagggagagagggaggaagatccacttggcctgaca

gcacaatatgaccagggctcagaatcgtttccaactaagaaggggttgaa

agggggaaaataccagcagattctcagacatttagtggatggtgttgatt

ctacacataggttgtctgttatgctaagcaagagactaggaatggagaga

atggctccctttatctttgtctaacaatgagaggaaagggaggaggagga

ggagagagaatgggagcagcagcttagtgagatattggtgaggatttgag

gagtcgcagaggtgtccatgtttgtgttatgtgtgggcacatgtatgcct

atgagcccctgtctgctttgaaagctccatttggattactttatataaag

tttatttttagattatgtgtataggtgtgtggatttgtgcatgggagcgc

agagcctcaggaggcaaacacagggtgttcgagtcccccaaactggagtt

atgggtgactgtaaatgcttagtgtgggtggcaggaagtgaatctagtcc

tctgagcttttaaccactcagctgtctctctagacccatggtcacaagtc

taaaataggatatgttggttggctctagagttctagccttgtccatgaaa

gccattacataaccagaggccggggcttggatcctctgggtccatcgtgc

tgacttggtagcagataggctttggatagagggtgtccaggtttgcaatc

tggaagtggtgaaggaaggagaagataaagctgtggtcaccatttgtctc

ctttcttcactttgagaacaggccctgtggtctgagtgaacctctcccca

ggctgttgagctgtgaacacaacacagctttaagagctctaaccccccct

cttcctgacgctcactgccccttatacacaaaaggtccaatgaaggttgc

tgggtagggcaaatgcacctgtcttaggtgtttccattgctgtgaagaga

caccatgatcagggcaactcttataaaggacagtgtttaattgtgactgg

cttacagttcagaggtcaggtccattatcatcacggtgggaagcatggta

gcatgaagacagacttgatgctgtcctccagcatcaagaactgagagttc

tacatcttgatccccaggcagcagaaggggattagaggtgtgacttcaaa

gccccacctccacagagatacacttcctttaacaagccactcctccaacc

agccacacctcctcatgatgccactccccatggaccaagcattcaaacac

atgagtctatgggggggcatctgtactcaagcccccacagcccccctccc

catccggatgcttttgcagtcctgacttagctgcggggggtcttgtctct

gctgttatgttgggtctgctttgccctttctccttccttcctaggcagtg

aactgctgttggggtcagaatgaaactgggtgacaaggagagggacgact

gctgctgagtcttggcatgtggagtctgataagctttgctcaccctactg

gctaggggcctgaagaaatcatctgtcaaaacttttagccatgagcccaa

aacacccataggtgaggtggggagaggcagcctgagccttgtacatagtt

cacttgcatgttgcaaggctagagaccatgggaaatgagaaattagccac

cccacacacacacacttttcaatgagttggtgtgtcaccacatgagcccc

tgttgccaagcatttttagaagacccgggcctaccattgcttatctgttc

taagcttatggggagtgaatgcagaactggtgactccacccggtgtcctg

atacagagctcttggctgtgggggtgacagacatgataggtcacattcat

accaaatgtcctgagggagacagagatgcacagaagtggggggggggtag

aggaagggtgcagacacccaggggacgggtaaggctccctgaaaaggaga

gaccatgaggttataaagaaacagcaggaattggctgggatcaacggagc

aagaggcagaccacatggaatcgtgtgtatttgaaaactgttgctgagac

cacatgtggctggcggagacaggggcagaccaaatcctagcccttcttta

ccatgttctctttaccccagctccccccacactgtgctgaggagtccggg

gtggcatgggctgattctggagaggtgtgcaggtggcttgggatggattg

gttggtgtctgtagggagtgaggagggagacagagaagtgctagatgact

ggatgaggagggtaggaagaacacagaggcgtcaacacagtgtctggtgc

gggcgacagagcggctgttagcgctggcagcccaggtgacgatcaagaga

caaaggtggagctctcgacagtgaataggttgtgtgcagatgctggagca

tccacagagcacttgggtctgcagcttttgctctccagaaaagtgtagtt

actagcttcctagtgttcacggagaccgttaagagttcagttctcccgag

acagatgtggggatgagatttacagatgaaagagcaaacccagtgtgcgt

gagcattgggttgaaggacagtgctcagcagagatgcagtgctttgggaa

ggtgagcaggtttttctggctggagggtcctgcagaagtgaactggtgct

ggaatccccagagccaggctagatgggctctcagagtgagggggggttta

cctggtcctactagagagcagagaccagaaagctgagctgtggaaccact

ggggcctctagagatggcaagtccgttcttctgacgcccagggattggat

gctctgtccttcatgctgtcgatggccagttctcagagaaggacctaccc

tattgttctcgccttcctaggcagtctagtccagtgcagtgcctcccgcc

ggttcctgggcctcatccctctacacgtgtgcctctgttctcagatgtgc

tatgcttggctccgtctcatggtggggaggggcggggaggggcagggtta

aaactcagtttccttccaaaggaagcagagaaatgggctcctcatacggt

ccttatgttcttaggtcctcacaatcctacaccaggtcccgacatgagca

cttcatttgggatgtgtgtcctgcctcctctccagacccttccctcagtg

gtgtggcacttggggaccagctgccagcatgattgacacaaagccaatct

ataaataaagtctttttcttttctcataaagactttgtggttgagtttgc

cttcaaggtcaagatcagacctttctaaataaacacatatcaaaggatca

ttagcaaagcagcactgttaaattgagatattaacttctgtcaactgctt

gcttcttttttgttctgtaactcatgactgcagtaggtttttattatttt

gtccttaggcggtagcatctggcttctgtagagactgtaaaaattaaaaa

gcaacacaaatcattgcgagagaaaaagcagtaaattcaatgataagaat

ttaaaattaaaatgttgcaaagcttttatattctgcaaaacatgaaacga

atgagtctgttgcctgttgccataaacccggcccagctaatcgtctgctc

cagttttttccagtctcctttgctcatcaccatcaattctgtcttggcac

gaggcgagacataaaggggagagctctgtgtatatgcgtgtatgcatatg

tttgcatgcgtttttttgcatttgcatgtatatgtgtggatatgcacagc

catgtatgtatttgtgcatgtgtatgtttctgtatgtatgtattcatgtg

tgtgcatgcaattgagaagctgcatcttgtggctctcccagacagaagat

taaatatttgtattgaagtcattcattcttattgcaaaatagatttgtga

ttctgaacctagctgtgttgtaggcgataagggagattaaaagtatttat

tatcatccctgctctggtggaactagctataatatatgtgtgtgtgtgtg

tgttctatatacatgaatattatgtattatatcatactacatatataaca

tgtatattacatatatgtaatatgttatatataatatatgcatatataca

tgtatattacatattgtaagggagattgaaagataacatgtatatattat

atatgagatatatctgatatagactcacatcatacacacacacacacaca

cacacacacacacacacacacacactcgagataatgtgggaccggagaat

aattaagattacctactgcatttgcagagaacctgaaatcagtggaatca

gtttccagtacccgtatctgtagttcataatcacttatagccagctttgg

aggaatccaccaccctcttctggcctccatgagcacacatgtatgcatgc

taaaacatacacacacacacatacacataaataattaaataaatatttca

attaaataggtagaagatagtttggggtgactcttcataaggttaacttg

atgtcaccaagctgtatacaatactgccaggagccagtggtggcttccct

cagaacctgggttgctttgagctaattccacagacccatctggctctggt

gtgtttggagtgagcatttgttgagatgagtttactaccccccccccccg

tctgtcctaataggactgtggggatggcagtgtgaattggggaccattca

ggtcagttggctggtctgactcctggaccgcaggcatgaggctagagatc

tttgtccatgatgaattctctcccggggagactggaggaagcgctaggcc

ttggcctccatcaaagggaatgaaggaaagtgtgaagattaggggacaga

gaggtggagtgaagagcatatgactaactcgaggtcacagaatacattgt

tctctgggcccagatggtccttggaataaatcagcccgggcagaagcaga

tttccactatgagtaagttgcacagtgacacaacccaggcccctggccct

gaatggcagccattcatttctctgctgagggtgtcaggaagagggtgtgt

gtgtgcacgtgcatgcgtatggtgcatataaacatgttcttatagcctct

gattctcacaggcaagtgtgggtgtttatggggtgagggctatagggagg

tttataagtcggagcccaatactgagagctttatacgtagccgtttgttt

ccctttatctcagtcaggggaaagagaaccctctgaaggcagggcccgca

ttggtctcctataacagcgtgtcccaggcctcacacggtacagggacagg

tcacaggaggtggcagaggagtgaacatgggcaaagcttgggcacaggtg

tttgtctctcgctcctccagcaccccatgttcacacacgtgaataagtgg

acaataattgtgtgctatttctcatatggctcccaccttaaggcgctgtt

gcagactgggtgaggtagagtaggagccctcatcggagccgtcacacagg

taggcatcattgtcacgcacggttcccagtagccatcacgatggcccctg

cttggagacttgcaggcatatgcaggacacaagctgagtgtgggcattag

agtgccatgtgagggcgttgctagcacaggaagcgagaggcaaagaggga

ggcttctgcctcgccttgtttgcctccaaaggctgcggcatttctttgcg

tttgcaaaagcctgtgaagacttcttaggagtgaggataggtcacggcta

agcggtgagaacaccgagtaaataccaatgatcaaacgattctccctcga

gaggcctttcttcccatgaagctgttaattaatataaatgtgctattctt

acattctaaggcatgccttctgcatagactgtatacagctgagaatcccg

ctcttcctaagctgtgttcttggacgtctccgtccgttgcagtgaagctg

ctttggagagtcaagacagcttctagctcctgcctgtttagagggctaag

taattggctttgtcctaaagtgtacagctaagtgcacagtgggaggagta

gatgaagacatccctggctgacttgcagttgagctagattttacccatac

cccatgggcacacacacccctccacatcttcatctcttaccttcttccac

tccattctggtttcaccagcctccgggtatttcagaagacctcagactga

ggttgctaccatgtctagtggggtgttatttttctcccattcatatctaa

agccttggggaatcctctgggctgtctcatgaatgttttcttcaccagac

ctctcagagaaagcttgatggtcattaacagtctctgacagctggattta

ccagagaacccctgcgtcctctcttcaggccattgtgcagttagcaatgg

cgactgaatggcacagcggctgaagcatgaaagaggacggagctgggtcc

cttatgtagcccaggaatgtttggaagtggaagtgccaaggagtgatagg

tggaatgccaaggagtggtggccatgactcacagtttgactcttgtgcta

aacactttgtgtctttgtggccaaatacccgattgaacaatttaagggaa

aggagagatttatttgggctcctggttggaaaagttcagcctttggtcac

tttgccccctgtgttgggacagagtgtcatggaggtgacagtgttcagtg

gaggctaatcttaacttctagatggatggaaacagagagaagtgcgaggc

agcgctctcaccagctgcctccttctcctcttttactctgtcagtgtcgt

cagcccatgtgacacctattgtcagagtggatttttccccactgtgttaa

tctttgctgaaaacaccctcggagtgagtctgaacagtctcccgggtgac

tctaaatacaatcaagatgacaatgaaggctgcccactaccactcggaca

ttgctttcatactttcatgcaatatacttcatgagatcgagcagccctgg

gtgagagctgtgtggacagattatccatgcaggcagctttgacttttccc

atgaatatggggactggaaaatcattggacaacccccacctaggcttgat

gagtctatgctataatgtcttgttgtagcaagatgatacaaatcaagtgc

ataagagatgcacacttaatatacgggcccagaaacttgtaacttagagg

gaatcaggatgggtagctggagagggcaagaagtcatgtttgcttttgag

gatagtaagaatcaaacgttaatggtttggcctctggagatcataactag

caactctggcccatgctcacagagaggtagtactagttcgtttgttcatt

cagccatttgtctgttaatccactcagtaaatactgagtacccaagatgt

agtgtgtagtctattaggttctgggaatttagtgacatgaaaatcaagag

tccctgtcttcatgagtctcatgccttctatgaaaacagataagcagtta

ggcaaactatgtaaatgctctcacattgtgatgttgaatgtcaaagaata

tgttagagcctctccccccacacacacacatactgcttgtgccctactta

tggataaggctttgaggagggggtgtctcctgggtgtggtctcatctaaa

gaatgagagggtgtttagtttggacagttgggtagtgggagaaaggggaa

agtggtaggctaagagccaatgtggacagatgtccaagtgtccaagggtg

ggagagctagggtgaggacataggctgaaaatggctgtggagagacagct

gagattcacaatttgagacaaaaactgagactccatgctaaaaacctccc

caaaatgactgtaagtatgctgactgctttctggggacagtagggaacta

ttatgaatcataatcaagaataagacattaagttatggccatgtgtgagt

agattggagggcttttgccatgggtgctgacattctgtgggatgagattg

ccatcatctagaacaaaacaagatagccaggcatggtcgggttaagtttc

aacactcaggaggtagaggcaggtggatctctgtgagttttaggccagct

tggtctacagaagacaggacagccaggactgttacacagagaaactctgt

cttagaaaaagaaaatctcaaatccatacaattgcagcagggatggacag

gatggacaggatggaccagaggacactagagagctggggacatttggctg

atactctgtgagaggtgagaggcaaccttagacaatgcctggttttattt

tttttgcatcactaaatgatggaggagttggaagttgagatggggtacaa

gggatttgtcactaagatgatgaagggtttttgatattatgagaacaaga

tttgagccacttagaggtggaatggagttgacaaaagattgaggtggcta

ttctggactcatcagaatgattgtctagagccctggaagtagaagtttta

atactacaggaccctacacatcagcacctccaaggcaggcaggcaagact

catccctgaggaggagtcaggggatgagacacaagtgagtagaaatacgc

actgtaagatgtgctgttgttggtaatgaagaagttgccagttttaagta

ctgttgagatgtctcctaagaggaagatgaaaaatgtctggtggatttca

tggcaaggtgacgactggttatcagagatgagaggtttgggtgttggcaa

aataatggccacaggaatgtagtggcagtggactgaggggtacaggggca

ccctggaggtggaggtaccatgtgtggatggctctaggttacagaaggtt

aagccgatagaagtcaagatggtgaggtgaccacggcagaaggaatggac

agggaagctgaaggattggccagcagtcagaggatgcccgaactacatgg

accaggcaagaaaccagggaacaaaggagagatactggaaggtcagagag

ccattctggtgggaggaaatggactggagaacttgaaaggtgtgtcagta

gaccagagttgaacaaaggagaagctgcccttttcatgatgcaaagcctg

ggctgtccctttaaatgcctcctggggcgccctgatggattcaatggaat

agactcagtctctttgtgcatccctagcagtgtttgataaaaagcacaaa

cttctttcctcatggttcgggagtctggacaagcccacactgaagtgcca

acatcgggtgagagccttccacagggttatcccatggcacaagaccagag

gacgaaaagaaggcagtaaggatgttgagcagtgcagaggaagtaaaagc

aggacagactcccttacaaaggacagactcccttacaaaggaactccact

caacctttaggccccacctcctaaggcgacactgtcagttggagaggggc

gggaacattcggatttaaatataccacccttatgcaacctgcaagatctg

tggtctaaacagctactatttctcaccggttgctatatgtcatcacagta

gacactttcagatgtgatctaatccagcaaccttgtctgatgtcactaca

tacggttgtaaaggtaggaaatggatggcggaggtggaagctgagcctgg

ggtttggtctgcctggggagcataggctggctgttgctctgttctcggcc

cacaggcttcttagggacatgatgtcttactgtcattggcctcagagcgt

gacatttgcactgcctgtatttttgttttatttgacataaggtcttccta

cgtaatccaggctgggctagagctcactggacatagtcttgcttgtgcgg

atcctcctgcctcagccctcttcctgctgggattccaggtgtgcatcagc

acatattgcccgatgactgtcattcttttccggtgttaggatctgaggct

aggttcacctccagaactttctctacagcatcctgggtgtgagaaaatta

actgggttagtcttttgtacaatatccagtaacgctacattgatcattcg

ttgggagtaagtgatgccccgtgtataagagaagtggaccccactctcaa

ggagcaatggttctgacagggagggggacacaggaagccataaacttgaa

ggactcatggagtcttggttgagaggcagacttagcccttacccagtgtc

attaaggaccctcaggctgtagctgtccttgtggctagactgagcatctc

ttctgttcttggtggcaggtgaggaagtttaaagaaaggaaccccaattt

ttggtttgaataacttttgagagtagagcagttgaagagtatggggaact

gtgaaccagcctagaccatggattttaattagggttcttttaatttggat

ggttccgtaatgcttgaagcagttttaaaagtacactttaatgaatcttc

attgtttcttagtcataaccaacacccatctccaggacctggtcatcctg

tgacgctgagactttatacattaagctccacacccacacccgctctctcc

agcctcctgagcaatggtgtgctccctgcctcccgccacaatcccaggat

tcctcagagaggtgaagcctctacacacatgctcagagtgatgtctgaga

agaggggatgtggcagaatagtcgagagctggcctaacacacacacacac

acacacacacacacacacacacacacacacacacacacacacacacacac

acggctcagtccccagtactaaaatcaagaaagagaaagaacaccgggcg

tggtggtgcacgcctttaatcccagcacttgggaggcagaggcaggcgga

tttctgagttcgaggccagcctggtctacaaagtgagttccaggacagcc

aggactatacagagaaaccctgtctcaaaaacatcaaaaaaaaaaaaaaa

agagaaagaacactgtctatgaatatataaagtaaaatacataaggcttt

aacggaacctaaaatacatgtcaaataatattttaaaaaccatttgtagg

ctgggtgcagtagcaaacatcaattgtcccttcatgtaggagactaaagt

aggattgctagtccagggctaccctgggctaaatgtaatgagtttggagc

tacatagtgaattggaggcaagcctggaccatatagcaagcccctgtctg

aaacaaaacaaaacaaaacaaataaaacatgtttaacacagtaacatgtg

tgatttcattgttatcctatgacctaacagggcatgattgaataactgtc

atatttttgtaggtgtggctttcttaaatgatacccccagagtggcatga

aaatagctacccatgacaaaggtgctccaggctgctttgcttctaacacc

cccagtccagagaactgcagactttcccagtgagaatcaagatgttctat

ttccccatccaagttcaccagcatcctgaatccttgccctgtcagctggg

gagtttcacaggcttctgtttaagaaggcttgctgagaggcgcggttctg

ccctgcctccaggggccagagcaggaggtgggggacgctgatgagtgggc

tgtggcgaatgaagccttcctcatgcccaacatcccaggagcccagccaa

caggaaaagttcccccattctccccgggaggccgccagcctgctgtctga

gaggggtggggccactgccctctagaagtgtgcctccttctggcagcaca

ctctccgccccctcccacccccttgtccaaaggcctcctacagaagggag

ccatatggccctttgtggacacattttccctggtacagtatcttcccggt

gtgaatttgggacctagtgccctagagtcagctgtctgtctcctccccaa

ctggcccccctccccacagcagcctaggggagaagcaagttgttgtgggg

acaagcccacggaatcagggagccctttggagtcggggtgagtgagaagt

gtctctgtggcacccacacccacgcccacacagaaaacagttgaagtaag

tcaaatgtctcaaagtcagatcctttttagtatgaatttgctgtgtctca

ttccgagcactaattccaatttaattgcaagtggcctgaagcctcttcaa

taggagtaacaaatggtttgattttgtaaacttcatcagactgcatttta

aatgcgacacagtagcccaactcgaatgccaatgaagcactggagtagct

ctctgtttactaattatccgtttttactccaagccacgctggctggcttg

tctgattaaatcggatttgtgggatagagagcagctagcagagttttctc

tatagtaaacgagattgaaactatgggccgcctggctgcacactgtaaat

tatatttccttttaaaggggacgtgtctcttttatttcaccgttgtcccc

ccctgcatattaaacttaacagcacaggaatggacccagcagcttctgga

tgtggcctctcagtctccacccgacaagagaggggaaaacaccgggaact

gttgtctgtgcccctctacctgtttcctctctaatacctttctttctccc

ctgtatactagcgttgagtcttcttctattattttttctttgtctgttat

tttcagaggtccttgagatgatatagatttgtactgatttagctgaaaga

gttctgtgataactgcatcagagtgatgttgggtttggtagcagtaagct

ggcacgtgtgcagagtccagccatacatttggtgactgtgggttcacctg

caacggaagatatttcctaaaaatgcaaataggttcttttggtacgtgct

gcaatggtaggtatccttcactctgaagcgagtgagataaagatcaccag

gcatgtaatacctgtggtccagcagattgggacatgtcgcagttatcagt

ggcgtgggcgtgttacaggttctcctatcctcccactgtctgtctggttc

tagtgactgtctgtccaagtgctctgcagcttgtttgtttcttcctgttt

ttttagggtagcaaatcagtacctgtctctggttcttccgtgtacatgtg

tacaagcacacacacaggcaggtgcgcacacacaggcaggtacacacaca

taggcaggcgcacacacgccaccgataattatgctggcacattgagtgtg

gtgtaatcagactatacttacactctgccatttctgagacttttggtccc

cagtccatccatgccccactctcggtgtctgtctctgtgcctggagcttt

ttatgtgttcatttgcttttgaatgtcctgcaacactaacgtgtatgctt

ttcccttttgggccattgtgtgctgtacagtacaagaagcctcacggtgt

ctctgatagcctggttaccgtgggaatgaatggcccgccatgctctcggg

cctacagtcctctttttaattgttgacagtaaaaagcatttaaatgctcc

ttaatttgtaatcatcttctgggggagaaagagaggaaccattaaaatgt

cagtaaaatgaatacattttctctttctcctgattttttttttttttttg

gttttggtttgtttatttacaggggatttttttttctcttcactgccagt

gtttgtcagatattgcaaatgctctgaaccaatcagtatttctcttactt

ctagtttctgtctcccctcttcctccccattctatgctatctctctgggg

ctctgttttgtcctgtctctttcctgtactcctgtattatgtttagcccc

cccccctggaaattggtcaccctacccccccttcacacacacacaaagat

tctacttctggaccccataaccctttctgggggcatctggtgccattatg

ggaactagagaaatggcttttgtgaaaaatgtctcgtgtacttgaagaac

tacctggaatcattgtctgtgccggtgtggaatccggtcccatgtttagc

tagaagccccatgctctctgccaagctctgaatgctgtactaagagagta

ggggcccactgtgcactgggacctggctaagaccagaggccactggatcc

agaggggtctgttcaccctgcccctgtgtgctctgcatgcactttggtat

catcccgacgctaggatgtggcaccttctgtctcgactctgctgcattct

cagctatcagaaaagtcttgcctgactcagatggggttcagggatccata

tccctagccactcaggaacatcacaggcatggccagtctgaggattgact

tactaggcctcggggagtggttccccaagtttttcttgccttctcttgct

aaaagtttgcatctcacaggatccttacaagtacctaagacatggattgg

tccctagctgtacctagtcaatgtcaggcccatctctgctttgcctgtga

caagtggttcccactgaagcctccagaatcacttgaaggcacttggccct

gtcctccatgggcctagaacattggctatcagagatggacaaattcttca

agggtctttccttttcctttcattgtccccgtaaagagcctgaggcatct

agccaggagctcacagtgtgtccttgagccaggtaggccttcttgctgaa

gctggcatgactttctgttctgtatgtagcctcttcaatgggggagtcta

ctataaaggtcacatgagggatggagcaagagacaaaggctgggtgaata

gcctggcctggtctgtctcatgccatcctgactctccatcacacactttt

ggagcctctgttcactgaggtctcccaaaccagatgaattggactccaag

agaccaattctctggaagggccaccttacggagagcaacaggtcacccac

tgaccatagagattgcagacctgaagatcagcaagggaaatggacttaga

gaggagctgacttgccctagatgaaggctgtatgatctctgaggctctga

aaagccagagagttgacagtatgtctcgccttgaaggtttaccccagtag

aacaagcccaatagggaaaccgaaggatggtttttttcctccaggtcctc

aaagaggtttggcatgacatccaccagtaccaggctctctcccatccttc

ctccggattgactaggtcatctctgatagaatggagccccctagaagccc

ttgcttcacacctacagggctgggctggagtcaggctaggtaagctcttg

tgggctgcctgtttttcctcccagggctgttgaagttggtccaatttttc

aatcgtgggccaattagtttcacaaatgggggcccctgtggtgccagggc

acatttgttccgggcctgcacactgaccaggccccattgtcctgcttccc

acccccttgtgggcccattgtccccgatctccagcaggccctgcttactg

ttgatttatatgggcaggcgtcccgcgggctccaggcttccctgccacag

ctggccgaggtgcttaaggcctcagactccctgacctttgccccgtcctg

ctgtcattttgaaagttacagaccagagggctgcatacctctccggattt

actttccatccctaccccccccttcacaccctcttcccatttcccttcca

gctccccctgctctgactgtagaggttttaagttcagtttggaatgtctg

caagggtccagcaagtagcctcggaagccaaagaggacaggggctgctag

agcaggtgactctccttcgcaaggctgctgaggctgccgcttctatttag

ggaagggcgttctcatccccaggttcatgagttctgagacttatgtgcag

ttgggaggttcaaagtaacccacagggtaccagaatgtgtgatgtcattt

tgcccttggcatctcttgtcccgtcttggcaccctaggaaaggagacccg

gcgtctatgaaatgggctgtccacagtggctgacagcagagagcaggtct

gctcccattgccgtccatttctgctctcttgtaaaccaacaccccacccc

acacacctttcctctacctcccttctttgcttttccacccctctcacatt

ttctccccatcttgggcaggtttgttggagtgattgacaaaattagtaaa

tccattcatgttacttttattgtgcaggtctcaataaatcccgagtgtcc

ggggcgcaccttaatgagctagtgagctgacaaatttgtttactgtagct

ggaggtgcctggtaatgaaatgcgcttgtgtctgggctcactgctaaaca

aatacacggtttccgggagggaggcagcaggctctgctagctgcagcagc

ctgaggaagcagccacattccccaagagactgacttgggtgtgtgcttgt

gttttgaaaaatgcagccactcagttttaaaacaaatttcaatcagctgc

ctgctcctcgtttatgttttccattgaggaatttctattcctttaacaaa

taccaaaccctggtaagattcttccttcccatcacataaatgatggaggg

ttagagagatgtgtagatacctcctaaggctctaaaaagaaaaaaagaga

gggaataagggctggaagagcctggagattcacagggttgaaacagacag

gaaggctaaggtttggggagaagtgactgtgtctgagacaaactcctgtg

caggagcttgacgctaactcagtggcaaggctttgagatgttttcccagg

ctgtggggtgagcaggataggcagagagtggctgggagggactgagctct

tccttgttggctgctgaagttaccaaagggtcaagcgggctgaaatgtgc

ctcagctgggtggattggattcttgtgccaagggattctgccactcaaca

cccagagagaaattaaagatgtaaactggcagcatgtgggccttcttcca

gggcatttctgagtcttcagcgatggttcaggagaagacaaagggtagtt

ccgcagatgtgagggtctgagcattcgtgtctgttctgctgcccactgca

tgcatccacaccccacaggcttttccagcctcttaagatgcagacagagc

cttctgcctttactgcgagtcagctgtgctgcctgtatcggtctctggca

cagtgggagctgcatctctgtgtcctcttcaacagcaacctggagggaca

tacctggtagagaaagccgagggagtaagagtagaggtatcttagggaag

ataatcccgaggggtcagcagcagcaggagagacctgcttgcagggttag

aggaagggagtgaaggtagaggtgtttccagttgaaattgaggtgttttg

cttagccccaatttgatatgttcttattgaggagacagctgctcggggta

gtaggagagatggctctcttccttggtgagtgggattggacaccagaaac

ccatctgtacacacacacaccgtacacgccccactcagagcagtcccagc

ctgcttacccaaagaaacagaagtatatttagcatcctctgctttgatta

aggaactgcctgtcgagggagataataagagccttgctccaagaaagtgt

accacctgagcgcctccaccagctgcagaggtgcccctcggatggcattc

cttccttttcattcaagtgctaattggcatggaagtggcacaggtgtaca

caggaggcttttgtgtcttacacctcccgaccctatcatcacctggacaa

gataaaggcccgagatccgtgtcatccctcctgcctcctgttctatctac

cctggcctggcttcctcccagtttttgctaaaattaaactcgtttttaag

tcaggaacctgtaagctgtacctgtgttccaccccagactggctgtgcag

ctccggtgtggctcactctctcctgtgatagtgacctggcgcagctccac

gctgctgcccacagcatcttcagttcttctcagagccaagagtttcaact

gtactgcagagaagaatttcagaatcaatccatgggaagccaacttggag

tttctgaaggaaagattttagcgtagattcaagtgtgagggttaacggaa

agaggaatgcttagagaaaggggcctacccactcaagtgggcgtgtgagc

attgaacttagatgtttcagtgatgtattggatatttggtatcttttgag

gttgtgccccacaaatgagattgtaggttgacttcttaggtgcactgggc

agtatttataatctgatcaggtaaaaccatggatcacagaggcttcacag

gggagcttggacgtgtctttccctgaaaatggagttctaatcttgcttga

gagacacctagagaattgctggcttgcagttgggaaagggaaaaggccat

ggctggtcatacacacataagcatggttcttatttgaaatatatcatgta

aaaggaattagcatgtagggcaacattttcagcaaatggtgctgcaattc

tttctccttaaatgtttgtgtgtctgtttgcatgtgtgtcagtttgtcac

gtgtgtgcctagtgcctgtggaggctagagaaagatgtcgaatcatctgc

aactggagttactgatggttgtaaaccaacatgggtacagagaaccagtc

tggatcctctgcaaatagaacaagtgttcttaaccactgagccatctttc

cagtcccctgtgctacaactattgactctcagccagcaagagacttaact

agattcatgagttgagatctatgggtagagacttctgtttctcccctacc

cacttccccttcttttctacttcaggaggaagtagaaaaagcacaggcaa

ttgcatggagcctacctatgtttcctcaactcacattctaaaggagtccc

ttgggcaaatacttggggaatctagaccaagccacatctcccccactctc

cagaaacatgggccttggggtggttgggtccttttcctgatgatgtagtt

ctctttgggccgaggctgagttttgtatgtgattcataatagaattccca

gattggaagaagacattgatgtgtggcttttatacactctgcagtgtagt

cagtcacctcgaggcaacatgaggaagcgagtgttagaagcagcaggttg

gttaggagttctgtgacttcagtgcagaggctcaggtcagggactgagat

ggtcagctttctgttactgtaacaaacaaccgagagagtgaacctgtgga

tgggaaaggttgggtttgcttcacagtccatggtcccttgttgcccttag

gcttgtgatgatgtggcattcctggcaaggagcttgagaaggaccaaaac

tctcacctctcacctctctcctctcacctcctggccaggcagtaagagag

acaacagggtggaaactccatcactcagggataccctacagtggtttgaa

gactttattcctagagtccaccttaaagtttccgccacttcccaacaaga

agttggacacaagagaacatttctgacccagtctacagctatggaaagga

cagtagttgcctgtccagggatcacaaggccaagaacagctacactgggc

tgtagtggggggtttgcactgttttgccatacagtgtcgagtgactgctt

cagtgtagttaattaactaagttcatgcctctaagcagcatagctcagac

gatggtgcagtcaacagacatggtaggagtgttactattgcttttatgaa

acatcacaaccaaaagctacttgggaggacacagtttctttcccccagac

ttctgtcatcatcaaaagcagtcagggcaggggctcgagcaggagaggaa

ccttggaagtaggagctgatgcagaggctgcagaagagtgctgcttactg

gcttgcctccatggctccaacagcctgtttgcttacagaatgcaggacct

ccagaccaggggtggccccatcctcatcaaccttgcccacagtcctttca

tatggaggcattttctcagtcaaggttcgctcttctttaatgactcttat

cttgtgtcaagtggacataaagctacccagcacagggagccagtgagttt

ggcatatgtgtgccacctggatttaatgattgccatggagataggattgt

gtggggaggctcttggtagtggccttgcttattatagacttagttctgag

ctctcaccagacctagctctgtaccatgacctactgaccaccatctgccc

tgcgtgcacacacttctgttatcaccccagagtccctctgtattgtcaca

gcacacacttaccaagggcctgcttgcgttcacccctgtgcggtgtgctg

gtgaggagaggtaggacggctcttctggttcctgctttggcagcttcgtc

tcgtaggccgagctgaaagttaaaagtaaacgcaataaagggttttatgt

gttgtaacagtgagtgctcagaagaagatggtgtccttggcaccaagtcc

tgactagtacaagtcatccaagatgccatggctagccggggtcagcttcc

tggaagaggcaacccagagtctataaagaagcatatgcttggtcgttgcg

caggggcttccaagagtcaccgtccgggttgttcactgcacaagggcgtc

cagctgagggagaggtggaggttaaaatgtagccccctctctagccaaca

gtgaggaaggaaacaggcttttctagtttggagaaaggcagcctttgggc

tggcatacagctaaccgctctgcgaagattctttcaagtgagtggggaat

ctcattggttgttcttagcaaccacagaaatatggaaaaggatggacaca

ctccaatccagggaagccaaatggcttacacgagatcccaaggcatttga

aatggcagaactagaatctccgctccgttttcctctctccgtctcacaga

ccgtgattgctaaagaccgtcttcatacagcccacctccttcctgatggg

agggacaaggtctggttcccatggctgcctcctcggtggaggatagaact

gaggcaaggcagggagcagagcacactagcgacgtgggtccctcgtccct

ctacacatctcatggcagctaatgaattgctaatacaattttctagataa

ttttatttccatagcccaggattttaatgtgtttccaaaagctagcattt

atttgcttaatctcccagtgtaatagattcgggcctgactcatcatacat

gcaaacaatcagctaggcagaaacgacagcaaattgtttaataactcaca

tctagtctttagatttttaacttgcgcgtaccaatagtttttcttgtgtc

tcatttcctcaaccctttcctgattttcttttgctttttcctttttttct

tttctaagtccttttctttcacagtgactgttggttttcaagctaaagag

tggagcaggtagattcttgcacctttaaacactcatctcaccccacaagc

ccaccactctttgagtcttggttggtgagtaagtggccaaaggaaagacc

ttgtgtttgtgtgagactccaaggagagactactgagtcataatatttct

ttttaactggcaattgtagaaccttaagccactcagatgcagagctggcc

atgtggggattttattttcccttttccctcctagagcccagctcctgcac

aaaacctctggcctcatcttgtctccaccttcctcctgtagtagcacaaa

agccagtcgttctctcccagcttccagccacttgagaccatccacgcctt

ggatgtgtatggaggtgctgcccactaggctgttggacacgtggcaccta

cctgtctcccgtcagtttgagttgctataacatggaaccatagactggat

gattcatatctagaaatgaatctctcccggtcctgaaggggtgggcagtc

tgagatggggattccagcacggctggcttcctgggatgcccttcttccta

gctgtagactgcagacttctgactgtgtcttgtgttctgaccttgaaaaa

ggatcttttcatcttgttgaggagggctccacccttgtaataaaagactt

tgttaagcctgaatctggtaataccatccccttgggggtaatgattttta

acatactcagttttgcagtggggaaggggacatctagacctttacagcac

tccagccctgtggccccgctttgcctgtgggatgccttccatctatgctg

tctgtcctctgtgatctgacctccccagtttgccaggcttgtctccatcc

tctgtgctacctcctctgaaatccgtttgcagctcactcttagccatttt

cttttacccctggcttctcagatgcaccccgtttctcggagctgaactca

aaatctctgaagctccgttggagccaccctttcttatctatgctctggat

ggtatttcttgaccaggcttggtatccagcttctctactccatctcagcc

tcttcttggtcacttggtaatggagtaagacaggtaaacggacacaaagc

aaattcaggccattctgtgagatgcaggtggcccaggagagcagcttcta

agcactttgggaatagcaaggggacaggatgccactcagtgtgctctgct

aggtggcatatttcagggtatcctcctggagtgacattcattcactgtcc

aagtccatactggggatctcagagtaagaattaaaaatgttggctggtag

aagcatgcctgttcaggaaacaagctcgctcggtctgagcacctagccta

gttaggctttctgtgggatagagttcagggtaaagcgggaataggtgctg

aaaacctatgacactggactgtggtgctgtgtgcattagaacttgtttgt

gggtggaatctgctcttgcttaccgggctggactggacactgcaggactc

agtgtcagagcttgtagctgcttctgcccaatggactacctctttcgtgg

atgtcctcctgcatcggatgggactctcttactggctccttcctgtggat

cttgggtttggaaagtttaagttacctgtgtccaatgacctgcctctgac

tgtgcttcccgggccatttctagagtgatcccagcatgctttggtgcttg

ctccttactgtctaggggaagcccagatagacagatatgtcccaaatgag

gttcagaagctaggaagtgactcagttgggtacactgactgatgagcaag

catgaggacttgcatttggatgcccagcactcacataacagccaggcatg

gcttcctgtgcctgtaatttccccctgcaggtaacagggacaagccaatc

ccacagctccctggccaactagtctacccaaaactacaagattcagtgaa

aggtcctgtcttaataaaatagatggagagccatcctgaggttaataacc

tctggccttcaaacaccccatcacacacgtacaccctacaaaggtacatg

gcacacatactaaagactctaaaataagtgggattcagttggtggtttct

acaatcaaatggaccaaggcattttctagattcccagctctacctctttc

tcactgtgtgaccttaggcaagttatttaatctctatttttctctttgga

agaaggtggcaacaatgcctgcccattagggatgcgtgttttaaatgaga

tgacagcacagataaagcacctagtggaaatggtggtctttagtgtcctc

tcccttctgctctcagacctttggaggacaccaggggttcttcctccgtg

cagccctcccagctgttggttgatgaagcaagcatgaccacaggtagcta

gaatgcagtgtctgcaaagagaggagccaacgggtaattgtgtggtggtc

cttgccacagctccttgcccaacattttggattccctatctctaacaaca

tcagctaccaacgcaagcccatatgcttttctctctgcccatatttgggt

ggcatggcctgtgctcagtcccacagtgctgtctcttgtgcaagtgacac

cagtggaaaagggaggtccttgccttttattccagaccaagctacatgtg

gtcctgaggaggcaagacccagccctcccatttgctgtagccattgctag

tcagggaggcagcaaaactcttcccgttcctttctctctcacctggcccg

aagagcttccttatgccccactgagcagcctgagactttcagtagcccaa

atgctctggtgctccttcctctttgcactcaactgtcagaccttgacctg

atcagctttgagttgagtctcaactggaaggctgtgtcaggaaggtgttg

agggatggaagatagcagcctgcctttgcgggcttcagtaggcacctagg

ctactgggccagccgagcaagtgaccaggtagaactgaatgaatatttca

tgtgactcactagttggaaatcctttaacaacttcttgttggtctgagga

tgaacccctgggctttgtagtatggctggtcaggacccacgtgatctggc

tcctggctcctggctcttctttctttgtatgttctccctgcttctaacgt

taggactggtcactgtgctcaacctggtgtggctctccctgaatgctcac

agactcggttccaatctccatgcactgggtgctgggtgctactgagatca

gaacagggactagtatactcttccccagcctttcatccccagaaccccag

agagagtctcccttctaggaagtatcacagtacaatagaatgaaatgact

ctcaccaatgcctgaatttttttttaagatgggagagtggttataagaat

atagtcaccagtggtggtggtggtggtagtggctcacacctttaatagca

gcgctcaggatgcagaagcaggcagatctttgagtttaaggccagccctt

gtctacagatagagttctaggacagccaggactacacagagaaatcctgt

ctagggggttggggtggggcactgatctagagctaacccaagggaataaa

tgaatgaatccttttactgctcccagttgggaatccaatggcaattgggg

tgggttaatctccccgtgtttctttctccttacatgaggtttgtggtttt

cagtgagttatgtcacaacagtggccagctcgtgtgatccaggttattgt

tggttcacacagaatttcccgtctccccactgctgcctcagactctgtaa

ggtccccgagttcactcccttagtcacctgcattgcgttcaaacctcttg

cagagatgcatctacgttaccatccacatggttttgtatctttctcccca

aacctttaggtctcgtggaggtcgggcacccgttggagctacattcaggg

tgctcacggactggactgtgggctttaggatctctagggggcagcgctgg

gtcttcaggggtgaaaatctggccttgggtctctccgttgctgaggctct

cccgttgctgtctcccacagGCACGGACATGGCTGTCTTCTGTCTGCTGT

GTGGGAAACGCTTTCAGGCACAAAGCGCACTCCAGCAGCACATGGAGGTC

CACGCAGGCGTGCGCAGCTATATTTGCAGTGAGTGCAACCGCACCTTCCC

CAGCCACACGGCTCTCAAGCGCCACCTTCGCTCACATACAGgtaggctgg

tccaggggatgggtggggagcatctctggaccttatctctgggcttcacc

tccaaaaatatatggtgtgtatgagtgcagggggagggtgtttcaggttg

cggcattctctgcttgaagttagacgcagaagggcttccatagaacattt

aaatgctgacagttcttccaatccaccacccacccctccacctgtagaca

gtctgttcccaatctttccacacatggcacagaaaataattcattaaaga

aaatcatactcttgaggtctatcctggctcagggtgagctctagatctca

acacaactaatgaatattcccaagggattctgacactggaggtgtgggga

ttgcaaattgcattttgagaaaaactgggaaaatcccacttttaccccaa

tcgatcctaggacaacactgaggtcagtcagcacagggctccagagggta

ctttgaggaactcaccctaccttcataacagacatgggaggcctctactg

tggccagaatgttgtctagggaagacagtgagagtatttgagcaagagcc

atgagatagccctgctcagccagttcacactatggagtgaaagacagggt

atgtgtgagtcaagtaggcagggctagttgcccggtcctagcctagtctt

gctaccaccctcttgatttaggggacaggatacccagctcagagggcctt

gaaggagctgtgggtcttttcctaactgaggagtagatgacttaactcct

ttctttgctctaaaacaaaatgagatcgtttcttactgtccatgagtgga

agaatgccaatttctgaattatctgtctgtctgtctgtctgtctgtctgt

ctgtctctccctccctctcccgtgtgtgtgtgtgtgtgtgtgtgtgtgtg

tgtgtgtgtgtgtgtgagagagagagagagagagagagagagagagaaag

agaaagagagagagagagagagctgggatcaagtctctctgtaaggatgt

agtggagtttgcacacagcataaatgttctgcactcttcagggagggctg

aaagaacttctcatcctcctagccttttcctagctgagggatggggtatg

tatgtggctggcccttacatgggtgacgggctctgatgaataaagaagga

gcttcactttcaggacctgagcagtacggtccagccagttgtcagcattc

tcaccaaggagcctgccttggctctgggttctggatgccaccctagtcag

aggagcacaatgccatatcattatcaaaggtgaaaggctcaccagatgct

tagaactacctagggctgcttggtagacaggagggggtcttagcaggtaa

ggtccagcaagggggaccaaagagagaggcgtatactcaacaactttctc

tacagcgtccgcataaggacctcccagtaagacatcatcccatcagtgat

tccctcatagaccccctaagttcagtctcagggaagatgaccagcttccg

ttagccacctccaggtatggcattggatcaagtcacttgatatcgcatgg

catgagtgactcaagccctacgaagtccacccaggatgcagcttactttg

cccatgcccctgtctcggagttcatttagccacgtgtcagtccaactcta

ctcagggcaagccaagcttcagatctccagcaaggacagagtttgggtga

cacccttgctcattctagaaacgcagacatgtgtgccccttgtgcacaca

cattcaagcactcattgattttcacaccagcccagcagaggctctgccca

cccctgacacaatgccttattaatattccgttgctcttggcccatttgct

gctggggcgggtgtttagcatcagcagctgtgtttggtgcgttagtccag

ccgtgctgttggcattgatggatccttgattcacatggctcatcatataa

taaactctcagctgcacggaagggaggggccgagaggacgggatctgagg

agcccggggccggggtggggaaggaggacggtggggttgaccggagcaga

aggaagccacatggtaatgacaaaccgggggaaaaggagggtcacaggtg

ccctgggcaggccagaggggctctaattagtctcgtttgtcctgcactta

atgtcaagctcattaacgcgcctacagagttaatcaacataccacaaatt

ggattttgacttttgtcgccagctttatgtacattgtggccagcctgaca

cgctcagctctcagggagagggttgtaggcctggtagccactgagctgta

aaacctgggtggggtgagagtaggtttgtgtcatagtgacttgtcatttt

tgcttttttttttttttattctctgaatgcttttcactctttccctgggc

agactccagggagttcctatttaaactcatttaacatgtgatccactttg

taattaaattcataaaaatctgatcaaacaaacccctccgttgtttgtta

aactcttagggcccccctctggaagccgggagcacacacaggcagccagc

cctccctcactgccaccccctcttcccagaggggggctgggtgactgccg

atgtacgttttaattaccggcttcggctgaagtctcacgtggcccttgct

ttatttatttatttatatcttaatgaggatactgaatctttgattaaagt

cgtaatcgttgctgattcaggaaggagggggagctgggtgtcagaccaga

cggggccatgtgtgcagacagacagacagaggagggagggagggcaggtg

gccagacagcagagagacagacagacagacagacagacaagagacaaaca

ggcaggcaggaagggagagaggaagagtggggagagagagcagcagacag

acaggcaggcaagacagcagacaggcagacagacagtcagacagcagaga

ggcagagagacaggcagacagagggggagggagggtaggtgggcagacaa

cagagagatagacagaaagacagagacaagcaggcaggcaggaagggaga

gagggaggaagggagggggagagaaagcgacaggcagacagacagacagg

caggcaggtagacagatggcagacagacaggcagacagacagacaggcag

gcaggtagacagatggcagagagacaggcaggcagacagacagcagagaa

aggcagagagacaggcaggcaagcatatggacagcagagaaacagagaga

cagagtctcatgcacaggctacccctcactatggtagtcctatagtcagt

cagtacctgtatcagtgagggctaacctcgtgcctttgatggggatagga

acagatccttggtatagctctagtagtgagagagacaagggttggtttgg

ccaggcctgtgagctacgacagcgccccttagatggtgaagactggtgag

ggatgggacctctctctgtgcttatgtcatgaagtcttgcccccccccgc

ccatgtcccatgttctttctgtatatctttcaccccacgagagagagtgc

cagtgaacagatcccaacaaccactccccacagagcctcagagaaatgtt

ctgagccatcagaggaggtgagaatttatgtgggtgctgggggggctgca

attcaggaagagggacttttggttagtggcccacagaggggagatggcag

agagagtaaaatatcattttaaagaggaagtgaaggcacagacatgcgag

tctataaggagaaggacaatttatgcccaggcacgagcacagtttgacat

ggggataatgaaaaaacgtaggtcattgtggatgacttaagccttcagca

gctgaagagaatgtatgaaatgcaggggacattacacggttggcagcctc

acacatctgcagagggacaagtgaaatacagtgggccattctttaccata

aactgtggttgttggagatgcagcccagagaaattgtaattacagtgaca

gaggaatcagcgatatttaaatattcatgaacaaagtctggggtgatcaa

tctatctttattgtctgcgccttcgctcctgtaataaataagtagctccg

cacctcacctttcattccccgagctcgacacctccgttacagaagtgcaa

ggtggataaccccactcctaccccttcggcccacgggaattgaaatgtgg

agtgtcccaagagaatggtcagctgatggacatgaggctagaccaaggaa

gggctgttcttcccagcccattacccagaattctccaggagttggttttg

agcctttcgtgggcaagcactgggggcataagggtgggaaaggctccatt

cttaccctggaggtgtttcttgtggccctgtcagtcagtacaatgcagtg

tggtgtcatttagccctgtggtaagtgcccagtgttcagatcctagaacc

agaccatagcagaagtcccaacctagctacttctagctgggtgaccttgg

gacagtggttaagtccctctataaacaactcattttccccctcatttgta

agataagcatatcttccatcagatggtttccattgccatgtgacacggtc

tctgtgatgctctcagatagcggaaaaaatacagcacacgtggaccatat

aaatgtcctatgttttggggggctggggttttttggtttgttttaagtcg

tgtttctgtaaataaaagactaaaaatgttaggtattcttctcgttgggg

atagagaagaaaacacagctcggtctgcctggggagctagaaggggagcg

agtgtgcacatgggggaggggtgatggatgatcagggatccttagggttt

gccagggaaaggagtagaagagaagcaggaaatacacttaactagtcaag

taggtgagagggtggtggataacactggggatcaaagagaagttctccaa

gctggagggattttttaaagggtaggaaagctaagctggagtggtccttg

gcccagaaccagaggaaatagagaagagttagtgaagcccctggctctgc

tccaccgacacaggggcctgcttcctcagggccattactctttgttggtc

caagcaggctttcttttcctgggactgacccttacccagggatgacagca

gagccacagtaagtctagatgtatgaacacagggactggctattcacgtt

gtgggatcagtcaggtggcgaatataggcaaactcaggaccctgctgtcc

cctgaaacaggcaggaggaagcaaggatcttatatccagtctattgttct

cacagGTGACCACCCATATGAGTGTGAGTTCTGCGGCAGCTGCTTCCGGG

ATGAGAGCACACTCAAGAGCCACAAGCGCATCCACACAGGGGAGAAGCCC

TATGAGTGTAATGGCTGTGGCAAGAAGTTCAGCCTCAAGCACCAGTTGGA

GACGCACTACAGGGTTCACACAGgtacttgagggccaggctggctctggg

acctgtatggtgactgggaataggtagttatttggagaagcccaagcaac

catggttcaaggctcaaccggagagaaaggggtctattaagagatcatat

ggggactagagagatggcttgatgaaaagggtgcttcctgtttggaccac

agtttggttcccagcacccacattggatgacccatgatcatctgtaacta

cagctccaggagatctgatgccctcttctggcctcagagggcaccacatt

catgcacacacatacacaaataaaaataatttttattttttaatttatat

ctttttatttttcaaggcagggtctgtgtagcccttggctgtcctggaac

tcattctgtagagcaggctggcctcaaactcacagagatccacttgcctc

tgcctcctgcatgctgggattgaaggcatgcaccaccaccattcgacttt

aaaaaagtattttttctaaaaaaaagagataatatgtggtctgagtacca

tcctcctcctctgaaggccaaggcagacattactaatcaattgtgacaga

taatagtaatcaattgcagtagtgtgtcagtcagtcctcctaggcaatca

tactataaaccatccaggagtatcaggcaatatgatgcttcttataaatc

ccaaattacagttaagttggtcagagtggggagaagtgaaatttgagtga

tttttatgttgctccattcacagttggaggcataatcagagggcattaag

ttatgataagggtcaggaagttgtgacattaattctggtgttctgaaatg

aaaggagccagtgtatttaatggtagaatgttccaccatgacagagtgct

tccacattcctttttacatagaaaaatcatgattctctgtcttttattgg

gggtctggggacagacagaggacagggtttggagttggagagagtagagc

cctaggaccggcctctacttaattggaaagcctatttcttccccattcta

agttcagttgcttaaaatgtgccccggtgggagtattgactggatagcat

tggagcatgtgtctttggagagctggttgttaaatacccaccccctgtag

tctgccagctgcggggaaggtagcattttctgtgaggctaagggtgaagt

agagcaaagaaagatggcctctctgatcagtggggacagcacccagtcca

gacaagggtatggtgacctcagctggcttaagaaagagaagagcccaggg

agaggcaggcaaccggcaggtgtaggcggcggagctcttctcagagctct

gtctcctggcctgagctctgtttttcttgcctctggcttaaagccacagg

agaagacagggtgcttatggctgacacgtgagtggcccaagggatatccc

agagtcccagggttccctcagtcccttgttgtgggatgtgacctagtggg

cagatcattaaatgcatcggcggtgactattgtaaatagctggtcgatag

ttagatcactttggctgttcattacttcattatttgctggggcctcagca

cggaggagtattgatagcgagatggctgaccagttaattactgtattgaa

acaaggttctcttacatctctgctcagctccaggggactctagtggaagg

gagatctggatagccggctagggtgacactgtcgtacactcctaggctct

ctgtactgcccctccgcactgtcctagactgggaggcaaagtccaggaga

ggtccttggagcattagtgggtagacagccctagatagagtatatttgaa

gaactggtttcttggatttcccctacctctcctccttactgactgtagct

ctaaaaagactgtatccattctcctgttccctgcgggtgagcttgttatg

ctgaagcacccagaagggcttcagggacttgctatccctttccacagcat

acacatttccttaattagaagggaagcaccctgccttaagatagtaagac

cagagcccagaacttcatggacagtcctctcgttaccagccactagccta

tttacagtagtcacaaaataaagtaggagtgaagtgccacaatgaccaga

ccactaggcctgtgtggctaacggctgccttgttcgatggtccagcccac

ggtcttgggaagttctgttgacgactgcagggctacaaggatggacattc

tccaacaagcccaaggatggctcaaggcccttcgggtcttcacattcccc

tgtcctgtcttgtcttttccacagGTGAGAAGCCCTTTGAGTGCAAACTC

TGCCACCAGCGCTCCCGAGACTACTCGGCCATGATCAAGCACCTGAGAAC

ACACAACGGTGCCTCGCCATACCAGTGTACCATCTGCACGGAATACTGCC

CCAGCCTCTCCTCCATGCAGAAACACATGAAGGGCCACAAGCCGGAGGAG

ATCCCACCTGACTGGAGGATAGAGAAGACGTACCTCTACCTGTGTTATGT

GTGAAGGGGGGGGCATCCCCCCCAACACATGGTAGAGCAGTGGCAGGAGC

CAGCAAAGGCGAGCTAGAACCAGACAAAGTGGAAAGACTACGGAGAAAAG

AATAACAGAAAAGAAAAGGGGGAAATAGAAAAAAGAGAGAAGGAGAAGGA

AGCCTGATAGTTACTTGGCCTTGGATTATTTCCTGGGGCTCCAGGTGATC

TGGCTATCAAACCACTGCCCCCTCCCTGGGGTCTCTGCCCTCCTTGGTTC

TCTTGGGTGGGTGGGGTGGGTTTGCTCATTCACACAGGGGCATATTTTTC

TCCTCTCCACAACCTATAACTTCGTTTTGTGTCTAGAAATGGAAACCAGG

AAGCAGAGGTGAGGGTCTCTCTGGTCAGAGCCATGACCTCTTAGACCTCC

TTTCCCCTCCTCTGAAGAGGATGGGAGGGGAGCAAAGAGGGAGAGGCAGA

GATGGGGGGGGGGTCCCCAGCTGATGCTCAGAGTGGCAGCTCTGGCGTCA

TGGCGGGAGAGGCTTTCTGCTCTGCCCAGCTACAGCTGGTGCCCCTCACT

GTCTCTGAGAGTCCAGTGGCTCTCCTCACCCTTCAGCTTTTTGCACCTGC

TTTTGATGGCTGTTCTCTGGTCACAAGGGGCTTACACCGTCTGTGGATCT

GAACTGTATCTTCCCTGGGTTCCTCCTAGGGAAAGCTGTGTCAGATTCCA

AAACTTCTTCCTTCCTTCCTTCCTTCCTTCCTTCCTTCCTTCCTTCCTTC

CTTCCTTCCTTCCTTCCTTCCTTCCTTCCTTCCTTCCCTTAGCTTTCAGA

GAACACAGAACAAAATAAAGCAGGCATTGTACCCCACCCAAACTCCCTCA

TGAGCAAGAGTCCTGCTGAGCTGCACTCTGCTTGCAAATGGAAACAAAGA

TCAAACAAAAACCAACTAAATATATTCTATCCCACCCTCCAAACAAGTAC

ACATCCCTGCAACCGCAGCTCAGCCCCTAGGGGAAGGAATCTCATCATCC

TATTGAAAGGCCCCTTTGCCCCAGATGGGGAGTGAATGCCCCCTCTCAGG

TGAGCTTTACGTGGTGGAGGTGGGAGTGGATGCCCTGCCTGATCAGAGTG

GGAGGATTGGCCACTGGAGAACGTTACTGTCACTGAGGATCCTCTAAGCA

CAGGGAGAACCCTAGATCTGGAGATTAGCACCGACTTCCTGGCTGGGAGT

GGAGGTGGTGGGGCTGCCCCCAGGGAGCAGAGGGGAGGAGCTGATGGCGC

TTCTGGCCTCTTGTCATGTTTGCTTTTAGCACATTGTACTTGAATTACCT

CAGTTTCTTTCTTTTTTTCTTTCTTTTTTTTTTTTGTGACCTCATGAGCT

TTTGAATCCCCTATCTCCTTCCCCACCCTTTTGGTTGTGAGAGATATTAG

AGGAACTGGGAGGGTGTCATTTTCTGTTCTAGTGGAAGTTAATAGTTGGC

TTTGAACTTGACCTGGTAGAGGCCCTCCCCTTCTCTGGCTGGAAGCTGTA

GGAAAAAGTGGACCATTCAGGATCTGAGTGGAGGCCAGGAGGGCTGAGAA

GGTGGTGGGAGTGGAGGGAAGGACGACTGGGTTGTCACTATGTCTCCCCA

TAGTATCACTAGTCACTGTTGGCACCACCCAGGGCTCAGACCATCAGTGC

CCAGCTTTAGTTCAGCCGGAAACACTCTGGAGAAAGGTCCCAGTGGTCTT

AGAATTATCTGGGCTGTTTAGGTTCTAGAGGGCCTGTTATTAAGATCTAT

ACAGCCACGTGGTCTCCTGCCGTTAATACTGCCAGAGTTGGTATATTTTC

ATGGCTGCTGTCTGTGTACATGAGGGGCAGATGAGCCAGGTTTTGTTGTG

CTGGGTGGGGTATTGTTTTTCTAAAAGCTACCTCTTCTGTTTGTCCAGTC

TTTGGTTCCTTCTGCAGCTCTGCCTCCTCCAGCCTCCCTGCCCTGAATTT

TGGGAAGCACATTTGTAATAATCTCCTTTGCTTTGGGACGGACCCACACC

CACTTCATTTCGTGCTTTATGTATGTGTAAGAGACCTGTTGTCTTTTCCT

CTCCTCCTCCTCTTTCTCTGAGAGAGAGTCATGGCTGTGTTTTGATCTCA

GATTTTAAAAAGTGGTTTAGGAAAAAACCGTTGACAGGGGCAAAGGGCCA

TGAAAAGGAACAGAAAGTCCGAGGGGTGGCCGCTGCTGTAGCCACGCCCC

CTATTCACCGGCCACGCCCCCATCCCGCCCATTTTTACCCCCTACAATTT

CATCCATCTTATCTGAAGGCCTTAAGAAAAAACATGTGTTGAGAGGATAT

GGGCTGGGGTCGCTTGACTCTCAGTGTGAGGTAGGGATGGTAAAGTGCAG

AGGGGACTATTTGGAACTGTAGGGATGACTTAGTGCATTGGGAAGGAAAA

AAAAACCCTTAAGCTTGAAATACGTGGCCAGAACAGCTGTTGTGCTTATA

ATGTGAAAAGAGGATCTTCTAGAAAAGAGTGGTCTTTAAAAGTCGTCATA

TACTATGGCTTATTCCCCTGACCAGGGGGATGAAGTGTGAACTGCTCACC

TTCAGGGATCTTTAACTCCCAGAAAACAGCATTTTTAACAGGAAGATGTC

ATTCAAGATCGGTGACAAAGAGAAAACCTTTCGACTCCTCCATAGAACTG

GCTGGTCCCTTGCCTCCCAACCCTTCCCACTCAGCCTGCCCCACACAGAC

CCCCGCCCAACCATAAAAGCTGGAGAGGGCTGAGCTGATCTTTTCAAACT

GTAATATTTTTGTAGTATTTGTTATTTCTTCCGTTCTACAGGACCTTGCT

CTTTTCCTTTTGTAATGTGAACAGGGGTGGGGGTGAAAAAGGAAAAAGT
