# Supplementary figures and images for: Loss of Zbtb32 in NOD mice does not significantly alter T cell responses
Source: F1000Res. 2018 Nov 5;7:318. Originally published 2018 Mar 14. [Version 2] doi: 10.12688/f1000research.13864.2 (PMC5909056; doi:10.12688/f1000research.13864.2)

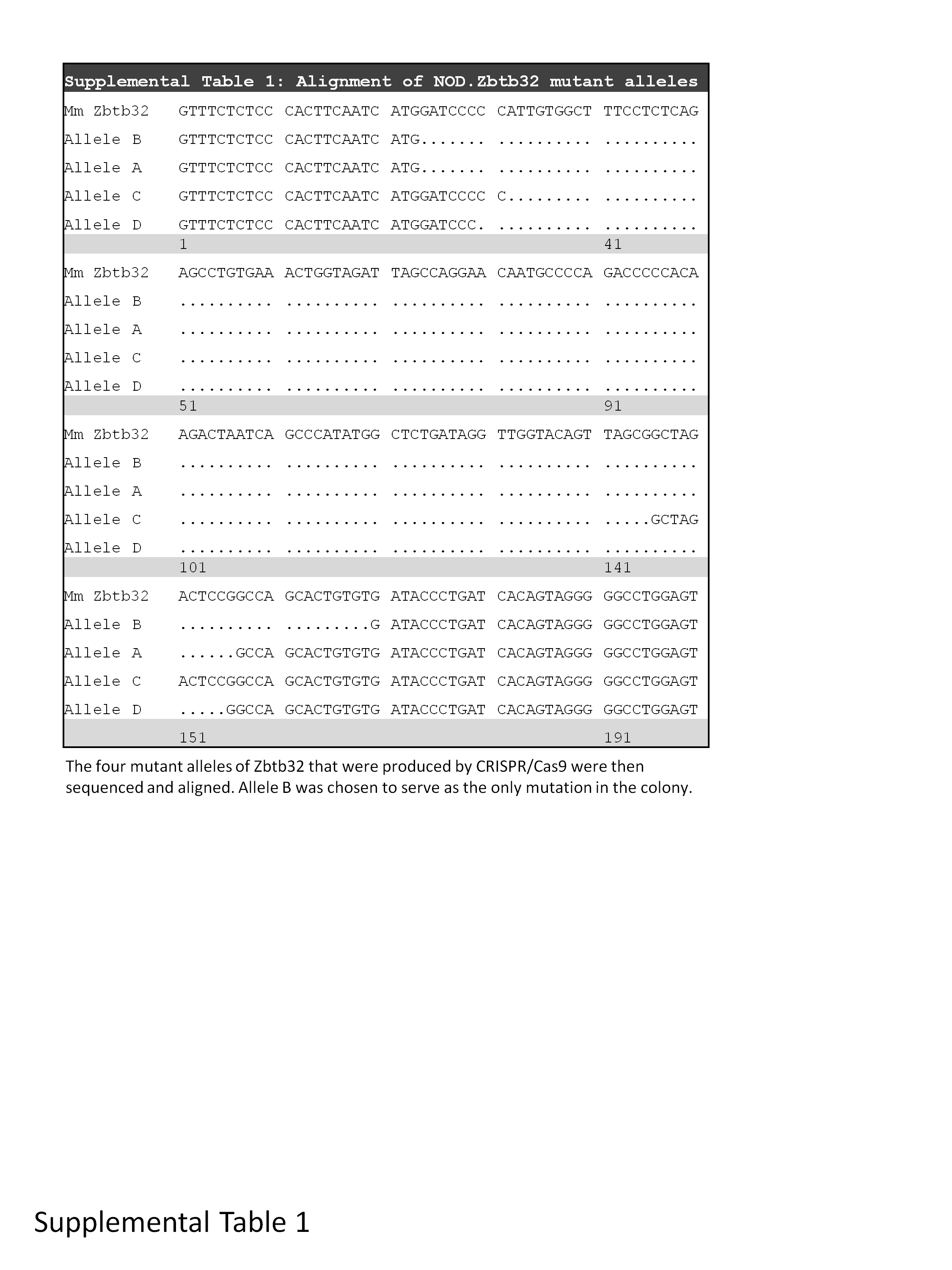

Supplement: Supplementary file 7 [file f1000research-7-18503-s0006.tgz › d48410df-b99f-4583-b507-b78f0d5033d5.TIF]

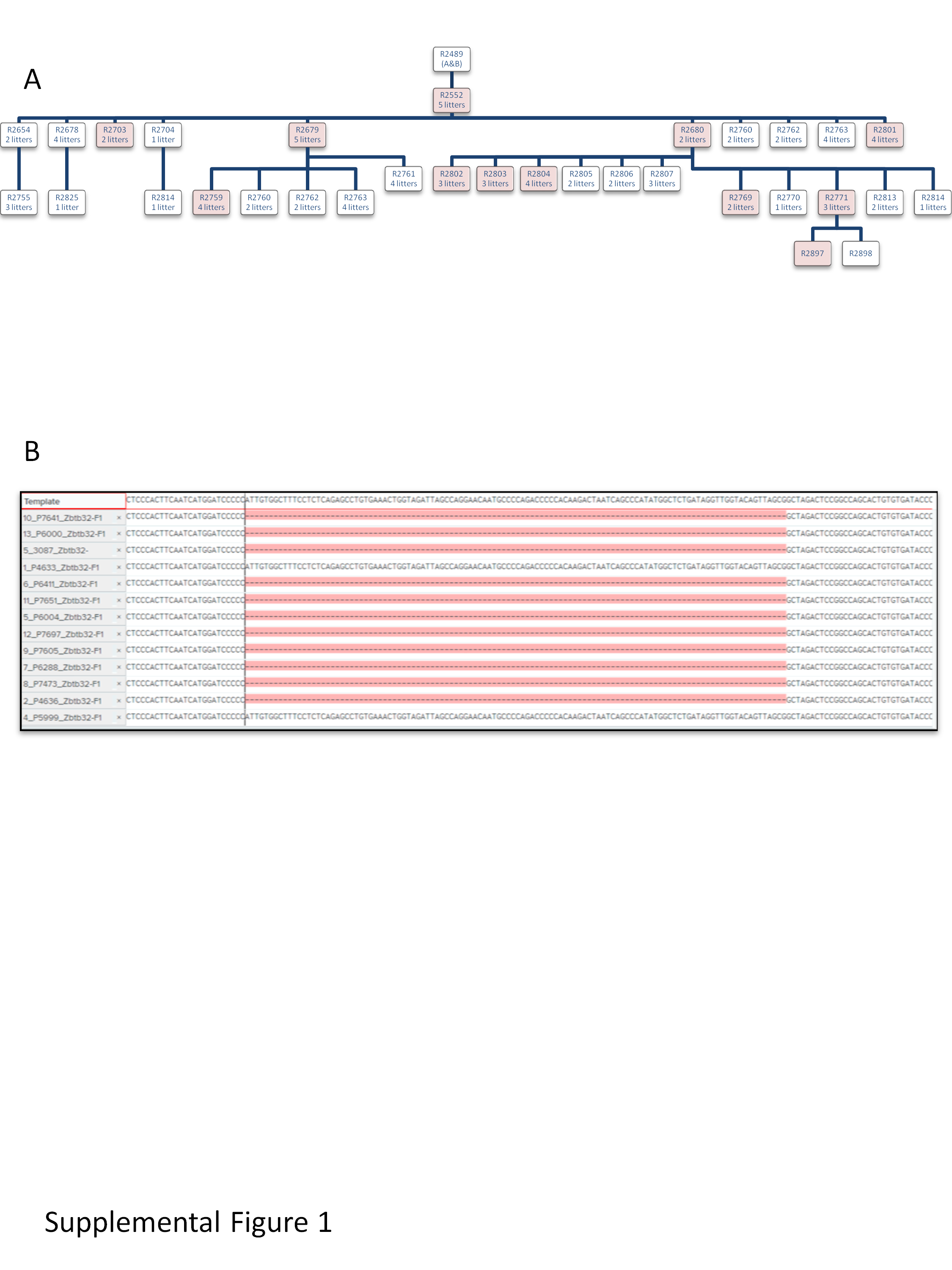

Supplement: Supplementary file 8 [file f1000research-7-18503-s0007.tgz › d95e5d97-4030-437c-95fc-cee38eb67b4b.PNG]

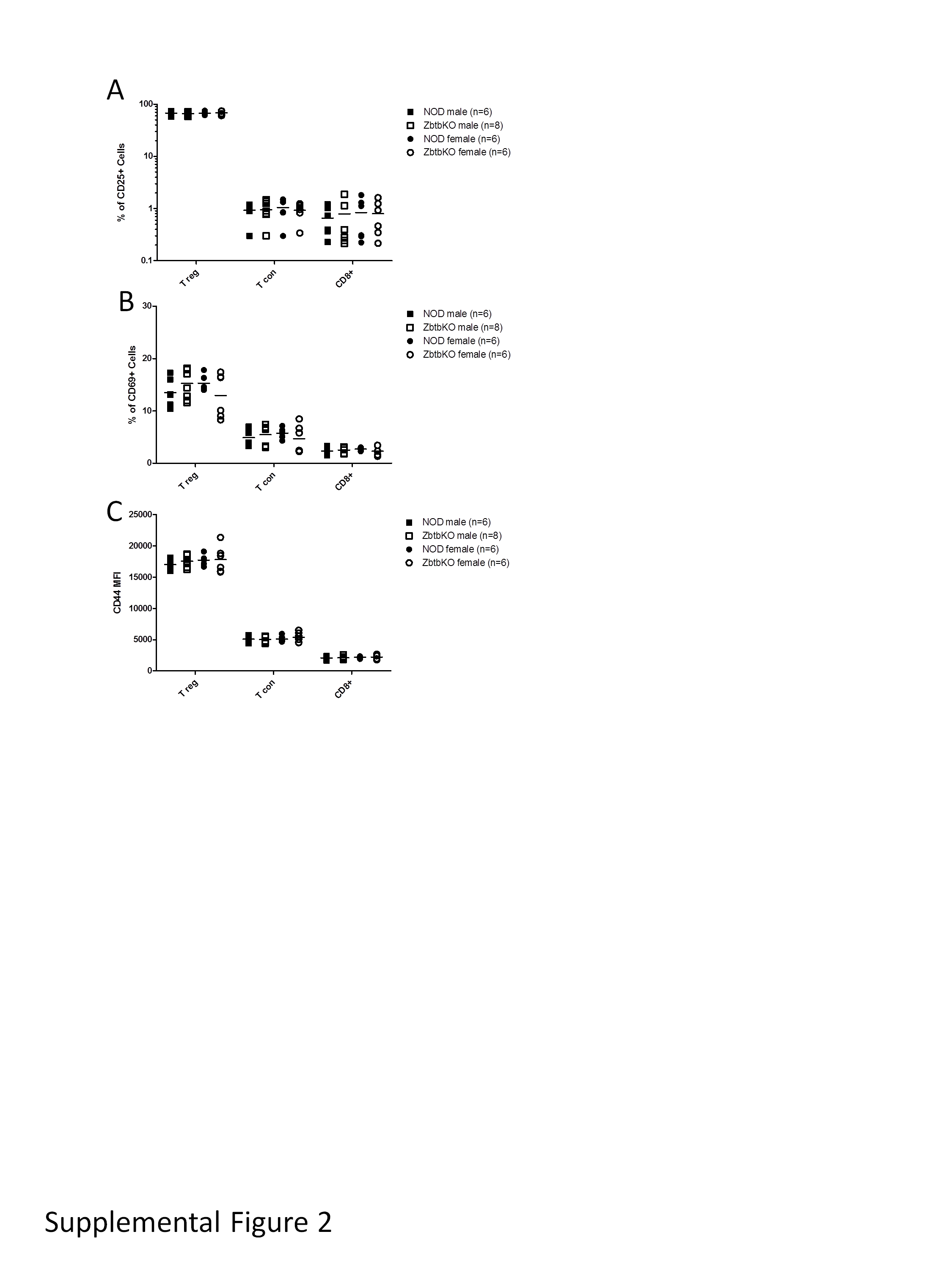

Supplement: Supplementary file 9 [file f1000research-7-18503-s0008.tgz › 3a522c55-398a-4d6e-8711-745d5a34c401.PNG]

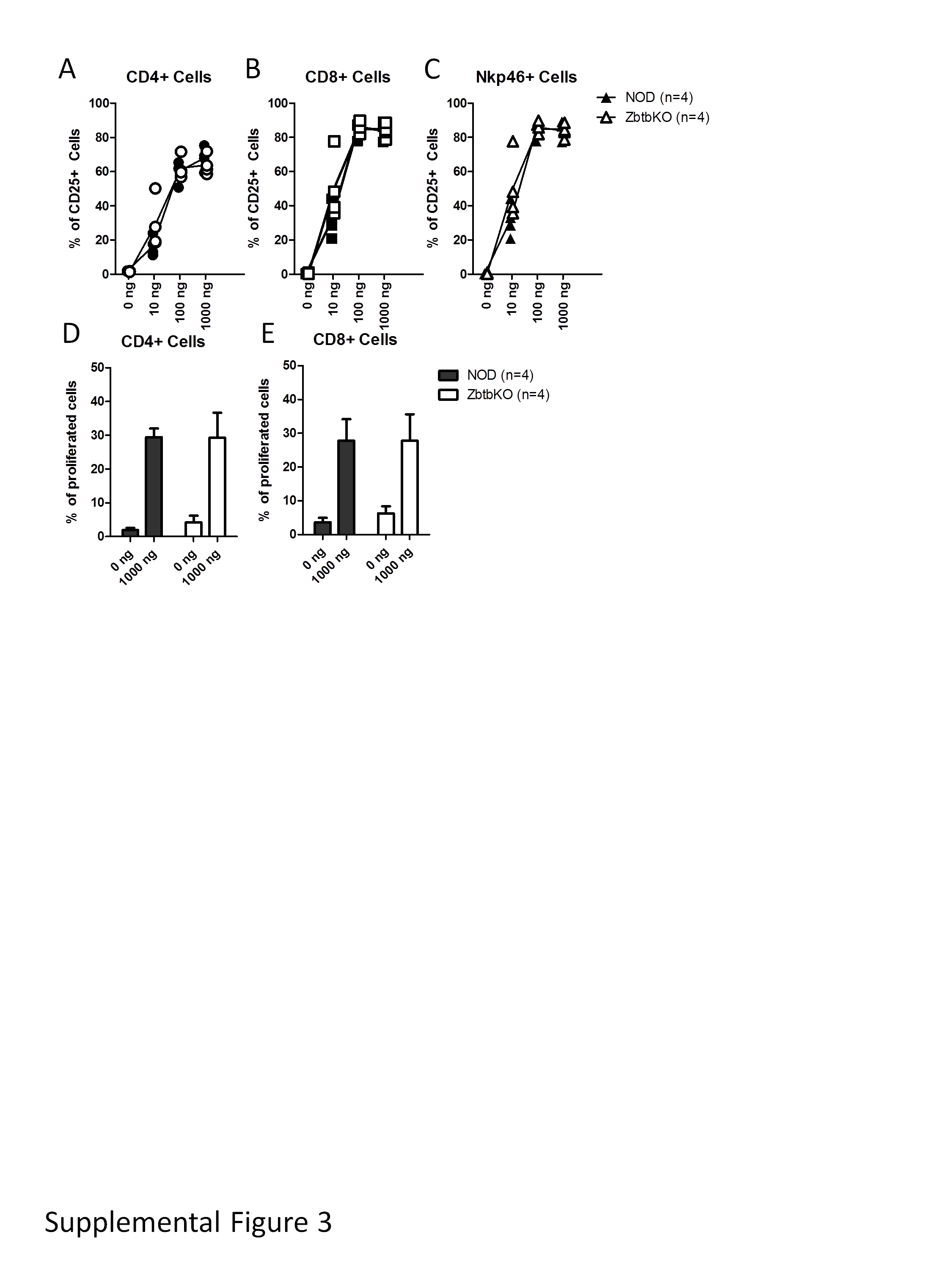

Supplement: Supplementary file 10 [file f1000research-7-18503-s0009.tgz › b7e7359e-3ec0-4c89-936b-e868d5ddb682.PNG]

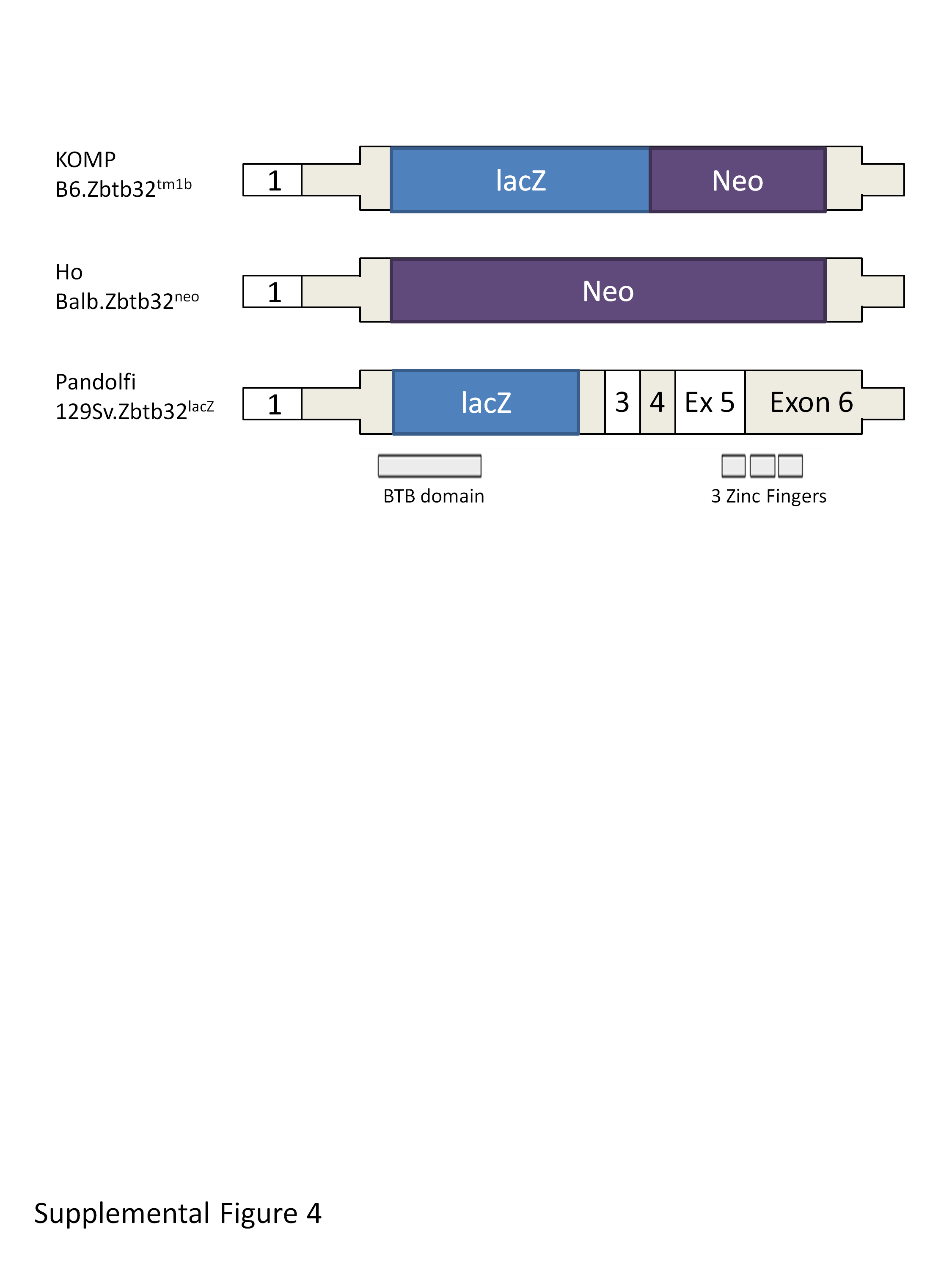

Supplement: Supplementary file 11 [file f1000research-7-18503-s0010.tgz › 5f8ee534-bf0f-4105-954f-8dd87bb33806.PNG]
