# Supplementary material for: Loss of Zbtb32 in NOD mice does not significantly alter T cell responses
Source: F1000Res. 2018 Nov 5;7:318. Originally published 2018 Mar 14. [Version 2] doi: 10.12688/f1000research.13864.2 (PMC5909056; doi:10.12688/f1000research.13864.2)
Supplement: Peer review history

# Peer review: Referee response for version 1

**DOI:** 10.5256/f1000research.15070.r32633

**Published:** 2018-04-19

**Authors:** Ivan C. Gerling

**Affiliations:** 1 Department of Medicine, University of Tennessee Health Science Center, Memphis, TN, 38104, USA

This is an interesting "negative results" report. The authors had previously reported data in support of the hypothesis that systemic loss of the expression of the gene Zbtb32 would increase T-cell activation and increase diabetic pathogenesis. The expected hypersensitivity of leukocytes was not observed. A minor but non-significant increase in diabetes incidence was observed in males, but only at later than 30 weeks of age. The lack of hyper-responsiveness of immune cells in these NOD mice (investigating an impressive panel of different leukocyte types and assays) is surprising since hyper-responsiveness had been found in 3 other strains with a knock out of Zbtb32. This raise an important question of whether immune responses and their regulation in NOD mice are different compared to other commonly used mice strains with respect to regulation by Zbtb32. However, as noted and discussed by the authors, the mutation on the NOD background was created using CRISPR/CAS9 whereas the 3 strains where the KO was successful in enhancing immune reactivity were more traditional KO mice. Furthermore the immune reactivity assays in this report are only comparing activation of cells from NOD mice without direct comparison to cells from the other strain backgrounds.  As such this question may warrant a more detailed investigation before it can be answered conclusively. The manuscript is clearly written and experimental designs are technically sound.

I have read this submission. I believe that I have an appropriate level of expertise to confirm that it is of an acceptable scientific standard.

# Peer review: Referee response for version 1

**DOI:** 10.5256/f1000research.15070.r31946

**Published:** 2018-04-09

**Authors:** Hubert M. Tse

**Affiliations:** 1 Comprehensive Diabetes Center, Department of Microbiology, School of Medicine, University of Alabama at Birmingham, Birmingham, AL, USA

Defining how tolerance is lost in Type 1 diabetes (T1D) will significantly contribute to our understanding of autoimmune dysregulation and the generation of beta-cell-specific effector T cell responses. Previous studies by the Tarbell lab demonstrated that DCIR2 ^+^ tolerogenic dendritic cells could promote self-tolerance to autoreactive CD4 T cells partly due to the upregulation of the transcription factor Zbtb32 in CD4 T cells. Overexpression of Zbtb32 in CD4 T cells was effective in delaying T1D by decreasing T cell proliferation and IFN-gamma synthesis. To further confirm the role of Zbtb32 in autoimmune diabetes, the current manuscript by Coley, et al. attempts to test the hypothesis that loss of Zbtb32 will exacerbate diabetogenic T cell responses and accelerate T1D in newly derived NOD.Zbtb32 ^-/-^ mice. Contrary to their hypothesis, the data presented displays a moderate acceleration in spontaneous diabetes in male NOD.Zbtb32 ^-/-^ mice, but no difference in female mice. In addition, there were no significant differences observed in activation markers, IFN-gamma synthesis, or proliferation between CD4, CD8, NK, and B cells in NOD and NOD.Zbtb32 ^-/-^ mice. The manuscript is well-written, results are interesting, and the conclusions are supported by the provided data. While the majority of the data is “negative”, the results are insightful and informative to the T1D research community. However, there are minor concerns that need to be addressed with the manuscript in its current form:

  

1. Zbtb32 is a negative regulator of GATA3, did the authors ever examine GATA3 expression levels and GATA3-dependent signaling pathways to determine if Th2 cytokine responses were elevated in NOD.Zbtb32 ^-/-^ mice?
2. The spontaneous incidence of T1D in female mice was stopped at 35 weeks of age, but the male incidence stopped at 45 weeks of age, if the female incidence was extended to the same time point, was there a difference?
3. I agree with the authors’ discussion concerning strain differences and potential compensatory mutations that may account for the absence of accelerated T1D in NOD.Zbtb32 ^-/-^ mice in contrast to other inbred mouse models. Out of curiosity, was EAE ever induced in NOD.Zbtb32 ^-/-^ mice to determine if this mouse may exhibit an increased susceptibility to ascending paralysis?
4. The figure legend for 4 needs to be consistent since the symbols are different for NOD and NOD.Zbtb32 ^-/-^ mice.

I have read this submission. I believe that I have an appropriate level of expertise to confirm that it is of an acceptable scientific standard.

# Response

**Published:** 2018-04-16

**Authors:** Kristin Tarbell

**Affiliations:** Department of Inflammation and Oncology, Amgen, Inc, USA

Thank you for taking the time to review our article and for your thoughtful responses. We truly appreciate your review and will endeavor to provide satisfactory responses to each the concerns that you raised.

**Query 1) Zbtb32 is a negative regulator of GATA3, did the authors ever examine GATA3 expression levels and GATA3-dependent signaling pathways to determine if Th2 cytokine responses were elevated in NOD.Zbtb32-/- mice?**

   Answer: It is our understanding that Zbtb32 normally inhibits the function of GATA3 by physically occupying the DNA binding site for GATA3, and not by regulating its gene expression. Consequently, we did not examine GATA3 gene expression. We did examine cytokine production in NOD.Zbtb32-/- lymphocytes (see Fig. 4), but did not observe any differences compared to control mice.

**Query 2) The spontaneous incidence of T1D in female mice was stopped at 35 weeks of age, but the male incidence stopped at 45 weeks of age, if the female incidence was extended to the same time point, was there a difference?**

   Answer: We monitored most of the female mice (50 out of 55) past 35 weeks but did not encounter any cases of disease onset in females beyond 31 weeks. The figure for disease incidence in females was truncated at 35 weeks for the sake of clarity.

**Query 3) I agree with the authors’ discussion concerning strain differences and potential compensatory mutations that may account for the absence of accelerated T1D in NOD.Zbtb32-/- mice in contrast to other inbred mouse models. Out of curiosity, was EAE ever induced in NOD.Zbtb32-/- mice to determine if this mouse may exhibit an increased susceptibility to ascending paralysis?**

   Answer: No, we never attempted to induce EAE in the **NOD.Zbtb32-/- mice.

**Query 4) The figure legend for 4 needs to be consistent since the symbols are different for NOD and NOD.Zbtb32-/- mice.**

   Answer: Thank you for the feedback. In Figure 4, data is shown for 3 cell types from both wild type and knockout animals. Each cell type was assigned its own symbol, but the coloration is consistent with the genotype. All wildtype data uses black, filled symbols; all knockout data uses open symbols. We will submit a revision to make this more clear to future readers.

Thank you again for your feedback and constructive criticism.

# Response

**Published:** 2018-04-17

**Authors:** Hubert Tse

**Affiliations:** University of Alabama at Birmingham, USA

Thank you for your responses!  I have no other concerns.
